# The immune system in cardiovascular diseases: from basic mechanisms to therapeutic implications

**DOI:** 10.1038/s41392-025-02220-z

**Published:** 2025-05-23

**Authors:** Xiaoyan Wang, Liming Chen, Jianming Wei, Hao Zheng, Ning Zhou, Xinjie Xu, Xin Deng, Tao Liu, Yunzeng Zou

**Affiliations:** 1 Shanghai Institute of Cardiovascular Diseases, Zhongshan Hospital and Institutes of Biomedical Sciences, Fudan University, Shanghai, China; 2 State Key Laboratory of Cardiovascular Diseases, Zhongshan Hospital, Fudan University, Shanghai, China; 3 Central Diagnostics Laboratory, University Medical Center Utrecht, University Utrecht, Utrecht, The Netherlands; 4 Jiangsu Provincial Key Laboratory of Critical Care Medicine and Department of Critical Care Medicine, School of Medicine, Zhongda Hospital, Southeast University, Nanjing, China; 5 Department of Cardiovascular Medicine, Anzhen Hospital Affiliated to Capital Medical University, Beijing, China; 6 State Key Laboratory of Cardiovascular Disease, Fuwai Hospital, National Center for Cardiovascular Diseases, Chinese Academy of Medical Sciences and Peking Union Medical College, Beijing, China; 7 Department of Biochemistry and Molecular Biology, School of Medicine, Southeast University, Jiangsu, Nanjing China; 8 State Key Laboratory of Respiratory Disease, Joint International Research Laboratory of Respiratory Health, National Clinical Research Center for Respiratory Disease, National Center for Respiratory Medicine, Department of Allergy and Clinical Immunology, Guangzhou Institute of Respiratory Health, the First Affiliated Hospital of Guangzhou Medical University, Guangzhou, Guangdong China; 9 Institutes of Advanced Medical Sciences and Huaihe Hospital, Henan University, Kaifeng, Henan China

**Keywords:** Cardiology, Inflammation, Translational research

## Abstract

Immune system plays a crucial role in the physiological and pathological regulation of the cardiovascular system. The exploration history and milestones of immune system in cardiovascular diseases (CVDs) have evolved from the initial discovery of chronic inflammation in atherosclerosis to large-scale clinical studies confirming the importance of anti-inflammatory therapy in treating CVDs. This progress has been facilitated by advancements in various technological approaches, including multi-omics analysis (single-cell sequencing, spatial transcriptome et al.) and significant improvements in immunotherapy techniques such as chimeric antigen receptor (CAR)-T cell therapy. Both innate and adaptive immunity holds a pivotal role in CVDs, involving Toll-like receptor (TLR) signaling pathway, nucleotide-binding oligomerization domain-containing proteins 1 and 2 (NOD1/2) signaling pathway, inflammasome signaling pathway, RNA and DNA sensing signaling pathway, as well as antibody-mediated and complement-dependent systems. Meanwhile, immune responses are simultaneously regulated by multi-level regulations in CVDs, including epigenetics (DNA, RNA, protein) and other key signaling pathways in CVDs, interactions among immune cells, and interactions between immune and cardiac or vascular cells. Remarkably, based on the progress in basic research on immune responses in the cardiovascular system, significant advancements have also been made in pre-clinical and clinical studies of immunotherapy. This review provides an overview of the role of immune system in the cardiovascular system, providing in-depth insights into the physiological and pathological regulation of immune responses in various CVDs, highlighting the impact of multi-level regulation of immune responses in CVDs. Finally, we also discuss pre-clinical and clinical strategies targeting the immune system and translational implications in CVDs.

## Introduction

Cardiovascular diseases (CVDs) remain the leading cause of global mortality and continue to be the primary contributor to the worldwide disease burden.^1–3^ The number of CVDs patients has doubled from 1990 to 2019, and the relevant mortality has increased by 5.5 million over that period.^1^ Meanwhile, global trends for disability-adjusted life years (DALYs) and years of life lost also increased significantly.^1^ Therefore, it is urgently necessary to elucidate the mechanisms of CVDs progression and, based on these mechanisms, develop new drugs to significantly reduce mortality.^3^

The immune system, essential for the host defending against pathogens, acts as a double-edged sword in the physiological and pathological processes of CVDs.^4,5^ Both the innate and adaptive immune systems play significant roles in this process.^6,7^ Immune cells such as macrophages, dendritic cells (DCs), T-cells, and B-cells, which are components of the immune system, are essential for maintaining vascular health and integrity.^8–10^ The multi-level regulatory signaling pathways and mechanisms of the immune regulation also matters in CVDs.^11–13^ In addition, there’s great progresses in therapeutic targets and clinical research progress (e.g., FDA-approved drugs and clinical trials) regarding the immune regulations in CVDs’ treatment in recent years.^14,15^

This review aims to illuminate the complex interplay between immune system and cardiovascular health. It offers a systematic exploration of the research achievement that have shaped our understanding of immune regulation in the cardiovascular system. Then the endotypes and immuno-features of CVDs are discussed in terms of both physiological and pathological. Also, the activation and regulation of immune responses in the context of CVDs are highlighted, including both innate and adaptive immune responses that contribute to CVDs progression. Furthermore, the multi-level regulatory signaling pathways and crosstalk between immune and non-immune cells in CVDs are discussed, highlighting the importance of epigenetic, post-transcriptional, and post-translational modifications in modulating immune responses. Additionally, the crosstalk of key signaling pathways, such as G protein-coupled receptor and growth factor receptor pathways, with immune responses are summarized. Importantly, the review outlines preclinical strategies and clinical progress in immune regulation for CVDs, covering potential therapeutic agents, FDA-approved drugs, and ongoing trials. This review will conclude with key insights, future research directions, and the role of immunology in CVD management, highlighting new opportunities for prevention and treatment. The systematic insights provided in this review aim to furnish a current and thorough understanding of the immune response in CVDs. This knowledge is expected to contribute significantly to the future development of the immune response in both basic research and clinical translation in CVDs.

## The past and present of immunology and immuno-therapy in CVDs

The role of immune response in atherosclerosis was first identified by Rudolf L.C. Virchow and Nikolay Nikolaevich Anichkov more than a century ago.^16^ However, for decades, research mainly focused on cholesterol’s central role rather than immune response in the development of human atherosclerosis.^17^ In 1985, researchers from Cambridge University confirmed Virchow and Anichkov’s earlier views by identifying macrophage foam cell clusters forming fatty streaks in human atherosclerotic plaques.^18^ That year, Jonasson et al. showed that major histocompatibility complex, class II, DR Alpha (HLA-DRA), nearly absent in normal arterial walls, was significantly expressed in both immune cells and vascular smooth muscle cells (VSMCs) within atherosclerotic plaques.^19^ Later evidence identified monocytes, T cells as well as macrophages in these plaques, reinforcing the idea that dysregulated immune response contributes to atherosclerosis development.^20–22^ In 1977, Andreas Grüntzig developed primary percutaneous coronary intervention (PPCI), an effective treatment for preserving viable myocardium and limiting infarct size following an acute myocardial infarction (AMI).^23,24^ However, myocardial reperfusion can cause additional death of previously viable cardiac myocytes, known as myocardial reperfusion injury, which can contribute up to 50% of the final infarct size.^25^ By the 1980s, researchers identified vascular immune dysregulation in coronary arteries before myocardial infarction (MI) and cardiac inflammation after MI. Furthermore, ischemia-reperfusion injury (IRI) is partly caused by a burst of oxygen free radicals, which leads to lipid peroxidation and membrane damage, with neutrophils as a potential source of these free radicals.^26–28^ While most pre-clinical studies focus on biological processes induced by AMI, Sarah A. Dick and Slava Epelman highlighted that the balance between physiological and pathological immune dysregulation also influences the progression of chronic heart failure (HF).^29^ In the 1990s, researchers found that low-grade chronic inflammation might contribute to clinical deterioration in patients with non-ischemic heart failure.^30,31^ Moreover, HF, whether ischemic or non-ischemic, is frequently linked to increased plasma levels of pro-inflammatory cytokines like tumor necrosis factor- alpha (TNF-α) and soluble TNF-α receptor, which are associated with worse clinical outcomes.^32^ In 1994, Attilio Maseris, a prominent cardiologist in ischemic heart disease, made significant contributions to understanding the inflammatory pathogenesis of unstable angina. His group discovered that elevated acute-phase reactants, like C-reactive protein (CRP) and serum amyloid A (SAA), predicted poor outcomes in unstable angina patients.^33^ This observation highlighted the role of immune system in unstable angina, and the findings were soon expanded upon by another research team. Paul Ridker et al. discovered that prediction models combining inflammatory markers (hs-CRP, SAA, interleukin-6, soluble intercellular adhesion molecule-1 (sICAM-1)) along with lipid levels more accurately predicted cardiovascular risk than models based solely on lipids.^34,35^ These studies established pro-inflammatory cytokines as key prognostic indicators by linking dysregulated immune response to increased cardiovascular risk. Later that year, Paul Ridker and colleagues found that elevated plasma TNF-α in post-MI patients was linked to a higher risk of recurrent coronary events and was predictive of CVDs prognosis.^36^ In 2003, Roman et al. reported that patients with chronic inflammatory diseases have an increased prevalence of underlying atherosclerosis compared with healthy controls, independent of traditional risk factors, indicating atherogenesis is associated with systemic inflammation that occurs prematurely.^37^ In 2005, lymphoid follicle-like structures in the aged aorta of *Apoe*^−/−^ mice, now known as tertiary lymphoid organs (TLOs), were characterized by the aggregation of T and B cells.^38^ Furthermore, in 2015, Andreas’ group elucidated the protective role of TLOs against atherosclerosis progression.^39^ Of note, recent advances in single-cell technologies, such as single-cell mass cytometry, cellular indexing of transcriptomes and epitopes by sequencing, and single-cell RNA sequencing (scRNA-seq), have significantly enhanced our understanding of immune and non-immune cell interactions in atherosclerotic tissue, marking a major leap in studying immune heterogeneity.^10,40^ In 2023, Sun et al. found TLOs present in various CVDs and used 28 single-cell RNA sequencing datasets to investigate their formation and heterogeneity.^41^ Also, Rafael Kramann’s group utilized single-cell spatially resolved transcriptomics to map gene regulation and cardiac remodeling in human tissue post-MI.^42^ Additionally, immunotherapies for cancer, like immune checkpoint inhibitors (ICIs), which boost immune surveillance against tumors and are increasingly used in various cancers, have been associated with cardiovascular events.^43^ The CAR-T cells in vivo by delivering modified messenger RNA (mRNA) in T cell–targeted lipid nanoparticles (LNPs) could reduce fibrosis and restore cardiac function after injury.^15,44,45^

**Fig. 1:**
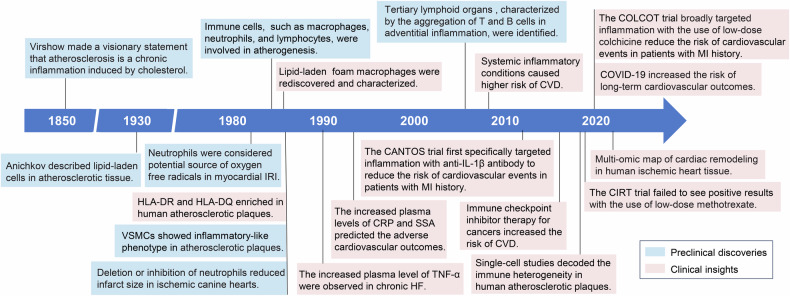
Timeline of key milestones in the development of immunology and immuno-therapy in CVDs in cardiovascular diseases. IRI ischemia-reperfusion injury, HLA-DR Major Histocompatibility Complex, Class II, DR, HLA-DQ Major Histocompatibility Complex, Class II, DQ, VSMCs vascular smooth muscle cells, TLOs tertiary lymphoid organs, CVDs cardiovascular diseases; CANTOS the Canakinumab Anti-inflammatory Thrombosis Outcome Study (CANTOS), CRP C-reactive protein, SSA serum amyloid A, TNF-α tumor necrosis factor-α, HF heart failure, COLCOT the Colchicine Cardiovascular Outcomes Trial, COVID-19 Coronavirus Disease 2019, CIRT the Cardiovascular Inflammation Reduction Trial

Building on these significant discoveries, numerous clinical trials are currently underway to explore and validate new therapeutic strategies. In 2017, Ridker et al. reported that administering 150 mg of canakinumab every three months significantly lowered the risk of recurrent cardiovascular events compared to placebo in the Canakinumab Anti-inflammatory Thrombosis Outcome Study (CANTOS).^46^ The CANTOS study was groundbreaking, being the first large-scale trial to show that targeting interleukin-1β (IL-1β) with anti-inflammatory treatment significantly reduces cardiovascular events in coronary heart disease patients. In 2019, the Colchicine Cardiovascular Outcomes Trial (COLCOT) underscored the role of inflammation in coronary atherosclerosis by demonstrating that low-dose colchicine reduces cardiovascular events in patients with a history of myocardial infarction.^47^ Overall, these clinical trials emphasize the importance of targeting the immune system in individuals with residual inflammatory risk, highlighting the potential of immune-targeted therapies to improve cardiovascular health (Fig. 1).

## Immuno-features in cardiovascular system under physiological conditions

**Fig. 2:**
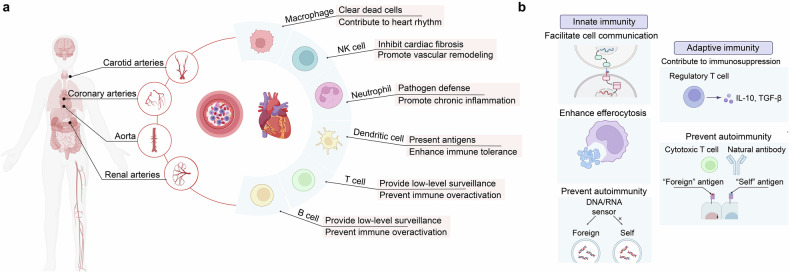
Immuno-features in cardiovascular system under physiological conditions. **a** Overview of Immune cell in cardiovascular system; **b** Innate and adaptive Immune Signaling Pathways in Cardiovascular Homeostasis (Created with BioRender.com, https://BioRender.com/i19e947)

Cardiovascular homeostasis relies on the precise coordination of immune cells, signaling pathways, and cell interactions to maintain an inflammation-free environment essential for optimal cardiac function.^48^ The immune system’s role in the heart under physiological conditions involves finely tuned responses from various immune cell types that contribute to tissue repair, maintenance, and immune surveillance without triggering an inflammatory cascade^48^ (Fig. 2).

### Overview of immune cell in cardiovascular system

Innate and adaptive immune cells play crucial roles in both the homeostasis and pathogenesis of the cardiovascular system.^49,50^ The main types of innate immune cells involved in cardiovascular diseases (CVDs) include neutrophils, monocytes/macrophages, eosinophils, dendritic cells (DCs), mast cells, natural killer cells and innate lymphoid cells.^49,51^ The adaptive immune cells include CD4 + T_tem, such as effector-memory CD4 + T cells, CD4 + T_cytox, CD4+ cytotoxic T cells, CD8 + T_tem, CD8+ effector-memory T cells, CD8 + T_cytox, CD8+ cytotoxic T cells, and B cells, such as marginal zone B cells, and regulatory B cells (Bregs).^12,49,50,52^ The origin of immune cells in the cardiovascular system is diverse.^53–57^Innate immune cells, like tissue-resident macrophages (C-C chemokine receptor type 2-, CCR2- macrophages), may develop from the yolk sac and fetal liver progenitors,^8,53,54,57^ while CCR2+ macrophages are derived from the recruited monocyte.^8^ Others are continuously replenished from bone marrow-derived progenitors.^58–63^ Among the overall cell population, the largest cell populations are macrophages/monocytes and B cells, followed by Natural Killer cells (NK cells).^12^

#### Macrophages phagocytose bacteria, clear dead cells, and contribute to cardiac rhythm

They are abundant within cardiac tissue and serve various functions, including phagocytosing bacteria and clearing dead cells, which help prevent tissue inflammation and support cell turnover.^8,48,64^ Notably, macrophages form direct connections with cardiomyocytes via connexin 43 (Cx43) gap junctions, enabling electrical coupling that modulates cardiomyocyte activity and contributes to cardiac rhythm maintenance without promoting inflammation.^65,66^ Moreover, macrophages appear to play a role in cardiac renewal. Under normal conditions, macrophages remain in an anti-inflammatory state but can shift to a pro-inflammatory phenotype in^12^ response to minor injuries, facilitating repair and clearance of damaged tissue without excessive inflammatory responses.^64^

#### NK cells inhibit cardiac fibrosis and promote vessel remodeling

NK cells support immune regulation by controlling the extent of inflammation, preventing immune cell over-accumulation in cardiac tissue, and thus preserving cardiac stability.^48,67^ NK cells play a critical role in preventing cardiac fibrosis by directly limiting collagen production in cardiac fibroblasts and curbing the buildup of specific inflammatory populations and profibrotic cell types, such as eosinophils, within cardiac tissue.^68^ Upon activation through IL-2 administration, NK cells facilitate blood vessel remodeling via α4β7 integrin and killer lectin-like receptor subfamily G member 1 (KLRG1), independent of their involvement in initial vascular formation.^69^ Activated NK cells initially adhere to cardiac epithelial cells (CECs) through α4β7 integrin and vascular cell adhesion molecule 1 (VCAM-1), disrupting N-cadherin bonds via KLRG1.^69^ This interaction translocates β-catenin from the cytoplasm to the nucleus, alleviating contact inhibition and promoting cellular proliferation.^69^

#### Neutrophils prevent chronic inflammation and infections

Neutrophils contribute to cardiovascular health by performing tissue surveillance, patrolling the vascular endothelium, and identifying potential pathogens or tissue damage, which helps maintain cardiac integrity and prevent infections.^70,71^ Under normal conditions, neutrophils produce reactive oxygen species (ROS) in controlled amounts, essential for pathogen defense and promoting cellular repair, with regulated ROS release avoiding oxidative stress.^72,73^ Additionally, neutrophils release proteolytic enzymes, including elastase and matrix metalloproteinases (MMPs), which facilitate extracellular matrix remodeling and vascular adaptability.^74^ Through vascular endothelial growth factor (VEGF) release, neutrophils promote angiogenesis, supporting the formation and maintenance of blood vessels crucial for oxygen supply in metabolically active cardiac tissue.^75^ By signaling macrophages to phagocytize apoptotic cells, neutrophils help reduce inflammation, prevent unnecessary immune activation, and maintain cardiac immune balance.^76^ Overall, neutrophils play a supportive role in cardiac function by balancing immune responses, preventing chronic inflammation, and promoting tissue integrity.

#### DCs present antigens and enhance immune tolerance

DCs in cardiac tissue serve as antigen-sensing sentinels, continuously surveying for foreign antigens or cellular abnormalities, thus preventing infections while maintaining tolerance to self-antigens to avoid autoimmunity.^77^ They regulate local inflammation by presenting antigens to T cells and activating anti-inflammatory pathways, which minimize immune activation that could harm cardiac tissue.^78^ DCs also monitor endothelial health by detecting changes in the endothelial environment, supporting vascular integrity, and facilitating the clearance of apoptotic cells, thus preventing inflammatory responses caused by cellular debris and contributing to overall cardiac stability.^79^

#### Regulatory T/B cells prevent excessive immune activation while effector T/B cells provide low-level surveillance

By secreting anti-inflammatory cytokines like IL-10, Regulatory T cells (Tregs) prevent excessive immune activation that could lead to inflammation in cardiac tissue. This helps prevent autoimmunity and chronic inflammation within the heart, maintaining a balanced immune environment.^80^ While Tregs aid in immunosuppression, effector T cells provide low-level surveillance, ensuring any damaged or abnormal cells within the cardiovascular system are promptly recognized and, if necessary, cleared.^81^ B cells contribute to normal cardiac function by maintaining immune homeostasis within the cardiac environment. Bregs play a critical role in this process by producing anti-inflammatory cytokines, such as IL-10, which prevent excessive inflammation that could disrupt normal cardiac function.^82^ Furthermore, B cells produce antibodies that identify and neutralize pathogens in the bloodstream, helping to prevent infections that could indirectly affect the heart by triggering systemic immune responses.^83,84^ In addition, B cells regulate autoimmune responses by controlling antibody diversity, limiting self-reactive antibodies that could target cardiac tissue and ensuring cardiac stability.^85^ Certain B cell subsets also release factors that support tissue repair, which is beneficial for minor myocardial injuries, enhancing cardiac resilience and structural integrity.^82,86,87^ Moreover, B cells interact with macrophages and T cells in the cardiac environment to promote a balanced immune response, ensuring that immune reactions are proportional and supportive of cardiac health.^88^ Collectively, these functions allow B cells to play a multifaceted role in preventing unnecessary inflammation, controlling autoimmunity, providing immune surveillance, and supporting tissue integrity in the heart.)

### Innate and adaptive immune signaling pathways in cardiovascular homeostasis

#### Innate immune signaling pathways facilitate cell-to-cell communication, enhance efferocytosis, and prevent autoimmunity

Certain cells, such as resident macrophages and endocrine cells, respond to external stimuli or internal signals by producing and releasing chemokines, which act as messengers to convey information to neighboring cells. These bioactive mediators can orchestrate different cell types within a particular tissue, modulating a wide range of physiological processes, such as development, growth and renewal. Furthermore, nucleic acid-recognizing molecules, such as the DNA and RNA sensors are directly involved in regulating cardiovascular behaviors through interacting with other intracellular homeostatic processes, including apoptosis and autophagy, thereby regulating cardiometabolic health.^89^ This biological process largely depends on their ability to discriminate self-DNA/RNA from non-self DNA/RNA, suppressing uncontrolled autoimmune response.^90^ Importantly, incorrect self-DNA/RNA recognition could lead to the release of specific autoantibodies, indicating that DNA/RNA sensors serve as critical immune checkpoints and control the autoimmune responses.^91^ On the other hand, the activation of these immune pathways in phagocytes helps facilitate the resolution of apoptotic cells harboring damaged self-DNA/RNA following programmed cell death.^92,93^

#### Adaptive immune signaling pathways contribute immunosuppression and prevent autoimmunity

Tregs exert an immunosuppressive function through the production of anti-inflammatory cytokines like TGF-β and IL-10, playing a role in maintaining peripheral tolerance.^94^ However, they also limit sterilizing immunity against abnormal self, such as cancer cells and mutated cells.^95^ Usually, these transformed target cells could be eliminated by cytotoxic T lymphocytes (CTLs).^96^ In addition to the cell-mediated adaptive immune response, specific antibodies produced by B cells also play a role in maintaining the homeostasis of the cardiovascular system. For example, cardiovascular-reactive natural antibodies (NAbs) can be produced in the absence of infection, even under homeostatic conditions. NAbs interact with multiple self-derived antigens, providing benefits in autoimmunity prevention.^97^

## The endotypes and immuno-features Of CVDs

**Fig. 3:**
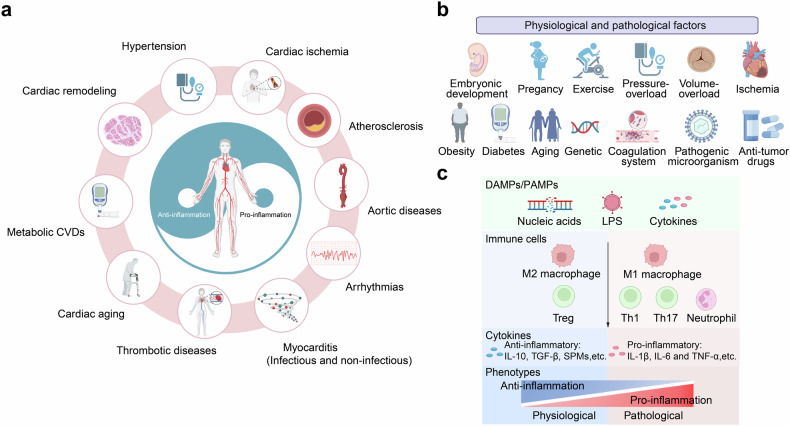
Mechanisms of Immune Regulation in Physiological and Pathological Processes of CVDs. **a** Immune regulation in various kinds of CVDs. **b** Physiological and pathological factors in CVDs. **c** Physiological and pathological immune regulation in CVDs. CVDs cardiovascular diseases, PAMPs Pathogen-Associated Molecular Patterns, DAMPs Damage-Associated Molecular Patterns, LPS Lipopolysaccharide, Tregs Regulatory T cells, Th1 cell T helper 1 cell, Th17 cell T helper 17 cell, IL-10 Interleukin-10, TGF-β Transforming Growth Factor-beta, SPMs Specialized Pro-resolving Mediators, IL-6 Interleukin-6, TNF-α Tumor Necrosis Factor-α(Created with BioRender.com, https://BioRender.com/r16i827)

CVDs encompass various endotypes such as hypertension, atherosclerosis, ischemic heart disease, cardiac remodeling, chronic heart failure, metabolic cardiomyopathy, diabetic cardiomyopathy, aortic disease, cardiac aging, arrhythmia, inflammatory and infectious cardiomyopathy, cardiotoxicity of antitumor drugs, and thrombotic disease. Traditionally, CVDs were understood through their pathophysiological aspects, like plaque buildup and heart muscle failure. However, recent advancements have revealed the immuno-features in the pathogenesis and progression of these diseases (Fig. 3).

### Hypertension and immuno-dysregulation

Hypertension is a global health challenge, impacting over 1.3 billion people worldwide, with an increasing prevalence among younger individuals.^98,99^ Emerging evidence suggests that the development of hypertension is closely linked to immune dysregulation.^100,101^ Genetic and integrative network analysis showed that some single-nucleotide polymorphisms (SNPs) or genes related to immune response have been implicated in hypertension.^102^ In individuals with immune-mediated diseases, the risk of developing hypertension increases by 22% to 90%.^103–106^

#### Immune regulation in normal blood pressure

Normal blood pressure regulation is based on a delicate balance between pro-inflammatory and anti-inflammatory responses. Tregs are vital for vascular homeostasis under physiological conditions, as they suppress excessive inflammation and oxidative stress by producing anti-inflammatory cytokines like IL-10 and TGF−β, thereby maintaining normal endothelial function and vascular tone.^107,108^ Also, resident macrophages in the vessel wall and perivascular adipose tissue contribute to blood pressure regulation by modulating vascular reactivity and NO production.^8^ DCs maintain a tolerogenic state under normal conditions, preventing over-activated immune response that could lead to hypertension.^109–111^ Moreover, renal DCs interact with tubular cells to maintain normal renal sodium handling and blood pressure by regulating local inflammation.^112^ Additionally, the immune system closely interacts with the nervous system to regulate blood pressure. Vagus nerve activation reduces excessive inflammation by releasing acetylcholine to lower pro-inflammatory cytokines production, while the sympathetic nervous system regulates immune cell function and trafficking, maintaining balanced blood pressure.^113,114^

#### Immune dysregulation in hypertension

Hypertension often results from chronic, dysregulated inflammation driven by various immune and non-immune cells, including T cells and endothelial cells (ECs).^115^ Its onset can be triggered by factors like renin-angiotensin-aldosterone system activation, the sympathetic nervous system (SNS) stimulation, high salt intake, stress, eicosanoid changes, mechanical forces, or proteasome inhibitors treatment.^109,116^ These pro-hypertensive stimuli drive the release of upstream inflammatory regulators, leading to local inflammation and mechanical and oxidative damage.^109,116,117^

T cells play a vital role in the development of hypertension. The increase in CD3 + CD45RO+ memory T cells, especially CD8+ effector memory T cells, which exhibit upregulation of pathways related to mitochondrial oxidative metabolism and inflammatory activation, is also associated with hypertension.^118,119^ Moreover, activated DCs produce IL-6, IL-23, and IL-1β, which drive T cell polarization and the production of effector cytokines.^109^ In addition to immune cells, non-immune cells such as endothelial cells can also affect the progression of hypertension.^109,120^ For example, IL-10 deficiency aggravates angiotensin II-induced endothelial dysfunction and superoxide production, which contribute to hypertension.^121^ This immune-endothelial crosstalk illustrates the complex interactions that drive hypertension.

### Atherosclerosis and immuno-dysregulation

Atherosclerosis, the primary underlying pathology of coronary artery disease (CAD), is characterized by the chronic accumulation or acute rupture of vessel-occluding plaques in the subendothelial intimal layer of large and medium-sized arteries.^122^ This process ultimately leads to significant stenosis, restricting blood flow and causing critical tissue hypoxia.

#### Immune regulation in vascular homeostasis

Under physiological conditions, the immune system plays a critical role in maintaining vascular health through balanced inflammatory and anti-inflammatory responses. ECs are central to this process, as they maintain vascular tone, support hemostasis, and regulate thrombosis.^123,124^ ECs respond to inflammatory signals by secreting mediators that initiate both innate and adaptive immune responses.^125^

In normal inflammation, immune cells like neutrophils and macrophages produce cytokines and chemokines to amplify the localized immune response. These molecules recruit additional immune cells, creating a balance between pro-inflammatory cytokines such as TNF-α, IL-6, and IL-1, and anti-inflammatory mediators like IL-10 and TGF-β, which are essential for vascular stability and function.^126^ Tregs further aid in this process by modulating inflammatory responses and preventing excessive vascular inflammation.^107,108^

#### Immune dysregulation in atherosclerosis development

The initiation of atherosclerosis is driven by immune dysregulation triggered by hemodynamic forces, particularly in regions of low shear stress. This hemodynamic environment contributes to endothelial dysfunction, allowing the infiltration of apolipoprotein B (ApoB)-containing lipoproteins into the subendothelial space.^127^ Upon activation, endothelial cells secrete chemokines that recruit monocytes, which differentiate into macrophages within the vascular wall. These macrophages, upon taking up lipoproteins, transform into lipid-laden foam cells, marking the onset of plaque formation.^127^

Additionally, antigen-presenting cells (APCs) such as macrophages and DCs present lipid and peptide antigens to invariant natural killer T (iNKT) cells and T cells. This interaction triggers adaptive immune responses that contribute to plaque progression.^128^ Single-cell transcriptomics have shown that VSMC-derived foam cells constitute a significant portion of foam cells, with these cells demonstrating phenotypic plasticity.^128^ They can adopt macrophage-like characteristics that exacerbate lesion growth or fibroblast-like traits that stabilize plaques.^129–131^ Collectively, these processes worsen endothelial dysfunction and drive additional inflammation through continued monocyte recruitment, increased lipoprotein uptake (which adds to the plaque’s lipid load), VSMC activation and proliferation, and fibroblast migration, which aids in forming the fibrous cap.

#### Immune crosstalk and plaque progression

The progression of atherosclerosis involves continuous immune cell recruitment and inflammation. Monocytes migrate to the subendothelial layer, where they differentiate into macrophages, perpetuating inflammatory responses through cytokine production and further lipoprotein uptake, which contributes to plaque lipid accumulation.^126^ VSMC activation and proliferation add to the plaque mass, while fibroblast migration contributes to the formation of a fibrous cap that stabilizes the plaque but can also increase the risk of rupture in vulnerable plaques.^126^

The crosstalk between immune cells in atherosclerosis highlights the intricate interactions at play, involving not only the innate immune system but also adaptive immune responses that drive disease progression. This cross-talk between immune cells and vascular structures reinforces inflammation, fostering an environment conducive to plaque buildup and instability, which underpins the pathology of atherosclerosis and its progression to CAD.

### Ischemia heart disease and immuno-dysregulation

Ischemia heart disease occurs when blood flow to the heart muscle is reduced, usually due to partial or complete blockage of coronary arteries. The most common cause is atherosclerosis—the buildup of plaque in the coronary arteries.^132^ Other causes can include coronary artery spasm, thrombosis, and coronary artery dissection.^133,134^ Ischemic heart disease has the highest global age-standardized DALY at 2275.9 per 100,000.^135^ It occurs when blood flow to the heart muscle is reduced, usually due to partial or complete blockage of coronary arteries.

#### Immune dysregulation in ischemia heart disease

The immune response plays a complex and stage-specific role in myocardial ischemia, encompassing both inflammatory and reparative processes. Within hours following ischemic injury, CD4 + T helper cells, particularly Th1 and Th17 subsets, are recruited to the myocardium,^136^ where they produce pro-inflammatory cytokines, such as IFN-γ and IL-17, which escalate inflammation and attract additional immune cells to the site.^137^ This early influx of pro-inflammatory cells establishes a highly reactive environment that can lead to exacerbated injury if unchecked. Tregs are also quickly activated during the early stages of the ischemic response, playing a protective role by modulating inflammation and promoting tissue repair. They secrete anti-inflammatory cytokines like IL-10 and TGF−β, which help to control excessive inflammation and support the resolution phase.^138^ This dual response highlights the immune system’s dynamic involvement, with both pro-inflammatory and anti-inflammatory pathways engaged in managing ischemic damage.

B lymphocytes are activated within the first 24-48 h post-ischemia, contributing to the early immune response through antigen presentation and production of auto-antibodies against cardiac antigens exposed during tissue damage.^139^ Some subsets of B cells, particularly regulatory B cells, may have a protective role by producing IL-10 and modulating T cell responses.^140^ Thus, modulating the activity of lymphocytes may offer promising approaches to mitigate ischemic injury and improve cardiac outcomes.

#### Crosstalk and therapeutic implications in immune response to ischemia

Crosstalk among immune cells in ischemic heart disease is critical, as it shapes the progression and resolution of inflammation in myocardial tissue. For instance, interactions between Tregs and Th17 cells modulate the intensity and duration of the inflammatory response, with an overactive Th17 response potentially leading to prolonged inflammation and myocardial damage, while Tregs help suppress excessive immune activation.^80,141^ This balance is essential for tissue recovery, and dysregulation at any stage can exacerbate ischemic injury or hinder repair mechanisms.

B cells also interact with T cells in the ischemic heart, influencing the overall immune response; while effector B cells promote inflammation through antigen presentation and antibody production, regulatory B cells help mitigate immune activation.^84,142^ Understanding these interactions provides insights into potential therapeutic approaches to limit ischemic injury, highlighting the value of targeting specific immune cell types or pathways to enhance cardiac recovery and prevent further ischemic damage.

### Cardiac remodeling and immuno-dysregulation

Cardiac remodeling involves structural and functional changes in the heart due to hemodynamic overload and/or cardiac injury.^143^ Changes in the heart’s size, shape, and function are clinically observed and detected through echocardiography, magnetic resonance imaging (MRI), positron emission tomography (PET) scans, ventriculography, and tomography.^144–146^ Remodeling can be either physiological or pathological, classified as adaptive or maladaptive.^143^ Physiological hypertrophy, occurring during development, pregnancy, or endurance training, is fully reversible.^147–149^ It features mild heart growth (10–20% larger than normal), no reactivation of fetal genes, increased cardiomyocyte growth in both length and width, angiogenesis, and lack of apoptosis and interstitial fibrosis.^143^ However, pathological remodeling occurs in acute and chronic phase of MI, pressure-overloaded conditions, volume-overloaded conditions, or genetic changes.

#### Immune regulation in physiological cardiac remodeling

The immune system plays a critical role in modulating physiological cardiac remodeling during both development and in adulthood.^150^ In normal conditions, cardiac-resident macrophages, derived from embryonic origins, predominate and are maintained through local proliferation. These macrophages support homeostasis by modulating local inflammation and promoting angiogenesis without triggering adverse remodeling.^55,151,152^ Additionally, circulating CCR2+ monocytes contribute minimally to the cardiac macrophage population under these conditions, highlighting the importance of resident macrophages in maintaining physiological homeostasis.^55,151^

Physical exercise has been shown to modulate macrophage function by promoting a shift from pro-inflammatory M1 macrophages to anti-inflammatory M2 macrophages. This transition helps enhance cardiac function and minimize interstitial fibrosis, suggesting that lifestyle interventions could modulate immune responses favorably.^153^ Exercise also activates cardiac-resident stem cells, contributing to cardiac repair and regeneration through immune modulation.^154^

#### Immune regulation in pathological cardiac remodeling

Pathological cardiac remodeling occurs following cardiac injury or sustained mechanical stress. This process involves significant changes in the immune system, with infiltrating monocytes and macrophages exacerbating adverse remodeling.^155^ In this context, mechanical stress activates innate immune responses, leading to the recruitment of neutrophils and macrophages to the myocardium. These cells release pro-inflammatory cytokines, such as TNF-α, IL-1β, and IL-6, which contribute to cardiomyocyte hypertrophy, fibrosis, and tissue damage.

The balance between M1 and M2 macrophages influences the progression of pathological remodeling, where an excess of M1 macrophages can drive fibrosis and hypertrophy, while M2 macrophages attempt to counteract these effects.^156^ Cardiac-resident macrophages exhibit a protective effect by regulating inflammation, whereas infiltrating monocyte-derived macrophages contribute to adverse outcomes. Additionally, T cells, particularly CD4 + T cells, infiltrate the myocardium, promoting fibrosis and inflammation, thereby contributing to ventricular stiffness and dysfunction.^142^

#### Immune crosstalk and heart failure development

The development of heart failure involves intricate interactions between innate and adaptive immune responses. Initially, cardiac injury triggers the activation of innate immune cells, including macrophages and neutrophils, which release pro-inflammatory cytokines such as TNF-α, IL-1β, and IL-6.^157^ These cytokines promote inflammation and cardiac remodeling by promoting hypertrophy and fibrosis.^157^ As heart failure progresses, chronic immune activation becomes prominent, marked by the involvement of adaptive immune cells, particularly T lymphocytes. CD4+ and CD8 + T cells expand systemically and infiltrate the failing myocardium.^158^ This persistent immune activation leads to a state of chronic low-grade inflammation that exacerbates cardiac dysfunction. Also, compensatory anti-inflammatory mechanisms are activated, including the production of IL-10 and TGF-β, in an attempt to resolve inflammation and promote tissue repair.^159^ However, the balance between pro- and anti-inflammatory processes eventually becomes dysregulated, leading to maladaptive ventricular remodeling and the progression of heart failure.^159^

The neurohumoral activation induced by mechanical stress also interacts with immune responses, enhancing inflammation and contributing to adverse remodeling.^160^ Over time, compensatory anti-inflammatory mechanisms, such as the production of IL-10 and TGF-β, attempt to resolve inflammation and promote tissue repair. However, this balance between pro- and anti-inflammatory factors becomes dysregulated in chronic heart failure, leading to further deterioration.

### Metabolic cardiomyopathy and immuno-dysregulation

Metabolic cardiomyopathy is a chronic metabolic disorder characterized by structural and functional cardiac changes, occurring independently of hypertension and coronary artery disease. It involves interstitial fibrosis, diastolic and systolic dysfunction, and cardiomyocyte injury. In its early stages, metabolic disturbances may not significantly affect myocardial structure or cardiac function, but they induce low-grade inflammation in the heart, leading to impaired myocardial relaxation due to abnormalities in subcellular components, such as endoplasmic reticulum stress, oxidative stress, calcium handing, and impaired mitochondrial dysfunction. In the advanced stage, a vicious cycle of subcellular component abnormalities and immune cell infiltration leads to cardiomyocyte injury, death, and cardiac fibrosis, ultimately impairing both diastolic and systolic functions.^161^

#### Immune regulation in myocardial metabolism

Recent studies have highlighted the impact of cellular metabolism on immune activation, with coordinated regulation benefiting the organism by optimizing energy resources during immune or inflammatory responses.^162^ Nutrient-sensing pathways can trigger immune responses, while inflammatory or stress responses inhibit anabolic pathways like insulin/insulin-like growth factor (IGF) signaling, diverting energy metabolism from synthesis to catabolism.^163^ A key concept in this context is “trained immunity,” which refers to the long-term functional reprogramming of innate immune cells, especially monocytes and macrophages, following metabolic stress or inflammatory stimuli. This adaptation can contribute to the sustained low-grade inflammation seen in cardiometabolic diseases.^164^

#### Immune dysregulation in metabolic cardiomyopathy

Emerging clinical evidence indicates strong links between the immune system and the development of metabolic cardiomyopathy.^161^ Macrophages play a crucial role in the development of metabolic cardiomyopathy.^8^ M1 macrophages secrete inflammatory cytokines that impair systemic and cardiac insulin signaling, and their presence is associated with metabolic cardiomyopathy induced by a Western diet in mice.^165^ Additionally, inflammation in β-cells leads to β-cell dysfunction, which combined with insulin resistance, exacerbates the condition.^166^

Beyond immune cells, endothelial cells and myofibroblasts in metabolic cardiomyopathy contribute to dysregulated immune responses.^167–169^ For instance, in human epicardial adipose tissue treated for diabetes, pro-inflammatory cytokines like TNF-α and IL-1β induce an inflammatory phenotype in human coronary endothelial cells, resulting in diminished vascular progenitor potential and promoting cardiomyopathy development.^170,171^ Furthermore, myofibroblasts activated by inflammatory mediators such as IL-13, IL-18, and MMPs play a significant role in initiating myocardial fibrosis. This process increases cardiac stiffness and impairs the heart’s contractile and relaxation functions.^172^ The interplay between immune cells and these non-immune cells perpetuates a cycle of inflammation and fibrosis, which ultimately leads to metabolic cardiomyopathy progression.

### Aortic diseases and immuno-dysregulation

Aortic diseases are a variety of conditions affecting the aorta, the main artery, including congenital or acquired diseases of the chest and abdomen. They can be divided into three categories, thoracic aortic aneurysm (TAA), abdominal aortic aneurysm (AAA) and acute aortic syndrome (AAS).^173^ These conditions can result in life-threatening complications, such as aortic dissection, which carries a mortality rate exceeding 80 percent.^174^

#### Immune regulation in the aorta under physiological conditions

In physiological states, the immune system plays a pivotal role in maintaining aortic homeostasis by preventing excessive inflammation and supporting vascular function.^175,176^

At the forefront of this regulation are Tregs, which secrete anti-inflammatory cytokines such as IL-10 and TGF-β.^177^ These cytokines are crucial in mitigating inflammation and oxidative stress, thereby preserving endothelial cell function and reducing vascular stiffness to maintain aortic integrity. Resident macrophages within the aortic wall significantly contribute to vascular stability by producing NO and anti-inflammatory cytokines.^8^ These macrophages regulate vascular tone and prevent inflammation, thereby protecting endothelial health.^8^ Additionally, endothelial cells are key regulators within this network, producing NO and modulating cytokine levels to establish an anti-inflammatory environment.^178^ This reduces immune cell adhesion to the aortic wall, thus preserving vascular integrity and preventing inflammation-induced injury.^178^ DCs are another critical element in maintaining immune tolerance within the aortic tissue.^179^ They help establish a regulatory environment that curtails excessive immune activation, thereby preventing potential inflammatory damage.

Mechanical forces, such as shear stress resulting from blood flow, also play an essential role in modulating immune responses within the aorta.^180^ Macrophages and other immune cells can detect these forces and subsequently adjust their cytokine production to reduce inflammation, enabling them to respond to physiological changes in blood flow.^180^ Furthermore, aortic smooth muscle cells contribute to immune regulation by interacting with macrophages and releasing anti-inflammatory mediators, thereby maintaining a stable aortic wall and preventing inflammatory cell infiltration.^181^ Moreover, the SNS interacts closely with immune cells in the aorta, modulating their trafficking and activation.^182^ This interaction is vital to preventing excessive immune infiltration and maintaining balanced vascular reactivity.^182^ This neural-immune interaction is essential for maintaining vascular health and controlling inflammation within the aorta. Collectively, these cellular and molecular mechanisms underscore the intricate yet essential role of immune regulation in preserving aortic homeostasis, providing valuable insights into how immune cells and signaling pathways work in concert to maintain a healthy vascular environment.

#### Immune dysregulation in aortic aneurysms

The underlying pathology of aortic aneurysms involves significant immune cell infiltration and activation within the aneurysmal regions. This immune response contributes to inflammation and progressive structural degradation of the aortic wall.^183^ Macrophages, DCs, and T lymphocytes play central roles in maintaining aortic homeostasis and modulating inflammatory responses within the aorta. Macrophages are particularly abundant in aneurysmal tissue, where they accumulate and undergo local expansion through self-renewal.^184^ This macrophage presence is critical to aneurysm progression; macrophage depletion has been shown to disrupt endothelial integrity, leading to fibrin accumulation and microthrombus formation.^185^ DCs within the normal aorta have high antigen-presenting capacity and are essential in maintaining immune homeostasis by capturing and presenting antigens effectively.^186,187^ In aneurysmal tissues, however, DCs contribute to inflammation through the recruitment and activation of T cells, exacerbating tissue remodeling.^188^

T lymphocytes play an instrumental role in regulating aortic tissue integrity by secreting cytokines and modulating apoptosis and extracellular matrix remodeling. Recent single-cell transcriptome analysis reveals that T lymphocytes are abundant in aneurysmal aortic tissue and exhibit clonal expansion, suggesting their active involvement in the pathogenesis of aortic aneurysms.^176,189^ These T cells can drive inflammation and matrix degradation, leading to compromised structural stability of the aortic wall, which increases the risk of dissection and rupture. In addition, Tregs may have a protective role in modulating excessive immune activation and tissue destruction.^190^ Tregs can counterbalance pro-inflammatory responses by secreting anti-inflammatory cytokines, thereby promoting tissue stability and potentially limiting aneurysm expansion. However, dysregulation in Treg activity or quantity may contribute to unchecked inflammation and accelerated aneurysm development.

### Cardiac aging and immuno-dysregulation

Cardiac aging is a gradual process that diminishes cardiac structure and function due to the cumulative impact of internal and external stressors. With aging, the myocardium undergoes structural changes, including increased cardiomyocyte hypertrophy, interstitial fibrosis, and chronic inflammation, ultimately contributing to diastolic and systolic dysfunction.^191^

#### Immune dysregulation in cardiac aging

At the cellular level, various forms of cellular senescence contribute to cardiac aging. Immune cells, including T cells, mast cells, and macrophages, regulate tissue homeostasis and pathogenesis by modulating inflammatory responses and myocardial senescence in cardiac tissue.^191^ T cells can affect age-related diseases, including senescence, through several mechanisms, as outlined below: 1) Age-associated T cells continue to produce cytokines, such as IFN-γ and TNF-α, which leads to chronic inflammation and promotes senescence of neighboring cells^192^; 2) T cells enhance senescence-associated secretory phenotypes (SASP), which further exacerbate inflammation and Th17/Th1 cell differentiation, leading to tissue damage^193^; 3) Dysfunctional T cells fail to clear senescent cells, leading to the accumulation of these cells and exacerbating tissue damage^194^; Senescent CD8+ and CD4 + T cells acquire cytotoxicity, which directly damages tissue cells.^195,196^

Mast cells and macrophages also contribute to cardiac aging by promoting cardiomyocyte hypertrophy and cellular senescence. Mast cells release enzymes like chymotrypsin, which influence hypertrophy, while macrophages drive senescence through the activity of connexins and pro-inflammatory cytokines.^197,198^ Additionally, macrophages in the aging heart release cytokines, such as IL-6, TNF-α, and IL-1, which have been shown to induce an osteogenic phenotype in valvular interstitial cells (VICs).^199^ This change promotes calcification and fibrosis within heart valves, impairing valve function and exacerbating age-related cardiac dysfunction.^199^

#### Involvement of non-immune cells in cardiac aging

Non-immune cells, such as endothelial cells, VSMCs, and VICs, also play crucial roles in cardiac aging. Senescent endothelial cells release inflammatory chemokines and cytokines, with reduced levels of anti-inflammatory molecules, contributing to a pro-inflammatory environment.^200^ VSMCs display a SASP, characterized by the secretion of monocyte chemotactic protein-1 (MCP-1), chemokine (C-C motif) ligand 3/4 (CCL3/4), and various interleukins (IL-1, IL-6, IL-8), further promoting inflammation and fibrosis.^201^ VICs, as the primary cell type in heart valves, also undergo senescence, impairing their function and contributing to calcification and fibrosis. Pro-inflammatory cytokines released by macrophages in aging cardiac tissue further promote VIC senescence and functional decline.^191^

### Arrhythmia and immuno-dysregulation

Arrhythmias, including atrial fibrillation (AF) and ventricular arrhythmias, arise from disruptions in cardiac electrical activity and conduction, which are influenced by the immune system.^202^ Inflammatory cells, especially macrophages, are pivotal in maintaining cardiac electrical stability and have direct and indirect roles in modulating cardiac conduction.^65^ These roles include influencing ion channel expression and promoting fibrotic changes that alter the electrical landscape of the myocardium.^65^

#### Immune regulation in cardiac conduction

Macrophages in cardiac tissue interact with cardiomyocytes and influence electrical conduction through the modulation of ion channels. They express conduction-related genes, including those encoding ion channels such as Cacna1c (Cav1.2), Kcnj2 (Kir2.1), Kcnq1 (Kv7.1), Hcn2 (HCN2), and Kcnh2 (Kv11.1).^65^ Additionally, macrophages interact with cardiomyocytes through gap junctions formed by connexin 43 (Cx43), impacting both resting and action potentials in cardiomyocytes.^65^ Through these mechanisms, macrophages contribute to arrhythmogenesis by altering the electrophysiological properties of the heart.

#### Immune regulation in arrhythmia

A strong link exists between inflammation and arrhythmias,^202^ including ventricular tachyarrhythmias due to myocarditis.^203^New-onset AF is common in acute sepsis.^204^ Existing studies suggest that inflammatory signaling in cardiomyocytes has a key role in the development of AF, and in particular, NLR family pyrin domain containing 3 (NLRP3) inflammatory vesicles are particularly associated with cardiomyocyte-mediated inflammatory signaling in AF.^205^ Autoantibodies contribute to the development of arrhythmias by modulating the function of cardiac ion channels and significantly affecting cardiac electrical activity.^206^ Bradyarrhythmias and conduction disorders: anti-Ro/SSA antibodies target L-type and T-type calcium channels, inhibit calcium currents, and affect sodium currents in the sinoatrial node (SA node) and atrioventricular node (AV node).^207,208^

Autoantibodies have also been implicated in arrhythmia development by targeting cardiac ion channels, thereby altering cardiac electrical activity. For example, in bradyarrhythmias, anti-Ro/SSA antibodies target L-type and T-type calcium channels, inhibiting calcium currents and impacting the SA and AV nodes.^209^ In conditions such as Long QT syndrome (LQTS), anti-SSA antibodies targeting K11.1V11.1 K channels (hERG) inhibit potassium currents, resulting in delayed repolarization.^210^ Autoantibodies targeting K1.4V1.4 K channels may inhibit transient outward potassium currents.^211–213^ Conversely, in Short QT syndrome (SQTS), autoantibodies targeting K7.1V7.1 potassium channels increase potassium currents, accelerating repolarization and predisposing the heart to arrhythmic episodes.^214^

### Myocarditis and immuno-dysregulation

Myocarditis is characterized by the infiltration of inflammatory cells into the myocardium, which increases the risk of cardiac dysfunction. It can be caused by a wide range of factors, classified into infectious and non-infectious types.^215^ The immune system’s role in myocarditis is complex, as immune regulation is crucial for both protecting cardiomyocytes from pathogens and managing inflammation to prevent further tissue damage.

#### Immune dysregulation in infectious myocarditis

Infectious myocarditis, commonly caused by viral infections, may also result from bacterial, protozoal, or fungal infections.^216^ Immunoregulatory mechanisms play a vital role in the development and progression of cardiomyopathies in both physiological and pathological states. Under normal conditions, the heart maintains a balanced immune state to protect cardiomyocytes from pathogens while avoiding tissue damage from excessive immune response.^217^ Following infection, immune cells in the myocardium initiate an inflammatory response upon recognizing pathogens, which is vital for controlling infections but can also damage myocardial tissue. For instance, tripartite motif-containing protein 18 (TRIM18) regulates viral myocarditis by modulating TBK1-mediated immune responses in macrophages, thereby limiting the extent of inflammation.^218^ Additionally, TRIM29 has been shown to control viral myocarditis through the regulation of ER stress and ROS responses in macrophages.^219^Moreover, the heart-spleen axis is essential in managing the systemic inflammatory response; by preventing the recruitment of pro-inflammatory monocytes to the myocardium, this axis helps mitigate myocardial damage and chronic inflammation, emphasizing the importance of balanced immune signaling in limiting disease progression.^220^

#### Immune dysregulation in non-infectious myocarditis

Non-infectious myocarditis is often associated with immune-modulatory treatments, particularly immune checkpoint inhibitors (ICIs) and CAR T-cell therapy. In ICIs-induced myocarditis, CCR2+ macrophages are significantly recruited to the heart, creating a pro-inflammatory environment that accelerates myocardial damage.^221^ T cells also contribute to this damage through clonal expansion and recognition of myocardial antigens, which exacerbates inflammation and can lead to further myocardial injury.^222,223^ Additionally, “epitope spreading” in this context may lead to tumor-specific T cells attacking cardiac tissue, expanding the scope of immune dysregulation.^223^

In CAR T cell therapy, myocarditis can arise through multiple mechanisms: 1) Cytokine Release Syndrome (CRS): The anti-tumor activity of CAR T cells often triggers CRS, a systemic inflammatory response associated with high circulating cytokine levels, which correlates with the severity of adverse cardiac events.^224–226^ 2) Cross-Reactivity with Myocardial Antigens: CAR T cells may inadvertently target myocardial proteins, as seen with melanoma-associated antigen-3 (MAGE-3), which cross-reacts with titin, a myocardial protein, leading to fulminant myocarditis^227^; 3) Off-Target Effects: Immune responses directed at non-tumor antigens unrelated to the intended targets of CAR T therapy can also result in cardiac injury, underscoring the broad impact of immune dysregulation on myocardial health.^224,225^

### Cardiotoxicity and immuno-dysregulation

Cardiotoxicity, a severe adverse effect of numerous drugs, particularly those used in cancer chemotherapy and anti-viral treatments, poses significant challenges in clinical applications and patient management. The immune system plays a crucial role in the cardiotoxicity caused by various anti-tumor and anti-viral drugs.

Many anti-tumor drugs, such as anthracyclines (doxorubicin and pirarubicin), triptolide, antibody-Drug Conjugates (ADCs) are widely associated with cardiotoxicity. Anthracyclines (doxorubicin and pirarubicin) are widely associated with cardiotoxicity due to its oxidative stress induction and DNA damage in cardiomyocytes.^228^ Studies indicate that doxorubicin activates an immune response, recruiting inflammatory cells to the heart and initiating pro-inflammatory cytokine release.^229^ This inflammation often leads to fibrosis and eventual heart failure. The presence of T cells, specifically CD8+ cytotoxic T cells, exacerbates this cardiac damage by promoting fibrosis and systolic dysfunction.^230^ Triptolide, derived from the herb *Tripterygium wilfordii*, is a highly potent anti-tumor agent but is limited by its cardiotoxicity.^231,232^ Triptolide induces mitochondrial damage in cardiac cells, leading to dysfunction in energy production and increased oxidative stress. It also triggers an immune response, with macrophages playing a key role in the resultant inflammation.^231^ ADCs are engineered to target specific tumor cells, but their toxic payloads can lead to unintended cardiac toxicity.^233^ ADCs may cause off-target effects, where immune cells, particularly macrophages, respond to the cytotoxic payload released in cardiac tissue.^234^ This immune activation leads to the release of pro-inflammatory cytokines such as TNF−α and IL-6, which promote inflammation, oxidative stress, and endothelial damage, thereby exacerbating cardiac injury. Some ADCs can trigger delayed hypersensitivity reactions, where immune cells initiate a T-cell-mediated response.^235^ This response involves the release of cytotoxic mediators that target cardiac cells, leading to inflammation and subsequent myocardial fibrosis and heart failure.

Anti-viral treatments are associated with an increased risk of cardiotoxicity, which may involve immune activation. For example, abacavir induces pro-inflammatory cytokine release, promoting endothelial dysfunction and atherosclerosis, which increases cardiovascular risk.^236^ Ritonavir acts as a CYP3A inhibitor, affecting the metabolism of various cardiac medications and leading to immune cell activation and oxidative stress.^237,238^ This immune reaction can increase pro-inflammatory cytokines, causing myocardial strain, especially in critical COVID-19 cases where the immune response is already heightened.

### Thrombotic diseases and immuno-dysregulation

Thrombosis is the localized formation of blood clots that can affect arterial or venous circulation, potentially leading to severe conditions like myocardial infarction, pulmonary embolism, and thrombotic microangiopathy.^239^

#### Immune regulation in the blood coagulation system under physiological conditions

Under physiological conditions, immune regulation within the blood coagulation system is crucial for maintaining balance between coagulation and immune defense, ensuring that clot formation and inflammation are appropriately controlled.^199^ The complement system, for example, directly influences coagulation through its interaction with fibrin clots, which can activate pathways that prevent excessive inflammation and regulate clot formation.^199^ Factor H, a key regulator in this process, mitigates immune activation by binding to fibrin clots, thus ensuring a controlled immune response while supporting hemostasis.^240^

Interactions between the immune system and coagulation cascade are central to cardiovascular health.^241,242^ Pro-inflammatory cytokines, often released during immune responses, can trigger coagulation factors, which in turn help regulate inflammation and prevent infections.^243^ Immune cells like neutrophils and monocytes participate actively in the coagulation cascade by releasing factors that promote clotting when an immune response is necessary, thereby protecting tissue integrity and limiting pathogen spread.^242^ However, their activity is tightly regulated to avoid excessive clot formation, which could otherwise lead to thrombosis.^242^

Additionally, the immune-coagulation interplay is critical for managing immune responses in aging population.^244,245^ With age, immune cells may exhibit changes that impact coagulation, as evidenced by single-cell analyses showing alterations in immune and hematopoietic cell function related to immune aging.^244,245^ This balance is particularly crucial in the aging population, where dysregulated coagulation can contribute to age-related diseases.

These findings underscore the complex, tightly regulated relationship between the immune and coagulation systems under physiological conditions, where immune and metabolic signals finely tune clot formation and inflammation to maintain overall homeostasis and vascular health.

#### Immune dysregulation in thrombotic diseases

The rupture of an atherosclerotic plaque exposes the subendothelial matrix and releases tissue factor (TF), activating the coagulation cascade and promoting leukocyte recruitment via platelet adhesion and activation.^246–248^ Platelets, in particular, play a pivotal role by releasing chemokines and cytokines, including CCL5 and chemokine (C-X-C motif) ligand 4 (CXCL4), which recruit bone marrow-derived progenitor cells and leukocytes to the plaque site.^249–252^ These cells aid in vascular repair and mediate inflammatory responses that can stabilize or destabilize the plaque, influencing thrombus formation.

In acute conditions, such as COVID-19, immune dysregulation is evident in the form of neutrophil extracellular traps (NETs). Neutrophils interacting with platelets laden with pathogens release NETs that entrap pathogens and promote thrombosis.^253,254^ This process is intricately regulated by Nicotinamide Adenine Dinucleotide Phosphate (NADPH) oxidase and protein-arginine deiminase type 4 (PAD4), which are essential for modulating the immune response and preventing excessive thrombus formation.^255,256^ NETs play a dual role in immunity and thrombosis by targeting pathogens while inadvertently promoting clot formation, which can lead to microvascular obstruction and tissue ischemia in severe infections.

Platelets act as critical mediators of the immune response in thrombotic diseases. They release high-mobility group box 1 (HMGB1), which binds to receptors such as receptor for advanced glycation endproducts (RAGE) and Toll-like receptors (TLR2) on monocytes.^257^ This interaction triggers NET release from neutrophils and amplifies the inflammatory and coagulation cascade, further intensifying thrombus formation. Additionally, monocytes release TF, which activates both the extrinsic and intrinsic coagulation pathways, reinforcing clot formation and sustaining the cycle of inflammation and coagulation.^241^

## The Immune Signaling Pathways in Cvds

### Innate immune response

**Fig. 4:**
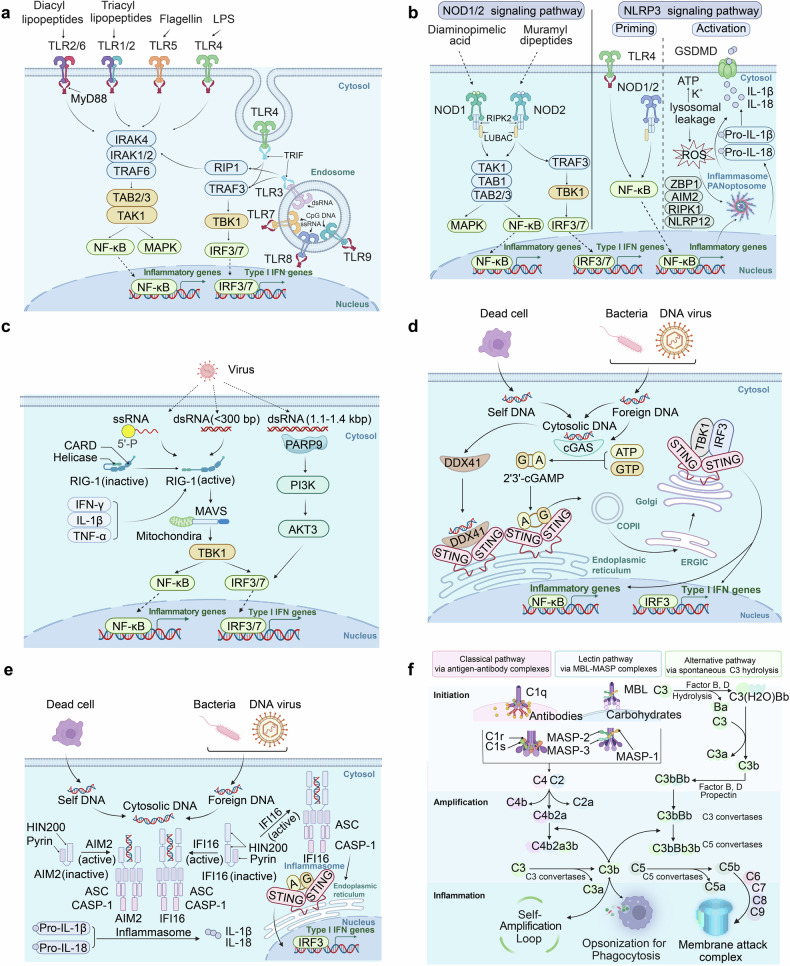
Innate Immune Signaling pathway in CVDs. **a** TLRs-Dependent Innate Immune Signaling pathway; **b** NLRs-dependent innate immune signaling pathway; **c** cGAS-STING signaling pathway; **d** ALRs-dependent innate immune signaling; **e** RLRs-dependent innate immune signaling; **f** The complement system-dependent pathways. TLR Toll-like receptor, LPS Lipopolysaccharide, IRAK Interleukin-1 receptor-associated kinase, TRAF6 TNF receptor-associated factor 6, TAB2/3 TGF-beta activated kinase 1/MAP3K7 binding protein 2/3, TAK1 Transforming growth factor beta-activated kinase 1, MAPK Mitogen-activated protein kinase, NF-κB Nuclear factor kappa-light-chain-enhancer of activated B cells, RIP1 Receptor-interacting serine/threonine-protein kinase 1, TRAF3 TNF receptor-associated factor 3, TBK1 TANK-binding kinase 1, IRF3/7 Interferon regulatory factor 3/7, NOD1/2 Nucleotide-binding oligomerization domain-containing protein 1/2, NLRP3 NLR family pyrin domain containing 3, GSDMD Gasdermin D, RIPK2 Receptor-interacting serine/threonine-protein kinase 2, LUBAC Linear ubiquitin chain assembly complex, ATP Adenosine triphosphate, ZBP1 Z-DNA binding protein 1, AIM2 Absent in melanoma 2, NLRP12 NLR family pyrin domain containing 12, IFN Interferon, cGAS Cyclic GMP-AMP synthase, STING Stimulator of interferon genes, GTP Guanosine triphosphate, 2'3’-cGAMP 2'3’-cyclic GMP-AMP, DDX41 DEAD-box helicase 41, HIN200 hematopoietic interferon-inducible nuclear proteins with a 200-amino-acid repeat, ASC Apoptosis-associated speck-like protein containing a CARD, CASP-1 Caspase-1, IFI16 Interferon-gamma inducible protein 16, MAVS Mitochondrial Antiviral Signaling Protein, pARP9 Poly(ADP-Ribose) Polymerase 9, AKT3 AKTserine/threonine kinase 3, IL Interleukin, TNF-α Tumor necrosis factor-α, RIG Retinoic acid-inducible gene, MASP Mannose-binding lectin-associated serine protease (Created with BioRender.com, https://BioRender.com/e69d507)

The innate immune system matters in CVDs.^4^ Pattern recognition receptors (PRRs), such as TLRs, are key components of the innate immune response that recognize damage-associated molecular patterns in cardiovascular tissues and initiate inflammatory cascades.^258^ Innate immune cells like macrophages and neutrophils contribute to both the progression and resolution of inflammation in CVDs, highlighting their dual role in tissue damage and repair.^259^ Sustained activation of innate immune signaling can lead to maladaptive inflammatory responses that promote cardiovascular dysfunction^4^ (Fig. 4).

#### TLRs-dependent innate immune response

##### TLRs

TLRs were the first family of PRRs discovered in the innate immune system.^260^ Ten TLRs (TLR1-10) have been identified in humans, each responsible for recognizing specific pathogen-associated molecular patterns (PAMPs) and damage-associated molecular patterns (DAMPs).^261^They are single-spanning receptors anchored in membrane structures, such as cell membranes, endosomes, and lysosomes. Leucine-rich repeats (LRRs), a conserved structural element in the extracellular region of TLRs, form the horseshoe-shaped ligand-binding domain responsible for binding a variety of PAMPs or DAMPs, such as lipopolysaccharides (LPS), peptidoglycans, flagellin, nucleic acids and oxidized low-density lipoprotein (ox-LDL).^262,263^ TLRs can be further categorized based on their cellular localization. For instance, TLR1, TLR2, TLR5, TLR6 and TLR10, are located on the plasma membrane to recognize extracellular pathogens. In contrast, endosomal TLRs such as TLR3, TLR7, TLR8, and TLR9 recognize nucleic acids from bacteria or viruses during endosomal or lysosomal degradation.^4^ TLR4 is unique in its localization, initially residing on the plasma membrane and later translocating to the endosomal membrane following endocytosis^264^(Fig. 4a).

##### TLRs signaling pathways

Ligand binding initiates dimerization of TLR ectodomains, which in turn causes dimerization of the intracellular Toll/Interleukin-1 receptor (TIR) domains of each TLR, activating downstream pathways and triggering inflammatory responses. Generally, TLR signaling pathways primarily rely on two key protein adapters: myeloid differentiation factor 88 (MyD88) and TIR domain-containing adapter-inducing IFN-β factor (TRIF). These adapters are recruited to the cytoplasmic TIR domain of TLRs to initiate downstream signaling cascades.

MyD88 is utilized by all plasma membrane TLRs and most endosomal TLRs, with the exception of TLR3. Upon dimerization of TIR domains, MyD88 binds to these domains and recruits IL-1 receptor-associated kinases (IRAK), including IRAK4, IRAK1, IRAK2, and IRAK-M, to form a protein complex known as the myddosome.^265,266^ Subsequently, IRAK1 undergoes autophosphorylation and then phosphorylates tumor necrosis factor receptor–associated factor 6 (TRAF6), which serves as a scaffold for other components.^267,268^ The adapter proteins TAK1-binding proteins 2 and 3 (TAB2 and TAB3) bring transforming growth factor-β-activated kinase 1 (TAK1) into proximity with IRAK1, activating TAK1 through close proximity-dependent transphosphorylation.^269^ Eventually, phosphorylated TAK1 activates the nuclear factor—κB (NF-κB) and mitogen-activated protein kinases (MAPKs) pathways.

TRIF is specifically recruited to TLR3 and TLR4 when these receptors localize to endosomes. TRIF binds to and activates TRAF3, forming a complex known as the triffosome.^270^ TRAF3 activates the TBK1 and is an inhibitor of NF-κB kinase (IKKi) along with NF-κB essential modulator (NEMO). Subsequently, TBK1 phosphorylates and activates IFN regulatory factor 3 and 7 (IRF3/7). Phosphorylation and dimerization of IRF3 and IRF7 facilitate their translocation into the nucleus, where they drive IFN production and subsequent IFN-stimulated genes (ISGs) expression^271–273^(Fig. 4a).

##### Role of TLRs signaling pathways in CVDs

TLR signaling pathways play crucial roles in cardiac ischemia. In myocardial ischemia (MI), endogenous DAMPs, such as heat shock proteins, HMGB1, and nucleic acids, are released from damaged myocardial cells. TLRs are activated upon binding to these DAMPs, promoting the expression of inflammatory cytokines. Of note, the mRNA levels of TLR2, 3, 4 are approximately 10-fold higher than that of TLR1, 5 - 10.^274^ The mRNA and protein levels of TLR4 were elevated in the infarct and remote area post-MI compared to sham mice.^275^ TLR4 deficiency resulted in smaller infarct areas and less inflammation in mice subjected to myocardial IRI compared with wild-type (WT) mice.^276^ Similarly, inhibition of TLR4 with eritoran significantly reduced MI/R injury and mitigated inflammatory responses.^277^ Aside from TLR4, mice with TLR2 gene knockout (KO) exhibited less myocardial fibrosis and a higher survival rate, despite having infarct sizes and inflammation levels comparable to WT mice.^278^ Also, increased expression and signaling by TLR2 and TLR4 could be observed in the hearts of patients with advanced heart failure, contributing to the sustained activation of innate immunity in the failing hearts.^279^ TLR2 or TLR4 induced cardiac hypertrophy and fibrosis in mice by regulating immune microenvironment.^280,281^ Additionally, activation of TLR7/8 leads to autoimmune vasculopathy and results in severe pulmonary arterial hypertension.^282^

Several factors act as pro-inflammatory stimuli in the vascular system, with ox-LDL being identified as one of the most potent DAMPs driving atherogenesis.^263^ Ox-LDL particles can be recognized by TLR ligands, inducing lipid-laden macrophages to release inflammatory cytokines.^283^ TLR2 and TLR4 are particularly important in vascular inflammatory responses due to their high abundance in atheromatous plaques.^284^ Loss-of-function studies have demonstrated the significant role of TLRs in the pathogenesis of atherosclerosis. Deficiency of TLR4 in macrophages protects them from transforming into foam cells, thereby mitigating the severity of atherosclerosis.^285^ In addition to affecting innate immune cells, lipid accumulation can induce non-immune cells to adopt a maladaptive phenotype in the vascular wall. Ox-LDL upregulates TLR2 and TLR4 expression in endothelial cells, concomitant with increased levels of adhesion molecules like VCAM-1, ICAM-1, and MCP-1.^285^ A recent study also demonstrated that a TLR2 agonist significantly promotes chondrogenic differentiation of VSMCs, an initial step towards arterial calcification.^286^ Overall, the role of TLRs signaling pathway has been well characterized and widely implicated in CVDs.

#### Nucleotide oligomerization domain (NOD)-like receptors (NLRs)-dependent innate immune response

##### NLRs

NLRs, a large family of cytosolic sensors, activate innate immune and inflammatory responses by recognizing intracellular PAMPs and DAMPs. Specific domains largely determine the distinct functions of NLR family proteins. Mammalian NLRs share a similar architecture, categorized into three core domains: (1) an N-terminal variable domain for initiating downstream signaling; (2) a central nucleotide-binding domain (NBD) for oligomerization; and (3) a C-terminal horseshoe-shaped leucine-rich repeat (LRR) domain.^287^ Mammalian NLRs can be divided into four major subfamilies based on their different N-terminal domain structures: acidic transactivating domain-containing NLR (NLRA), baculovirus inhibitor of apoptosis protein repeat-containing NLR (NLRB), caspase activation and recruitment domain (CARD)-containing NLR (NLRC), and pyrin domain-containing NLR (NLRP).

##### NLR signaling pathways

NLR family members are crucial in regulating various innate immune pathways, including NF-κB signaling, and cytokine and chemokine production. The functions of NLRs are diverse. Some modulate MHC class I or II genes and even Th2 response, while others form multi-protein complexes like inflammasomes or PANoptosomes.^288–291^ These complexes trigger caspase cleavage, leading to the maturation of IL-1β and IL-18 and subsequent cell death. For instance, nucleotide-binding and oligomerization domain-like receptors 1 and 2 (NOD1 and NOD2) are prominent in NLR-mediated inflammation in CVDs.^292^ NLRP3 is the most extensively studied NLR, recognized for its role in inflammasome or PANoptosome formation (Fig. 4b).

##### NOD1 and NOD2-dependent pathway

NOD1 and NOD2 are cytosolic sensors of bacterial peptidoglycans, essential for host defense and inflammation. Specifically, diaminopimelic acid binds to NOD1, and muramyl dipeptides bind to NOD2.^293,294^ NOD1 and NOD2 are associated with endosomal membranes, where they bind bacterial breakdown products transported through those membranes. Under steady-state conditions, NOD1 or NOD2 exists as an inactive monomer in the cytosol. Upon recognizing their specific ligands via the LRR regions, NOD1 and NOD2 self-oligomerize, undergoing a conformational change to recruit receptor-interacting serine/threonine kinase 2 (RIPK2) through homotypic CARD-CARD interactions.^295^ Subsequently, RIPK2 serves as a scaffolding protein that provides an organizing center for downstream signaling proteins.^296,297^ Further, the linear ubiquitin assembly complex (LUBAC) is recruited, mediating the recruitment of transforming growth factor β-activated kinase 1 (TAK1) and TAB1, TAB2 or TAB3, which forms a multi-protein complex termed as nodosome.^298^ Finally, these events contribute to the activation of MAPK pathways, NF-κB signaling and even IL-13 effector response.^299,300^ Alternatively, studies have shown that NOD1 binding to its ligand activates the serine-threonine kinase RICK and the TRAF3 complex, resulting in the phosphorylation of IRF3 and IRF7, which induces expression of type-1 IFN genes^301^ (Fig. 4b).

##### NLRP3-dependent inflammasomes and PANoptosomes

NLRP3 has recently garnered considerable attention due to its critical role in assembling inflammasomes. Mechanistically, the activation of NLRP3 inflammasome requires a 2-step process: priming (step 1) and protein complex assembly (step 2). Studies have shown that upregulation of NLRP3, pro-caspase-1, pro-IL-1β, and pro-IL-18 mRNA level via NF-κB pathway, mediated by TLR4 and NOD1/2, primes the activation of NLRP3-dependent inflammasome.^302,303^ During priming, inflammasome formation can be fine-tuned by various posttranslational modifications of NLRP3, including phosphorylation, deubiquitination, and sumoylation.^304–306^ The subsequent activation process involves NLRP3 oligomerization via homotypic NACHT-NACHT interactions, leading to the binding of apoptosis-associated speck-like protein containing a CARD (ASC) to NLRP3 and the recruitment of pro-caspase-1.^307^ During this step, signals of cellular instability and damage - such as potassium ion efflux, adenosine triphosphate release, and/or leakage of lysosomal contents - are proposed to induce NLRP3 inflammasome activation.^308^ Recent studies suggest that these cellular indicators may function via ROS, which are crucial for the interaction between NIMA-related kinase (NEK7) and NLRP3, thereby inducing inflammasome formation and activation.^309,310^ Inflammasomes activate caspase-1, which cleaves IL-1β and IL-18 precursors into their mature forms, thereby triggering and amplifying inflammatory responses that contribute to diseases such as chronic rhinosinusitis,^289^ very-early-onset inflammatory bowel disease,^311^ bronchiectasisand and non-T2 asthma.^312,313^ In addition, NLRP3, functioning as a key component of the PANoptosome - a complex involved in pyroptosis, apoptosis, and necroptosis - interacts with various NLRs and non-NLR sensors to form multi-protein complexes essential for innate immune responses^314–316^ (Fig. 4b).

### Role of NLRs signaling pathways in CVDs

#### Role of NOD1 and NOD2 signaling pathways in CVDs

The NOD1 and NOD2 signaling pathways have been associated with myocardial infarction, heart failure, and diabetic cardiomyopathy. Yang et al. first demonstrated that activating NOD1 with DAP (a synthetic activator) significantly worsened cardiac I/R injury and induced cardiomyocyte apoptosis in mice.^317^ Specifically, NOD1 activation induced myocardial fibrosis in diabetic mouse hearts.^318^ Similarly, Shen et al. reported NOD2 upregulation in diabetic cardiomyopathy in a mouse model of diabetes, and inhibiting NOD2 improved diabetes-induced myocardial fibrosis and cell apoptosis.^319^ However, NOD2 deficiency exacerbated cardiac hypertrophy and fibrosis in mice with pressure-overload-induced heart failure, indicating a unique role of NOD2 in various CVDs.^320^ Except for myocardial infarction, Kanno et al. reported that oral administration of a NOD1 ligand accelerated the progression of atherosclerosis in *ApoE*-/- mice by inducing vascular inflammation, whereas reduced development of atherosclerotic lesions was observed in *ApoE* and *Nod1* double-knockout mice.^321^ Similarly, the NOD2 cognate ligand increased lesion burden and vascular inflammation in atherotic cores in *Ldlr*-/- mice.^322^ Conversely, *ApoE* and *Nod2* double KO mice showed significant elevation in pro-inflammatory cytokines and atherosclerotic lesions.^323^ To investigate NOD2-mediated innate immune signaling in atherosclerosis, Liu et al. performed liquid chromatography coupled with tandem mass spectrometry to study the eicosanoid profiles after NOD2 activation. They discovered that NOD2 preferentially upregulated the prostaglandin E2 (PGE2) pathway. The role of PGE2 in atherosclerosis is complex, as it exhibits both pro-inflammatory and anti-inflammatory effects depending on cell types and PGE2 receptor subtypes, indicating a nuanced role for NOD2 in atherosclerosis.^324^ Intriguingly, a novel crosstalk between TLR4- and NOD2-mediated signaling was uncovered; NOD2 can sense the intensity of TLR4 signaling and modulate NF-κB pathway activation.^325^ This finding suggests that NOD2 serves as an immune initiator and functions as an immune regulator.^326^

#### Role of NLRP3-dependent inflammasomes and PANoptosomes in CVDs

NLRP3 is a prominent research topic in CVDs due to its involvement in myocardial infarction, cardiac hypertrophy, and atherosclerosis. In the ischemic heart of the mouse model, heightened inflammasome activation is evident from increased NLRP3 expression, elevated caspase-1 activity, and higher levels of IL-1β and IL-18.^327^ Inhibition of NRLP3-dependent inflammasome reduced cardiac inflammation and MI/R injury in mouse models.^327^ Similarly, treatment with the selective NLRP3-inflammasome inhibitor, MCC950, reduced infarct size and improved cardiac function in a pig model of myocardial infarction.^328^ While ischemia-induced NLRP3 inflammasome activation primarily occurs in immune cells,^329^ it has also been observed in non-immune heart cells like fibroblasts and cardiomyocytes. In vivo study, NLRP3 inflammasome could be activated after hypoxia/reoxygenation in cardiac fibroblasts.^330^ Moreover, MCC950-mediated NLRP3 suppression attenuated Ang II-induced hypertrophy and pyroptosis in neonatal mouse ventricular myocytes.^331^ Additionally, PANoptosis plays a role in the progression of various cardiovascular diseases, including heart failure,^332^ and NLRP3-dependent PANoptosis exacerbates doxycycline-induced cardiotoxicity in cardiomyocyte^333^ Of note, pro-atherogenic DAMPs, including oxidized low-density lipoprotein, free fatty acids, and cholesterol crystals, are potent triggers for NLRP3 inflammasome activation in macrophages, vascular smooth muscle cells or endothelial cells, driving the progression of atherosclerosis.^334–338^

### RNA sensor-dependent innate immune signaling

#### RNA sensors

##### Retinoic acid-inducible gene I (RIG-I)-like receptors (RLRs)

The RLRs are RNA helicases, RIG-I, melanoma differentiation-associated factor 5 (MDA5), and laboratory of genetics and physiology 2 (LGP2) - that function as cytoplasmic sensors of PAMPs.^261^ They recognize viral RNA and are involved in initiating and regulating innate immune response. RIG-I and MDA5 share similar structural features: an N-terminal region with tandem CARDs, a central DExD/H box RNA helicase domain capable of RNA binding, and a C-terminal repressor domain (RD) within the C-terminal domain (CTD) that autoregulates RIG-I.^339,340^ However, LGP2, a homolog of RIG-I and MDA5, competes with them for viral RNA binding, thereby inhibiting downstream signaling activation^261,341^(Fig. 4c).

##### Poly (ADP-ribose) polymerase family member 19 (PARP9)

Besides the canonical RNA sensors known as RLRs, PARP9 is an inactive mono-ADP-ribosyltransferase within the PARP family.^342–344^ Recent findings indicate that PARP9 preferentially recognizes and binds to viral double-stranded RNA ranging from 1.1 kb to 1.4 kb, acting as a noncanonical MAVS-independent RNA sensor during RNA virus infections^345^ (Fig. 4c).

##### RLRs signaling pathways

RIG-I signaling mechanisms are currently the most extensively studied among RLRs Canonically, RIG-I can be activated by short double-stranded RNA ( < 300 bp) and 5’-triphosphate single-stranded RNA.^346^ Upon binding to viral RNA at the CTD domain, RIG-I undergoes conformational changes that release the CARDs from RD repression, enabling interaction with its adaptor protein, mitochondrial antiviral-signaling protein (MAVS). This interaction activates the IRF3/7 and NF-κB pathways, leading to the expression of type I IFN and other pro-inflammatory cytokines.^340,347,348^ Notably, Liu et al. identified a novel RNA sensor, Gasdermin B (GSDMB), which shares similar characteristics with RLRs in activating ISG expression and downstream inflammation, significantly expanding our understanding of RLRs-dependent innate immune signaling^273^ (Fig. 4c).

##### PARP9 signaling pathways

PARP9 identifies and binds to viral dsRNA from reovirus in the cytoplasm, which triggers the recruitment and activation of phosphoinositide 3-kinase (PI3K) and AKT3 pathway, which occurs independently of MAVS. This activation of PI3K/AKT3 pathway subsequently phosphorylates IRF3/7, resulting in the production of type I interferon^345,349^ (Fig. 4c).

##### Role of RLRs signaling pathways in CVDs

RIG-I-mediated inflammation has recently garnered interest and is being actively investigated in cardiovascular diseases. Li et al. found that RLRs stimulation in human cardiac cells led to significant pro-inflammatory cytokines expression in fibroblasts, suggesting a pathogenic role for RIG-I in heart disease.^350^ Recent bioinformatics analysis identified RIG-I as a key gene in ischemic heart failure progression, with high RIG-I staining observed in human heart failure samples by immunohistochemistry.^351^ Indeed, RIG-I is expressed in intimal macrophages in human atherosclerotic lesions, and IFN-γ enhances its expression in macrophages, highlighting its role in atherosclerosis.^352^ In addition, RIG-I activation induced endothelial dysfunction by ROS accumulation and pro-inflammatory cytokines release during atherogenesis.^353^ Considering the critical role of innate immunity and inflammation in CVDs, along with RIG-I’s established involvement in cardiac reprogramming, investigating RIG-I’s function in the cardiovascular system warrants further study.

##### *Role of PARP9 signaling pathways in CVDs*

Bioinformatics analysis pinpointed PARP9 as a key gene with significant clinical diagnostic potential. Subsequent in vivo studies revealed that pirfenidone attenuated Ang II-induced myofibroblast differentiation and fibrosis by decreasing PARP9 expression triggered by Ang II.^354^ Consistently, PARP inhibition prevented the cardiac hypertrophy and contractile dysfunction in pressure overload-induced heart failure.^355^ Also, PARP inhibition could offer a promising new therapeutic approach to prevent postinfarction myocardial remodeling.^356^ However, the role of PARP9 in detecting cytoplasmic dsRNA and facilitating type I interferon production in relation to CVDs remains poorly understood.

PARP9 has been shown to be involved in atherogenesis. The PARP9 - PARP14 network, revealed through proteomics screening in cultured macrophages, demonstrated a notably stronger association with the human coronary artery disease gene module than with other cardio-metabolic diseases. Immunohistochemistry results confirmed that macrophages are a major source of PARP9 and PARP14 in human atherosclerotic lesions.^344^ Furthermore, inhibition of PARP with INO-1001 treatment markedly reduced atherosclerotic lesion development, as indicated by mitigated inflammatory reactions within the lesion.^357^The molecular mechanisms underlying these effects of PARP9 on vascular inflammation have yet to be thoroughly investigated.

### DNA sensor-dependent innate immune signaling

#### DNA sensors

##### cyclic GMP-AMP synthase (cGAS)

cGAS belongs to the ancient cGAS/DncV-like nucleotidyltransferase (CD-NTase) protein family, which produces various cyclic oligonucleotide second messengers in response to DNA, whether exogenous (from bacteria and viruses) or endogenous (from dying cells and damaged mitochondria).^358,359^ Upon recognizing DNA, cGAS dimers form ladder-like networks and phase-separated structures.^360^ These spatially restricted higher-order assemblies of cGAS-DNA on longer DNA stretches are crucial for biological functions. This mechanism prevents erroneous activation of cGAS by short or limited dsDNA, serving as an effective built-in immune checkpoint.^360^ STING (also known as MITA), consists of a short cytosolic N-terminal segment, a four-span transmembrane domain, a connector region, and a cytosolic ligand-binding domain (LBD) with a C-terminal tail (CTT).^360,361^ It is capable of binding the second messenger 2'3’ cyclic GMP-AMP (cGAMP), which is synthesized by cGAS.^362^ Upon binding to cGAMP produced during cGAS activation, STING undergoes conformational changes and forms a domain-swapped homodimer to initiate downstream signaling^363^ (Fig. 4d).

##### DEAD-box helicase 41(DDX41)

DDX41 is part of the DEAD-box protein family which consists of ATP-dependent RNA helicases, characterized by the conserved motif Asp-Glu-Ala-Asp (DEAD). Despite of the DEAD motif, DDX41 also contains other conserved domains, such as a helicase C-terminal domain and sites for ATP binding and hydrolysis. It has been reported that the helicase DDX41 functions as a DNA sensor by recognizing viral DNA.^364^ Additionally, DDX41 recognizes bacterial secondary messengers, including cyclic di-GMP (c-di-GMP) or cyclic di-AMP (c-di-AMP), to activate innate immune response^365^ (Fig. 4d).

#### cGAS and *stimulator of interferon genes (*STING)-dependent pathway

##### cGAS and STING-dependent pathway

cGAS undergoes conformational changes and synthesizes cGAMP (2’,5’-cyclic GMP-AMP dinucleotide) from guanosine 5´-triphosphate (GTP) and adenosine 5´-triphosphate (ATP) upon recognizing cytosolic dsDNA.^362^ cGAMP binds to STING, prompting STING to move from the ER to the ER-Golgi intermediate compartment (ERGIC) and the Golgi apparatus through the canonical ER-to-Golgi transport via COPII vesicles. Once STING reaches the ERGIC and Golgi apparatus, it recruits TBK1, which then undergoes self-phosphorylation and activates the transcription factors IRF3 and NF-κB pathways, thereby increasing the expression of type I IFN, ISGs expression and even IL-17 production.^366,367^ Notably, Liu et al. uncovered a crucial mechanism where GSDMB interacts with the C-terminus of STING, mediating STING’s translocation to the Golgi, significantly promoting downstream cascade^362^ (Fig. 4d).

##### *DDX41-dependent pathway*

DDX41 utilizes its DEAD domain to identify double-stranded DNA (dsDNA). Once the ligand is recognized, DDX41 associates with STING, which in turn activates the NF-κB and IRF3, ultimately leading to the production of type I interferon and other inflammatory genes.^368,369^ Moreover, Bruton’s tyrosine kinase (BTK) enhances DDX41 activation by phosphorylating the HELICc domain, thereby improving its binding to STING^370^ (Fig. 4d).

##### Role of cGAS and STING-dependent pathway in CVDs

The release of DAMPs, such as cytosolic DNA from necrotic tissue, following cardiac cell injury and death induced by MI, initiates the cGAS-STING pathway. Cao et al. observed MI-induced activation of the cGAS-STING pathway in mice, with significant upregulation of its transcriptional targets such as CXCL10, interferon-induced protein with tetratricopeptide repeats 1 (IFIT1), IFIT3, and IRF7.^371^ Inactivating cGAS signaling promoted myocardial repair by enhancing cardiac angiogenesis, reparative macrophage transformation, and myofibroblast transformation, and significantly reduced infarct size in mice with myocardial IRI.^371,372^ Pressure-overload induced HF, characterized by cardiac hypertrophy and fibrosis, was associated with elevated expressions of type I IFN and STING. Remarkably, mice deficient in STING exhibited improved cardiac function, with alleviated cardiac dysfunction and fibrosis, highlighting a direct role of cGAS-STING pathway in HF pathogenesis.^373^ Strikingly, Luo et al. found that doxorubicin-induced cardiac endothelial dysfunction via the cGAS-STING pathway modulated NAD homeostasis and mitochondrial bioenergetics in cardiomyocytes,^374^ indicating an intricate role of the cGAS-STING pathway in cell-cell crosstalk.

Cytosolic DNA from damaged cells and extracellular vesicles contributes to vascular inflammation, particularly in atherosclerosis.^375^ Activation of the cGAS-STING pathway in endothelial cells increases the expression of adhesion molecules, such as VCAM-1 and ICAM-1, aiding infiltration of innate immune cells into the arterial wall and contributing to the formation of early atherosclerotic lesions.^376,377^ However, activation of the cGAS-STING pathway in macrophages specifically enhances lipid uptake and foam cell formation, which are critical factors driving atherosclerosis.^377^ Furthermore, genetic or pharmacological suppression of STING in macrophages downregulates inflammatory cytokine expression, thereby mitigating atherosclerosis progression in mice.^378^ Therefore, targeting this pathway may illuminate strategies to prevent the progression of atherosclerotic lesions.

##### Role of DDX41-dependent pathway in CVDs

While current studies have not widely investigated the direct role of DDX41 in CVDs, there is emerging evidence suggesting its potential significance.^379^ Mutations in DDX41 have been associated with CVDs.^380^ The DDX41/cGAS/STING-mediated interferon inflammation has been linked to an increased risk of adverse outcomes in coronary artery disease due to the deletion of myocyte enhancing factor 2 (MEF2).^381^ These findings suggest that DDX41 may play a role in the regulation of cardiovascular conditions, highlighting the urgent need for further research into its clinical applications.

#### Absent in melanoma 2 (AIM2)-like receptors (ALRs) - dependent innate immune signaling

##### ALRs

ALRs are cytosolic and nuclear DNA sensors detecting bacterial and viral DNA, composed of an N-terminal PYD domain and one or two C-terminal hematopoietic expression, interferon inducibility, nuclear localization (HIN200) domain for DNA-binding.^382^ Similar to NLRs, the PYD functions as the effector region, transmitting downstream signals to the cellular machinery. Several ALR family members, including the IFN-inducible protein 16 (IFI16), AIM2 and IFI207, have been well characterized and implicated in the pathogenesis of various innate immune-related diseases^383–385^ (Fig. 4e).

##### ALRs-dependent signaling pathways

AIM2, the first identified ALR family protein for innate immune signaling, detects long dsDNA via its hematopoietic interferon-inducible nuclear antigens with HIN200 domains.^382^ Upon binding with a double-strand DNA (dsDNA), AIM2 interacts with the adapter protein ASC, whose CARD domain interacts with the CARD domain of pro-caspase-1 to form inflammasomes, leading to the release of mature IL-1β and IL-18.^386,387^ Another ALR, IFI16, also functions as a dsDNA sensor, inducing type I IFN expression and activating inflammasomes in a similar manner.^388^ Additionally, IFI207 co-localizes with active RNA polymerase II (RNA Pol II) and IRF7 in the nucleus, enhancing the induction of IRF7-dependent gene expression^383^ (Fig. 4e).

##### ALRs-dependent signaling pathways in CVDs

Studies have shown that ALRs-dependent signaling pathways contributes to both cardiac and vascular inflammation.^389^ For instance, the AIM2 inflammasome contributed to chronic inflammation in human and murine failing hearts, and its inactivation by probenecid improved outcomes in pressure overload-induced HF in rats.^390^ Also, AIM2 inflammasome activation is implicated in the proliferation of cardiac M1 macrophages and the expansion of infarct areas post-MI.^391^ In contrast, inactivation of the AIM2 inflammasome by Rg1 significantly reduced cardiac fibrosis and macrophage polarization, highlighting its potential as a crucial regulator of cardiac inflammation.^392^ In vascular inflammation, the AIM2 inflammasome is constitutively expressed in the healthy arterial wall but can become over-activated under atherosclerosis conditions.^393^ Moreover, the injection of AIM2 inflammasome agonist in ApoE^-/-^ mice resulted in impaired endothelium-dependent vasodilation, increased endothelial cells apoptosis, enhanced endothelium permeability, and elevated adhesion molecule expression.^394^ Additionally, AIM2 inflammasomes promoted MMP2 expression through the TGF-β/Smad signaling pathway in VSMCs.^395^ Genetic or pharmacological inactivation of AIM2 reduced levels of IL-1β and IL-18 in the necrotic core and destabilized atherosclerotic plaques.^396^ Apart from AIM2, IFI16 protein promotes inflammation in endothelial cells by activating of p38 MAPK and NF-κB p65, thereby contributing to vascular inflammation.^396^

#### The complement system-dependent pathways

##### Classical pathway

The classical pathway of the complement system is considered as the key bridge between the innate and adaptive immune systems. It is initiated by antigen-antibody complexes that involve IgM-class antibodies or specific IgG antibody sub-classes.^13^ This process begins when C1q, along with the serine proteases C1r and C1s, assembles into the macromolecular C1 complex, which then binds to the Fc region of complement-fixing antibodies.^397^ Notably, C1q can bind directly to various ligands - including C-reactive protein, DNA, annexins A2, and A5 - independently of IgM or IgG, thereby triggering the complement cascade.^398,399^ Upon binding antigen-antibody complexes, each C1 complex must bind to at least two constant regions of antibodies to establish a stable C1q-antibody interaction. This interaction induces a conformational change in one of the C1r proteins, activating and converting it into an active serine protease enzyme. Subsequently, the activated C1s cleaves C4 and C2 into larger fragments (C4b and C2a) and smaller fragments (C4a and C2b), respectively.^400,401^ The larger fragments associate with the formation of a C4bC2a complex on pathogenic surfaces, which acts as a C3 convertase, converting C3 into its enzymatically active forms: C3a and C3b. Specifically, the C3 convertase cleaves C3 into the anaphylatoxin C3a and the opsonin C3b^402^ (Fig. 4f).

##### Lectin pathway

In contrast to the antigen-antibody complex dependency of the classical pathway, the lectin pathway utilizes lectins such as collectins and ficolins as PRRs to recognize specific carbohydrate components.^403^ The lectin pathway of complement activation activates a C3 convertase (4bC2a) like the classical pathway, despite differences in their initiation processes. Several PRRs, including mannose-binding lectin (MBL) in the collectin family (collectin-10 and collectin-11) and ficolins (ficolin-1, ficolin-2, and ficolin-3), have been identified as specific receptors of the lectin pathway.^404,405^ These proteins share a collagen-like triple helix linked to a carbohydrate recognition structure.^403,406–408^ MBL is constitutively expressed in the liver and secreted into the plasma to recognize carbohydrate PAMPs on bacteria, viruses, and parasites.^409,410^ It forms a complex with MASPs (MBL-associated serine proteases)-1, -2, and -3, which are functionally and structurally similar to C1s and C1r of the C1 complex in the classical pathway.^411–413^ Among these three MASP subtypes, MASP-2 is recognized as the primary initiator of the MBL pathway.^414,415^ When MBL binds to pathogenic surfaces, it activates the associated MASPs, leading to the cleavage of C2 and C4 and the formation of the C3 convertase C4bC2a^416^ (Fig. 4f).

##### Alternative pathway

The alternative pathway of complement activation begins with the generation of C3(H2O), facilitating rapid immune responses against exogenous pathogens or endogenous damaged cells, independently of antibody-antigen complexes.^417^ After C3 undergoes spontaneous hydrolysis to form C3(H2O), this molecule binds to factor B, allowing factor D to cleave factor B into Bb and Ba, thereby creating the initial C3 convertase, C3(H2O)Bb.^418^ This C3 convertase then cleaves C3 into C3b and C3a. C3b subsequently binds to factor B, which is then activated by factor D to form the main C3 convertase, C3bBb.^419^ Properdin (Factor P) stabilizes this complex, amplifying the alternative pathway of complement activation.^420^ In addition to this “tickover” pathway, which involves the above four serum components—C3, factor B, factor D, and properdin—two other initiation modes have been identified. One of these is initiated by properdin, while the other is triggered by proteases such as thrombin and kallikrein^421^ (Fig. 4f).

##### The complement system-dependent pathways in CVDs

The complement system is implicated in the pathogenesis and development of various CVDs. Signs of complement activation are commonly observed in infarcted myocardium, failing hearts, and atherosclerotic arterial walls. For example, high-fat diet elevates plasma and local aortic complement expression levels, including C1q, C1s, C3, C4, and C9.^422–424^ C3-derived leukotactic fragments increased neutrophil infiltration in ischemic rat heart tissue and elevated plasma C3 levels correlate with the severity of atherosclerosis.^425,426^ Treatment with C3 inactivator reduced C3-derived leukotactic activity in the infarcted myocardium.^427^ Additionally, deposition of complement activation products C3d and C5b-9 was observed in ischemic human heart tissue, with more intense complement activation in patients who underwent reperfusion therapy or experienced reinfarction.^428^ Conversely, the absence of human complement components C1q in the classical pathway was associated with a higher incidence of cardiovascular events.^429^ Complement C1q reduces early atherosclerosis in Ldlr^-/-^ mice by clearing apoptotic cells.^430^ Also, lectin pathway is the key driver of tissue damage in CVDs.^431–433^ Mice lacking MBL or treated with an anti-MBL monoclonal antibody showed significantly reduced infarct size and cardiac inflammation.^432^ Similarly, in thrombosis progression, the complement system acts as a central hub and can be activated through the classical, alternative, or lectin pathways, leading to the production of complement factors like C3a and C1q.^434^ Moreover, thrombin produced during coagulation not only promotes fibrin generation, but also directly activates components of the complement system, such as C3 and C5, enhancing the complement cascade reaction.^435^ Consequently, the interaction between the complement system and the coagulation cascade response amplifies inflammation, leading to thrombus formation and tissue damage.^436^

### Adaptive immune response

**Fig. 5:**
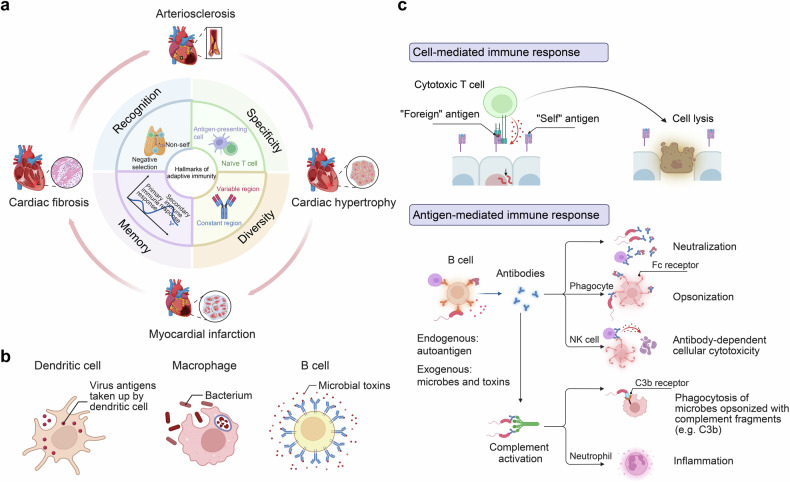
Adaptive immune response in CVDs. **a** Hallmarks of adaptive immunity in CVDs; **b** antigen-presenting cells (APCs); **c** Function of T lymphocytes and B lymphocytes. (Created with BioRender.com, https://BioRender.com/w51n151)

The adaptive immune system consists mainly of T and B lymphocytes (T and B cells) and APCs. It works together with the innate immune system to collectively defend the organism against foreign substances (“non-self”).^437,438^ In detail, antigenic molecules (either protein- or lipid-based) are phagocytosed and processed.^439^ Professional APCs like DCs, macrophages, and B cells process exogenous antigens via MHC class II molecules, presenting epitopes to CD4+ Th cells. In contrast, all nucleated cells process peptide fragments of endogenous antigens via the MHC class I pathway, presenting epitopes to CD8 + CTLs.^440,441^ Of note, not all antigens will provoke a specific immune response. For instance, individuals are constantly exposed to harmless foreign antigens, such as food proteins and dust components, as well as “self” antigens. The immune response to these antigens is highly suppressed, preventing potentially harmful processes to the host, known as tolerance.^442^ The adaptive immune system includes cell-mediated and antibody-mediated responses and is distinguished from the innate system by its specificity, diversity, memory, and self/non-self recognition (Fig. 5).

#### T lymphocytes

T cells are produced in the bone marrow and then travel through the bloodstream to the thymus, where they mature and acquire the “T” designation. There are three primary types of T cells: cytotoxic, helper, and suppressor T cells.^443,444^ Cytotoxic T cells destroy virally infected, damaged, and cancerous cells through cell-mediated immune responses and release cytokines to activate other immune cells.^445^ Helper T cells activate both cell-mediated and antibody-mediated immune responses by recognizing antigens bound to class II MHC molecules and initiating adaptive immune responses.^446^ Some helper T cells also differentiate into memory T cells after clearing an antigen, retaining antigen-specific characteristics to rapidly activate the adaptive immune system upon re-exposure.^447,448^

##### T lymphocyte-mediated adaptive immune response

Naïve T cells express either CD4 or CD8 molecules on their surface, classifying them as CD4+ or CD8 + T cells.^449,450^ They are activated into Th lymphocytes when they encounter APCs displaying antigens on MHC II molecules. Once activated, Th cells stimulate other immune cells and secrete cytokines to alert additional effector cells to the pathogenic threat. There are two primary subtypes of Th cells: Th1 and Th2. Th1 cells activate cytotoxic T cells through cytokine secretion, while Th2 cells stimulate naïve B cells to produce specific antibodies that target and eliminate pathogenic antigens.^451–453^ Unlike CD4 + T cells, CD8 + T cells are directly activated into cytotoxic T lymphocytes (CTLs) when interacting with antigens presented on MHC I molecules by APCs. These T cells, with diverse T cell receptors, achieve specificity through precise antigen-MHC recognition. After clonal selection, CD8 + T cells proliferate and target specific cells, inducing apoptosis.^454–456^ Additionally, CTLs can recognize and destroy infected cells before intracellular pathogens replicate and escape, thereby preventing further infection.^457,458^

##### T lymphocyte-mediated adaptive immune response in CVDs

T lymphocytes of adaptive immunity play significant roles in myocardial IRI mechanisms CVDs. Lymphopenia observed after primary percutaneous coronary intervention is linked to poor patient prognosis, possibly due to lymphocyte recruitment to ischemic myocardium.^459,460^ CD8 + T cells increase in aneurysmal aortic walls, promoting apoptosis and matrix remodeling by releasing IFN-γ.^461^ CD8-deficient mice exhibited reduced cardiomyocyte injury but had impaired necrotic tissue clearance, leading to inadequate scar formation and a higher risk of cardiac rupture.^460^ Furthermore, a non-cytotoxic CD8 + AT2R + T cell subset recruited to rat infarct areas post-MI, which produces IL-10 upon angiotensin II stimulation and promotes cardiac repair.^462^ In atherosclerosis development, *ApoE*^−/−^ *Cd8*^−/−^ mice showed no difference in plaque size compared to *ApoE* − /− controls. However, *ApoE* − /− *Cd4* − /− mice had significantly increased early lesions, and early depletion of CD4 + T cells accelerated atherosclerosis, indicating that early CD4 + T cell-mediated responses are largely protective against the disease.^6,463–465^ In addition, CD4 + T cells activate macrophages and induce smooth muscle cell apoptosis by secreting Th1 cytokines and Th2 cytokines.^461^ Tregs are also crucial for myocardial repair. Their depletion resulted in larger infarcts, increased local inflammation, reduced collagen deposition, and impaired survival.^466–468^ These effects may be due to Treg-derived cytokines like IL-10 and TGF-β.^469,470^

#### B lymphocytes

B cells originate in the bone marrow, naming them “B”. Subsequently, they mature into specialized adaptive immune cells. Upon stimulation by Th2 cells, naïve B cells differentiate into antibody-secreting plasma cells.^471,472^ Generally, antibodies produced by activated B cells perform six functions: 1) Neutralizing pathogens or toxins; 2) Agglutinating pathogens to aid in clearance; 3) Opsonizing pathogens to attract phagocytic cells; 4) Activating complement by binding to pathogens, which starts the complement cascade; 5) Enhancing cell-mediated immune responses by recruiting cytotoxic cells, leading to antibody-dependent cell-mediated cytotoxicity (ADCC); 6) Inducing degranulation in granulocytes.^473,474^

##### B lymphocyte-mediated adaptive immune response

Naïve B cells form a diverse population with numerous B cell receptors (BCRs) that bind and internalize foreign antigens.^475^ After processing the antigens, B cells present them on MHC II molecules to be recognized by Th2 cells. Plasma cells then rapidly produce and release significant quantities of antibodies that match the antigen recognition pattern of the BCRs into the bloodstream.^476,477^ Some activated B cells develop into memory cells to respond if the same antigen reappears. Antibody binding marks invading pathogens for destruction, primarily by facilitating their uptake by phagocytic cells.^471^

##### B lymphocyte-mediated adaptive immune response in CVDs

Studies in mice have shown that B cell accumulation occurs in the infarcted myocardium following myocardial infarction.^139^ Typically, B cells produce natural IgM antibodies against non-myosin heavy chain II, which can damage the heart because their response in generating specific antibodies is delayed.^139^ Furthermore, mature B lymphocytes selectively produced Ccl7 and induce the mobilization and recruitment of Ly6C^hi^ monocyte to the heart after myocardial infarction, leading to decreased myocardial function.^478^ It’s important to note that not all B cells are harmful. A subset of B cells that produce IL-10 may aid in resolving inflammation and promoting heart recovery after myocardial infarction.^479^ During arrhythmia development, autoantibodies alter the function of cardiac ion channels, significantly impacting cardiac electrical activity.^206^ For example, anti-Ro/SSA antibodies target L-type and T-type calcium channels, inhibit calcium currents, and affect sodium currents in the sinoatrial node and atrioventricular node.^207,208^ Anti-SSA antibodies also target K11.1V11.1 K channels (hERG), inhibiting potassium currents involved in rapid repolarization^211,213^

### Other signaling pathways in immune response

#### Hippo/YAP pathway

**Fig. 6:**
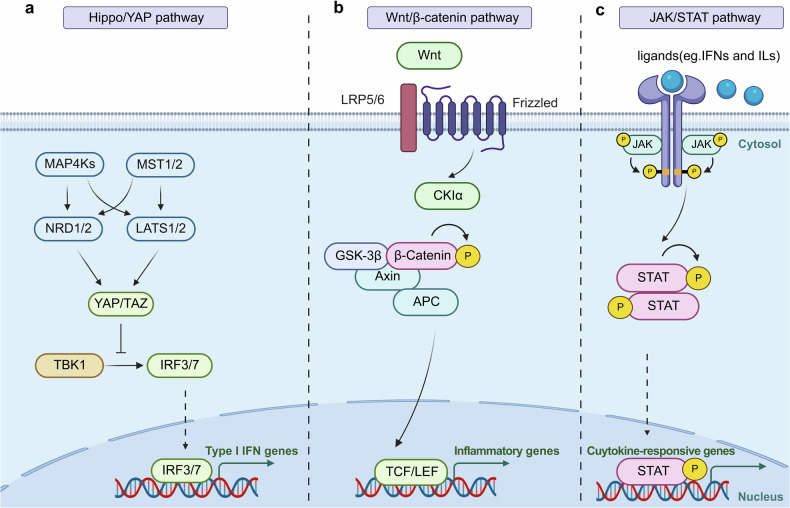
Other immune signaling pathways in CVDs. **a** Hippo/YAP pathway, **b** Wnt/β-catenin pathway, **c** JAK/STAT pathway. MAP4Ks Mitogen-Activated Protein Kinase Kinase Kinase Kinase, MST1/2 Mammalian Sterile 20-like kinases 1/2, LATS1/2 Large Tumor Suppressor Kinase 1/2, NRD1 Nuclear pre-mRNA Down-regulation 1, YAP/TAZ Yes-Associated Protein/Transcriptional coActivator with PDZ-binding motif, IRF3/7 Interferon Regulatory Factor 3/7, LRP5/6 Low-density lipoprotein Receptor-related Protein 5/6, GSK-3β Glycogen Synthase Kinase 3 beta, JAK Janus Kinase, STAT Signal Transducer and Activator of Transcription, IFN Interferon, IL Interleukin, TBK1 TANK Binding Kinase 1, CKIα Casein Kinase I alpha (Created with BioRender.com, https://BioRender.com/k49g175)

The Hippo-YAP signaling pathway is highly conserved among various species.^480^ Recent findings have revealed that components of the Hippo-YAP pathway, including MST1/2 (mammalian Ste20-like kinases 1/2), MAP4Ks, LATS1/2, NDR1/2, and YAP/TAZ are crucial regulators of innate immune responses.^481^ YAP has been shown to suppress interferon response by targeting TBK1 and disrupting its interaction with IRF3.^482^ Furthermore, Wang et al. showed that YAP can interact with IRF3, preventing its dimerization and nuclear translocation, thereby reducing IFN-β and ISG production.^483^ However, the Hippo/YAP pathway can be activated downstream of TLRs, ultimately amplifying the NF-κB signaling.^484^ This indicates that the Hippo/YAP pathway has a complex role in modulating innate immune response (Fig. 6a).

##### Role of Hippo/YAP pathway-mediated immune response in CVDs

The involvement of the Hippo/YAP pathway in modulating inflammation and immune responses in CVDs has gained significant attention recently.^485,486^ Emerging studies indicate that MI triggered the activation of Hippo pathway kinases, leading to enhanced caspase activation and elevated levels of phosphorylated YAP, which subsequently caused cardiomyocyte apoptosis.^487,488^ Importantly, the YAP/TEAD1 complex in cardiomyocytes modulated the expression of TLR genes during MI.^489,490^ The cardiomyocyte-specific overexpression of YAP has been shown to improve cardiac function and survival after MI in mice.^491^ Meanwhile, this process was accompanied by the reduced expression of TLRs, particularly TLR2 and TLR4, suggesting the upstream regulation of Hippo-Yap pathway in TLRs-dependent immune response.^489,492^ Furthermore, activation of YAP in tissue samples from hypertrophic cardiomyopathy and in TAC-induced failing hearts from mice, suggesting that the Hippo/YAP signaling pathway plays a role in the development of cardiac hypertrophy and HF.^493^ Cardiomyocyte-specific inhibition of MST1 reduced neutrophil and macrophage infiltration in the heart and suppressed the release of inflammatory cytokines in the progression of diabetic cardiomyopathy.^494^

The inflammatory response regulated by the Hippo/YAP pathway has been linked to the process of vascular remodeling. In the atherosclerotic arteries, prominent YAP/TAZ staining was observed in the endothelium, media layer, and intimal hyperplastic plaque.^495^ Activation of YAP/TAZ triggered the expression of pro-inflammatory mediators, including IL-6, IL-8, and CCL2, and enhanced monocyte adhesion to endothelial cells, implying that endothelial YAP/TAZ activation plays a role in the early stages of atherosclerosis.^495,496^ Moreover, in the aortas of hypertensive mice induced by Angiotensin II, treatment with the YAP/TAZ inhibitor decreased the infiltration of inflammatory cells and the production of pro-inflammatory cytokines.^497^

#### Wnt/β-catenin pathway

The Wnt signaling pathways consist of both canonical and noncanonical routes. The canonical Wnt/β-catenin pathway is characterized by the translocation of β-catenin to the nucleus, whTCFere it activates target genes through T-cell factor/lymphoid enhancer-binding factor (TCF/LEF).^498^ This pathway is structured into four main components:the extracellular, membrane, cytoplasmic, and nuclear segments. Extracellular signals are primarily mediated by Wnt ligands, such as Wnt3a, Wnt1, and Wnt5a. The membrane segment mainly includes Wnt receptors, including Frizzled (a seven-transmembrane receptor) and lipoprotein receptor-related protein (LRP) 5/6. The cytoplasmic segment encompasses key proteins like β-catenin, glycogen synthase kinase-3β (GSK-3β), and casein kinase I (CK1). In the nuclear segment, β-catenin translocates into the nucleus, where it interacts with TCF/LEF family members to regulate the expression of downstream target genes.^499,500^ Recent studies reveal that the WNT/β-catenin pathway and TLR-mediated NF-κB signaling pathways interact, influencing each other’s functions.^501^ The Wnt/β-catenin pathway plays a dual role, exhibiting both pro-inflammatory and anti-inflammatory effects, which are partly due to its modulation of the NF-κB pathway. Similarly, the TLR signaling pathway also serves as a either positive or negative regulator of Wnt/β-catenin signaling^501,502^ (Fig. 6b).

##### Role of Wnt/β-catenin pathway-mediated immune response in CVDs

Growing evidence suggests that Wnt signaling is activated during the pathological progression of MI injury, as evidenced by elevated expressions of Wnt ligands, including *Wnt2*, *Wnt4*, *Wnt10b*, and *Wnt11* after MI.^503^ Wnt5a has been identified as specifically expressed in cardiomyocytes, where it plays a key role in triggering the release of pro-inflammatory cytokines after MI.^504,505^ Endogenous Wnt pathway inhibitors, such as SFRPs, have been shown to protect against MI by preventing leukocyte activation and cytokine production.^506^ The activation of β-catenin in cardiomyocytes led to increased levels of inflammatory markers such as TNF-α, p-NF-κB, and IL-8, and enhanced the nuclear accumulation of NF-κB. This indicates that β-catenin contributes to post-MI inflammation by activating the NF-κB pathway.^507^ Dysregulated Wnt/β-catenin activation has also been observed in the pathogenesis of pressure overload-induced cardiac hypertrophy cardiac hypertrophy in mice, while interruption of Wnt signaling was found to attenuatecardiac dysfunction.^508^

Canonical Wnt/β-catenin signaling has been implicated in the pathogenesis of atherosclerosis. The transcriptional level of Wnt5a has been reported to be induced by oxLDL in macrophage-rich areas of human atherosclerotic plaques, where Frizzled 5 and Wnt5a cooperated to promote the expressions of pro-inflammatory markers to further amplify the local inflammation.^509,510^ Additionally, Wnt5a has been shown to exert a relatively long-lasting and sustained impact on the NF-κB pathway, thereby enhancing the innate immune response in the atherosclerotic plaques.^511^ Consistently, the Wnt receptor LRP5 expression was elevated in macrophages within advanced plaques compared to early ones, suggesting a crucial role of Wnt-mediated regulation of macrophages in the pathophysiology of atherosclerosis.^512^ Furthermore, myeloid β-catenin deficiency could exacerbate atherosclerosis in mice.^513^ These evidence demonstrate that targeting Wnt/β-catenin pathway in macrophages within plaques could offer a promising strategy for treating atherosclerosis.

#### JAK/STAT pathway

The JAK/STAT pathway includes four JAK proteins - JAK1, JAK2, JAK3, and TYK2 - and seven STATs proteins - STAT1, STAT2, STAT3, STAT4, STAT5A, STAT5B, and STAT6.^514^ The canonical signaling pathway is initiated when cytokines interact with their specific receptors, which consist of different chains that undergo oligomerization. This oligomerization of the cytokine receptor leads to the separation of the intracellular segments, distancing the receptor-bound JAKs from one another.^515,516^ This process removes the constitutive inhibition, resulting in JAK activation. The activated JAKs then phosphorylate the intracellular domain of cytokine receptors, creating selective binding site for STAT proteins.^517^ STATs are subsequently phosphorylated on tyrosine residues by JAKs, leading inactive STAT monomers to undergo conformational changes, enabling them to form active homodimers, heterodimers, or tetramers. These activated STAT complexes then translocate to the nucleus, where they act as transcription factors to regulate downstream target gene expression.^518^ The JAK/STAT signaling pathway is essential for coordinating immune and inflammatory responses, as numerous inflammatory cytokines, such as IFNα/β/γ and IL-4/6/11, activate this pathway^519^ (Fig. 6c).

##### Role of JAK/STAT pathway-mediated immune response in CVDs

The involvement of the JAK/STAT pathway in IR injury has been well investigated. McCormick et al. found that myocardial ischemia triggered enhanced STAT3 phosphorylation, which further augmented following reperfusion in rat.^520^ Using JAK2 inhibitor AG490, increased apoptosis and caspase-3 activity were observed in rat following I/R injury.^521^ Furthermore, overexpression of STAT3 protected mice against doxorubicin-induced cardiomyopathy.^522^ Similarly, cardiac-specific STAT3-deficient mice were more vulnerable to I/R-induced cardiac injury, as indicated by larger infarct areas and increased apoptosis after reperfusion, compared to wild-type controls.^523^ Furthermore, IL-6 and NF-κB activity are necessary for ischemic preconditioning and may act synergistically with the JAK/STAT pathway.^524,525^

The JAK/STAT pathway regulates the inflammatory processes in vascular cells, contributing to the development of atherosclerosis. JAK2/STAT3 pathway modulates arterial adventitia inflammation via crosstalk with NF-∣B pathway. Dotan et al. demonstrated that *Apoe*^−*/−*^ mice deficient in macrophage *Jak2* developed accelerated atherosclerosis.^526^ Additionally, An et al. found that STAT3/NF-∣B decoy oligodeoxynucleotides (ODNs) reduced atherosclerosis by modulating the STAT/NF-∣B signaling pathway in mice.^527^

## Multi-level regulatory signaling pathways/crosstalks in CVDs

### Epigenetic, post-transcriptional, post-translational modification regulatory mechanisms of immune response in CVDs

**Fig. 7:**
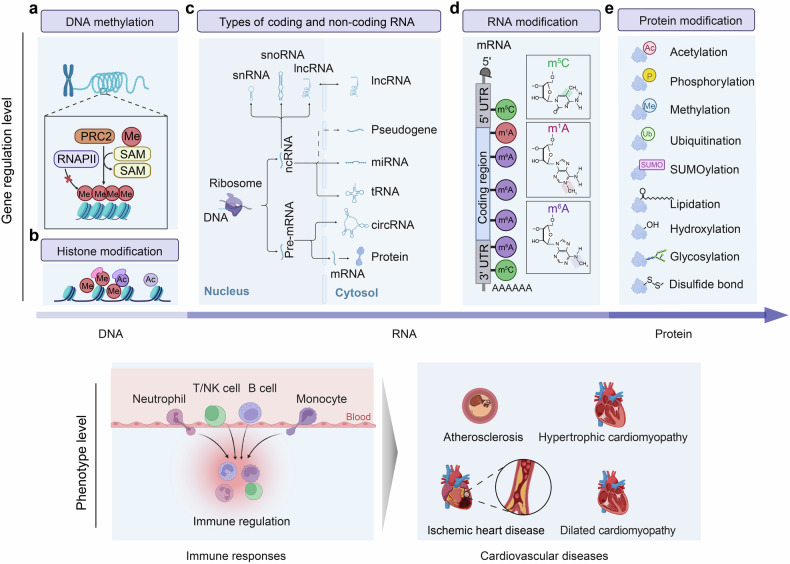
Epigenetic, post-transcriptional, post-translational modification regulatory mechanisms of immune response in CVDs. **a** DNA methylation; **b** Histone modification; **c** Types of coding or non-coding RNA; **d** mRNA modification; **e** Protein modification. NK cell Natural Killer cell, UTR Untranslated Region (Created with BioRender.com, https://BioRender.com/i78n158)

Epigenetic processes, including DNA methylation, histone modifications, non-coding RNA, RNA modifications, and post-translational modifications, mediate the diversity of gene expression patterns across different cells and tissues. These modifications establish a molecular framework through which environmental factors can impact gene expression. They play a crucial role in the activation and functional differentiation of immune cells and cardiomyocytes, thereby significantly influencing the development of CVDs (Fig. 7).

#### DNA methylation

DNA methylation acts as an annotation system for the genetic code, delivering essential instructions on when and how to read genetic information and control transcription. Unlike inherited genetic sequences, methylation patterns are formed through a programmed process that persists throughout development, leading to stable gene expression profiles.^528^ In terms of mechanism, DNA methyltransferases (DNMTs) covalently transfer a methyl group from S-adenosyl methionine to the C-5 position of cytosine, forming 5-methylcytosine (5mC).^529^

Emerging evidences has highlighted the role of DNA methylation in regulating immune cell functions,^530–532^ providing comprehensive insights into how DNA methylation affects immune cell behavior and contributes to CVDs. The CARDIA study discovered that the methylation risk score significantly improved the discrimination capacity for coronary artery calcification status compared to the cardiovascular health (CVH) score alone, and it was associated with the risk of incident coronary artery calcification 5–10 years later, independent of the cumulative CVH score.^533^ Shifting macrophage polarization from a pro-inflammatory (M1) to an anti-inflammatory (M2) state through epigenetic modifications could be a potential therapeutic strategy for conditions like atherosclerosis.^534,535^ In addition, DNMT1 regulates macrophage motility and mechanical properties by controlling cellular cholesterol accumulation and lipid homeostasis, affecting wound healing and macrophage chemotactic migration^536^ (Fig. 7a).

#### Histone modification

Histone modifications regulate chromatin structure and gene expression by chemically altering amino acids on histone tails, including acetylation, methylation, phosphorylation, ubiquitination, and sumoylation. These changes influence chromatin openness, controlling gene activation or silencing, and impacting cell differentiation, development, stress responses, and disease progression.^537–540^

Histone modifications significantly impact the occurrence and progression of CVDs by mediating immune regulation through mechanisms such as inflammation response, immune cell function, cardiac remodeling, and vascular function. Histone lactylation in monocytes promotes early activation of reparative gene expression, crucial for immune homeostasis and cardiac repair post-myocardial infarction, by regulating anti-inflammatory and pro-angiogenic activities.^541^ Inhibition of DYRK1A via histone modification, promotes cardiomyocyte cell cycle activation and cardiac repair after MI.^542^ Also, nucleophosmin1 recruits histone demethylase KDM5b to the TSC1 promoter, reducing H3K4me3 and TSC1 expression. This enhances mTOR-related inflammatory glycolysis and abolishes macrophage repair.^543^ Similarly, HDAC3 controls macrophage polarization and inflammation, with its deficiency leading to increased IL-4-induced polarization and atherosclerotic plaque size. Upregulated in ruptured plaques, HDAC3 inversely correlates with TGF-β1, and its knockdown reduces macrophage inflammation and pro-inflammatory mediators.^544^ Additionally, histone acetyltransferase pathways upregulate NADPH oxidase 5 in human macrophages during inflammation, potentially leading to excessive ROS production in atherosclerosis^545^ (Fig. 7b).

#### Non-coding RNA

Non-coding RNA (ncRNA) comprises RNA molecules that do not encode proteins but are essential for gene expression regulation, genome stability, post-transcriptional modification, and translation control.^546,547^ Present in eukaryotes, prokaryotes, and viruses, ncRNAs include microRNAs (miRNAs), long non-coding RNAs (lncRNAs), circular RNAs (circRNA), Piwi-interacting RNAs (piRNAs), and ribosomal RNAs.^548,549^ These molecules modulate gene function and cellular processes by interacting with DNA, RNA, and proteins.^550^

LncRNA has gained increased interest in the cardiovascular community for their ability to modulate cellular responses.^551^ For example, LncRNA-H19 regulates lipid metabolism and inflammation in ox-LDL-treated Raw264.7 cells via the H19-miR130b pathway, decreasing lipid accumulation and pro-inflammatory factors while increasing anti-inflammatory factors.^552^ The lncRNA MIAT is a newly identified regulator of cellular processes in advanced atherosclerosis, influencing the proliferation, apoptosis, and phenotypic transition of SMCs, as well as the pro-inflammatory characteristics of macrophages.^553,554^ The lncRNA SIMALR, suppresses inflammatory macrophage apoptosis via NTN1 (Netrin-1).^555^ LncRNA CCRR reduced infarct size and improved cardiac function by inhibiting the secretion of proinflammatory factors through the suppression of TLR2 and TLR4.^556^ miRNAs also play a role in the progression of CVDs. Overloaded hearts in mice revealed that miR-21 is crucial for macrophage polarization towards an M1-like phenotype, miR-21 primarily determined macrophage-fibroblast communication, promoting the transition from quiescent fibroblasts to myofibroblasts.^557^ miR-214 shows the highest induction in response to Ang II-mediated hypertension. Global deletion of miR-214 prevents Ang II-induced periaortic fibrosis, vascular stiffening, and T-cell recruitment. Thus, *miR-214*−/− mice are shielded from endothelial dysfunction and oxidative stress, underscoring miR-214’s involvement in pathological perivascular fibrosis through T cell recruitment and the release of pro-fibrotic cytokines.^558^ Moreover, circRNA_002581 can sponge miR-122, a micRNA that targets genes involved in inflammation. By sponging miR-122, circRNA_002581 promotes a pro-inflammatory macrophage phenotype, which contributes to vascular inflammation and cardiac hypertrophy.^559^ circRNA_010567 promotes the activation of the NF-κB pathway by sponging miR-141, which targets a key inhibitor of NF-κB. In M2 macrophage-derived small extracellular vesicles (SEVs), circUbe3a promotes myocardial fibrosis by targeting the microRNA-138-5p/RhoC axis, driving cardiac fibroblast proliferation, migration, and phenotypic transformation, thereby worsening myocardial fibrosis after MI^560^ (Fig. 7c).

#### RNA modification

RNA modification is essential for cellular function by regulating gene expression.^561,562^ Key modifications include m6A (N6-Methyladenosine), which methylates adenine residues and is added by the methyltransferase Like 3 METTL3-METTL14 complex and removed by demethylases like fat mass and obesity-associated protein (FTO) and AlkB homolog 5 (ALKBH5); m5C (5-Methylcytosine), which methylates the fifth carbon of cytosine and is added by methyltransferases such as DNA methyltransferase 2 (DNMT2) or NOP2/Sun RNA methyltransferase family member 2 (NSUN2); and m7G (7-Methylguanylate), which is capped at the 5’ end of mRNA by capping enzymes. These modifications influence various aspects of gene expression, including splicing, maturation, transport, stability, and translation.^563,564^

Dynamic alterations in RNA modifications across different types of RNA are crucial for the development and functioning of the immune system.^565^ *METTL3*-deficient dendritic cells display immature characteristics and extend allograft survival.^566,567^ Also, METTL3-dependent N6-methyladenosine modification of Braf mRNA amplifies the macrophage inflammatory response and accelerates atherosclerosis in mice.^568^ Similarly, METTL14 exacerbates endothelial inflammation and atherosclerosis by increasing N6-methyladenosine modifications on FOXO1 and mediates the inflammatory response of macrophages through the NF-kB/IL-6 signaling pathway.^569,570^ Seven key m6A regulators - Wilms tumor 1 associated protein (WTAP), Zinc Finger CCCH-Type Containing 13 (ZCH3H13), YTH domain-containing protein 1 (YTHDC1), Fragile X Messenger Ribonucleoprotein 1 (FMR1), FTO, RNA Binding Motif Protein 15 (RBM15), and YTH N6-methyladenosine RNA Binding Protein 3 (YTHDF3)—could serve as novel biomarkers for the precise diagnosis of ischemic cardiomyopathy (ICM).^571^ In addition, Kun Wang et al. reveal that piRNA-mediated m5C methylation is involved in the regulation of cardiomyocyte necroptosis. Heart necroptosis-associated piRNA (HNEAP) regulates cardiomyocyte necroptosis by inhibiting the m5C methylation of Atf7 mRNA^572^ (Fig. 7d).

#### Post-translational modification

Post-translational modification (PTM) involves the covalent and enzymatic alteration of proteins after biosynthesis, impacting their function, localization, and cellular interactions.^573,574^ Key PTMs include phosphorylation, ubiquitination, and methylation.

Post-translational modifications like phosphorylation and polyubiquitination strongly regulate innate inflammatory responses by affecting the activation, translocation, and interaction of innate receptors and signaling molecules in response to harmful signals.^575–577^ In atherosclerosis, IL-8 binds to CXC motif chemokine receptor 2 (CXCR2) on neutrophils, promoting NET formation through Src, extracellular signal-regulated kinases (ERK), and p38 MAPK phosphorylation. This activates the TLR9/NF-κB pathway in macrophages, increasing IL-8 release and worsening disease, highlighting NETs as a therapeutic target.^578^ In obesity-induced cardiomyopathy, deleting or inhibiting doublecortin-like kinase 1 (DCLK1) in macrophages protects against high-fat diet-induced heart dysfunction, hypertrophy, and fibrosis by suppressing receptor-interacting serine/threonine-protein kinase 2 (RIP2) phosphorylation and inhibiting RIP2/TAK1-mediated inflammation.^579^ Also, TRAF6, downstream of IL-1β, ubiquitinates YAP at K252, enhancing its nuclear translocation and disrupting interaction with angiomotin, leading to increased macrophage infiltration and atherosclerotic lesions.^580^ WWP2, an E3 ubiquitin ligase, regulates cardiac fibrosis in non-ischemic cardiomyopathy by modulating the CCL5/Ly6chigh monocyte axis. WWP2 affects Ly6c high monocytes, promoting IRF7 mono-ubiquitination, nuclear translocation, and transcriptional activity, leading to CCL5 upregulation and reduced myofibroblast trans-differentiation.^581^ RNF5 inhibits cardiac hypertrophy by promoting STING degradation via K48-linked polyubiquitination, thereby reducing inflammation and immune responses.^582^ Additionally, interferon-stimulated gene 15 (ISG15) induces vascular damage in hypertension by promoting oxidative stress and inflammation, leading to endothelial dysfunction and vascular remodeling through post-translational modification (ISGylation) of macrophages.^583^ In ischemic myocardium, pharmacologically-induced hyper-O-GlcNAcylation enhances M2-like macrophage reparative activation. Myeloid knockdown of O-GlcNAcase, leading to hyper-O-GlcNAcylation, positively regulates M2-like activation and reduces post-MI hyper-inflammation.^584^

The regulatory mechanisms of epigenetic, post-transcriptional, and post-translational modifications are crucial for understanding immune responses in CVDs. These modifications—including DNA methylation, histone alterations, non-coding RNAs, and protein modifications—interact in complex ways to influence gene expression patterns that are vital for immune cell function and the progression of CVDs. Investigating these processes not only reveals the underlying biological mechanisms of CVDs but also opens innovative pathways for therapeutic interventions aimed at modulating these modifications to improve patient outcomes (Fig. 7e).

### Integration of key signaling pathway in CVDs with immune response

**Fig. 8:**
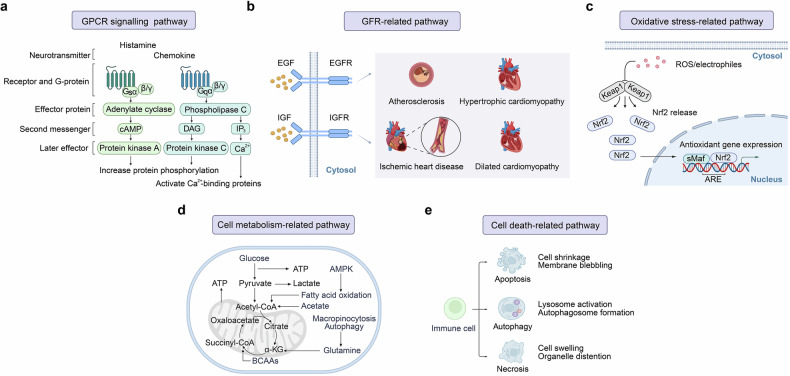
Integration of key signaling pathway in CVDs with immune response. **a** G protein-coupled receptor (GPCR) signaling pathway; **b** GFR-related pathway; **c** Oxidative stress-related pathway; **d** Cell metabolism-related pathway; **e** Cell death-related pathway. GPRC G Protein-Coupled Receptor, DAG Diacylglycerol, cAMP Cyclic Adenosine Monophosphate, IP3 Inositol Trisphosphate, GFR Growth factor receptor, EGF Epidermal growth factors, IGF Insulin-like growth factors, EGFR Epidermal growth factor receptors, IGFR Insulin-like growth factor receptors, sMaf Small Musculoaponeurotic Fibrosarcoma, Nrf2 Nuclear Factor Erythroid 2-Related Factor 2, ARE Antioxidant Response Element, ATP Adenosine Triphosphate, TCA Tricarboxylic Acid cycle, BCAAs Branched-Chain Amino Acids, TLR Toll-Like Receptor, IκB Inhibitor of kappa B, NF-κB Nuclear Factor Kappa-light-chain-enhancer of activated B cells, P50 Nuclear Factor NF-kappa-B p50 subunit, P65 Nuclear Factor NF-kappa-B p65 subunit (Created with BioRender.com, https://BioRender.com/p48m296)

Key signaling pathways in CVDs interplay with immune responses, which matters in CVDs. The crosstalk among those pathways highlights the importance of immune responses in CVDs (Fig. 8).

#### G protein-coupled receptor (GPCR)-signaling pathway

GPCR signaling pathways are intricate signaling processes initiated by GPCRs, a vast and diverse group of membrane receptors. Upon ligand binding to a GPCR, a conformational change occurs, which in turn activates an associated G protein by facilitating the exchange of GDP for GTP on the Gα subunit. This activation causes the G protein to dissociate into Gα and Gβγ subunits, both of which interact with various downstream effectors. These interactions produce second messengers, such as cyclic AMP (cAMP) or inositol triphosphate (IP3), which further propagate the signal. The signaling cascade ultimately leads to a wide range of cellular responses. GPCR pathways are fundamental for numerous physiological functions, including sensory perception, immune response, mood regulation, and metabolism.^585^

The GPCR signaling pathway modulates immune cell behavior and the expression of inflammatory mediators, playing a crucial role in maintaining immune system balance and ensuring appropriate inflammatory responses. This regulation is essential for orchestrating an effective immune response while preventing excessive inflammation that could lead to tissue damage.^586^ Chemokine receptors (CCR) are a large family of seven transmembrane domain GPCRs that are differentially expressed across various cell types.^587^ Single-cell RNA sequencing reveals that tissue-resident cardiac macrophages guide monocyte fate differently. Selective depletion of CCR2- or CCR2+ macrophages before myocardial infarction has distinct effects on left ventricular function, myocardial remodeling, and monocyte recruitment.^588^ Mechanistically, these CCR2- macrophages communicated with adjacent cardiomyocytes through focal adhesion complexes and were activated by mechanical stretch via a transient receptor potential cation channel subfamily V member 4 (TRPV4)-dependent pathway, regulating growth factor expression.^589^ Furthermore, CXCR7 meticulously regulates the dosage and signaling of adrenomedullin, a mitogenic peptide hormone essential for cardiovascular development. The loss of this decoy receptor leads to postnatal lethality due to abnormal cardiac development^590^ (Fig. 8a).

#### Growth factor receptor (GFR)-related pathway

GFR-related pathway involves cellular signaling processes that start with growth factors binding to their receptors on the cell surface. This binding triggers a cascade of intracellular events leading to various cellular responses, including proliferation, differentiation, migration, and survival. Growth factors, such as the epidermal growth factors (EGF) and the insulin-like growth factors (IGF), are proteins that interact with specific transmembrane receptors possessing intrinsic tyrosine kinase activity.^591^

The EGFR pathway mediates macrophage responsiveness to specific diseases, impacting cardiac function and remodeling after acute ischemic injury. Myeloid cell-specific EGFR deletion leads to increased cardiomyocyte hypertrophy and worsened cardiac function and repair after acute myocardial infarction, with decreased levels of pro-reparative mediators such as Vegfa and Il10, and reduced capillary density.^592^ In addition to EGFR, inflammatory cells secrete myeloid-derived growth factor (MYDGF) to aid tissue repair and regeneration following acute myocardial infarction. MYDGF levels rise in the left ventricular myocardium and blood plasma of pressure-overloaded mice, which enhances sarcoplasmic/endoplasmic reticulum Ca2+-ATPase 2a (SERCA2a) expression, reduces hypertrophy and dysfunction, and improves survival rates in both mice and patients with severe aortic stenosis.^593^ Furthermore, EGFR blockade in CD4 + T cells induces T cell anergy and reduces the development of atherosclerosis. EGFR inhibition decreases T cell proliferation, activation, and cytokine production, leading to reduced T cell infiltration in atherosclerotic lesions.^594^ Additionally, IL-35 promotes the survival of reparative CX3CR1+Ly6Clow macrophages, which in turn reduces cardiac rupture, improves wound healing, and attenuates cardiac remodeling after MI by enhancing α-SMA and collagen expression.^595^ Thrombospondin-1 (TSP1), a well-known inhibitor of angiogenesis, exerts its effects by interacting with cell surface receptors such as CD36 and CD47. TSP1 suppressed lymphangiogenesis and inhibited VEGF-C-induced AKT and eNOS activation in lymphatic endothelial cells (LEC). CD47 silencing in LEC prevented these effects, and Cd47 knockout mice showed reduced atherosclerosis and higher lymphatic vessel density.^596^ Insulin-like growth factor-1 (IGF1R) signaling in macrophages suppresses foam cell accumulation and reduces plaque vulnerability in atherosclerotic lesions. IGF1R-deficient macrophages showed enhanced pro-inflammatory responses and reduced lipid efflux, increasing atherosclerosis and plaque instability.^597^ Eosinophils and eosinophil cationic protein (ECP) promote SMC calcification and atherogenesis via the BMPR-1A/1B-Smad-1/5/8-Runx2 signaling pathway. Eosinophil deficiency in ΔdblGATA mice slowed atherogenesis, increased SMC content, and reduced calcification.^598^ TGF(transforming growth factor)-β activates Smad3 in macrophages, enhancing phagocytosis and anti-inflammatory transition. Smad3 knockout in macrophages leads to increased mortality, adverse remodeling, and impaired anti-inflammatory responses post-myocardial infarction.^599^ Fibroblast growth factor 10 (FGF10) coacervate injection significantly attenuated MI injury by preserving cardiac function, reducing inflammation and fibrosis, improving vascular stabilization, and activating phosphorylated fibroblast growth factor receptor (p-FGFR), PI3K/AKT, and ERK1/2 pathways more effectively than free FGF10 or heparin united FGF10^600^ (Fig. 8b).

#### Oxidative stress-related pathway

Oxidative stress-related pathways refer to the cellular signaling processes triggered by an imbalance between the production of ROS, reactive nitrogen species (RNS) and the cell’s antioxidant defenses. This imbalance leads to oxidative damage to proteins, lipids, and DNA, which can disrupt normal cellular function.^601^

In MI, mitochondria-targeted ROS scavenging mitigated impairments, enhanced myofibroblast function in vivo, and decreased mortality in mKO mice. These results underscore the crucial role of mitochondria in resolving inflammation and facilitating tissue repair through the modulation of efferocytosis and interaction with fibroblasts, holding significant potential for improving post-MI recovery and addressing other inflammatory conditions.^602^ The mitochondrial deacetylase Sirtuin3 (Sirt3) plays a crucial role in regulating metabolic and antioxidant functions linked to hypertension. Sirt3 depletion in hypertension leads to endothelial dysfunction, vascular hypertrophy, inflammation, and end-organ damage. Targeting Sirt3 expression has therapeutic potential for vascular dysfunction and hypertension^603^ (Fig. 8c).

#### Cell metabolism-related pathway

Cell metabolism-related pathways encompass biochemical processes that convert nutrients into energy and essential building blocks for cellular functions. Key pathways include glycolysis, the citric acid cycle, and oxidative phosphorylation, all of which generate ATP. The pentose phosphate pathway produces NADPH and ribose-5-phosphate for biosynthesis. Lipid metabolism processes fatty acids for energy storage and membrane synthesis. AMPK and mTOR signaling are among the best-understood metabolite-sensing and signaling pathways. These pathways regulate cellular energy balance, growth, and responses to environmental changes, supporting vital functions such as cell proliferation, differentiation, and survival.^604^

Dysregulation of cell metabolism can lead to diseases like cancer, CVDs, and metabolic disorders.^605,606^ In CAD patients’ monocytes and macrophages, excessive glucose metabolism enhances IL-6 and IL-1β production through mitochondrial ROS and nuclear PKM2, driving systemic inflammation. Targeting glycolysis, superoxide, and PKM2 tetramerization could potentially correct this pro-inflammatory phenotype.^607^ Macrophages from CAD patients are prolific producers of T cell chemo attractants (CXCL9, CXCL10), pro-inflammatory cytokines (IL-1β, IL-6), and the immunosuppressive ligand PD-L1, highlighting their significant role in exacerbating the inflammatory environment.^608^ NPM1 recruits histone demethylase KDM5b to the Tsc1 promoter, erasing H3K4me3 marks, inhibiting TSC1 expression, and enhancing mTOR signaling. Deficiency of NPM1 in macrophages mitigates myocardial ischemic injury, improves cardiac function, and promotes tissue repair after myocardial infarction by shifting cardiac macrophages towards oxidative phosphorylation metabolism and a reparative phenotype^543^ (Fig. 8d).

#### Cell death-related pathway

Cell death-related pathways regulate programmed cell death mechanisms such as apoptosis, necroptosis, and autophagy. Apoptosis involves caspase activation triggered by intrinsic (mitochondrial) or extrinsic (death receptor) pathways, leading to DNA fragmentation and cell dismantling. Necroptosis, a form of regulated necrosis, is mediated by RIPK1, RIPK3, and MLKL, causing cell membrane rupture. Autophagy involves the formation of autophagosomes that enclose cellular components, which are subsequently degraded by lysosomes, playing a crucial role in regulating the immune system^609^ (Fig. 8e).

The roles of immunogenic cell death in cardiac disease have yet to be fully defined, and biology-based strategies to inhibit cell death in various cardiac syndromes are also explored.^610^ In macrophages, ER stress activates the UPR, leading to apoptosis, with the PI3K/Akt pathway providing anti-apoptotic protection; JNK1 opposes Akt signaling, affecting atherosclerosis progression and plaque stability.^611^ CD47, an anti-phagocytic molecule that makes cells resistant to efferocytosis, is associated with atherosclerosis. CD47-blocking antibodies reverse this defect, normalize the clearance of diseased vascular tissue, and ameliorate atherosclerosis.^612^ Efferocytosis-derived nucleotides activate a DNA-PKcs-mTORC2/Rictor pathway, promoting non-inflammatory macrophage proliferation, apoptotic cell clearance, and tissue resolution, aiding in atherosclerosis regression and plaque stabilization.^613^ Myeloid CD147 promotes atherosclerosis by enhancing inflammation via the TRAF6-IKK-IRF5 pathway and inhibiting efferocytosis by suppressing GAS6. Anti-human CD147 antibodies reduce atherosclerosis, suggesting a new therapeutic approach.^614^ Serum immunity-related GTPase family M protein (IRGM) is linked to plaque rupture in STEMI patients. IRGM/Irgm1 deficiency increases plaque stability and suppresses macrophage apoptosis by inhibiting ROS production and MAPK signaling.^615^ Solute Carrier Family 26 Member 4 (SLC26A4), identified as a potential asthma target,^616^ also contributes to cardiac hypertrophy by promoting autophagy and inducing apoptosis in cardiomyocytes.^617^ In an in vitro starvation model, neonatal mouse cardiomyocytes from WT mice and those with macrophage migration inhibitory factor (MIF) depletion showed a significant reduction in starvation-induced autophagic vacuole formation and an increase in starvation-induced cell death in H9C2 cells. These results suggest that MIF plays a supportive role in maintaining cardiac contractile function during starvation by regulating autophagy.^618,619^ SIRT1 levels are repressed, and acetylated p53 levels are enhanced in CAD patient monocytes, increasing pro-apoptotic events and pro-inflammatory responses, contributing to vessel damage and long-term recurrent ischemic events.^620^

By integrating multi-disciplinary efforts that include genomic, epigenetic, and immune response studies, we can identify novel biomarkers and therapeutic targets. This can facilitate the development of personalized treatments that modulate immune responses and key signaling pathways involved in CVD progression. Additionally, adopting flexible clinical trial designs, akin to those used in cancer research, will allow for rapid validation of new therapies and improved patient outcomes. Ultimately, these innovations promise to advance the clinical translation of cardiovascular research, offering new hope for better management of heart diseases.

The integration of key signaling pathways in CVDs highlights the complex interplay between immune responses and cardiovascular mechanisms. This intricate crosstalk, involving pathways such as GPCR, growth factor receptors, oxidative stress, and cell metabolism, underscores the importance of understanding how these processes influence immune regulation in CVDs. By investigating these connections through multidisciplinary efforts—including genomic, epigenetic, and immune response studies—researchers can uncover novel biomarkers and therapeutic targets that facilitate the development of personalized treatments. Such approaches aim to modulate immune responses and regulate key signaling pathways, enhancing cardiac repair, reducing inflammation, and ultimately improving patient outcomes. Adopting flexible clinical trial designs, similar to those used in oncology, will further accelerate the validation of new therapies and support the clinical translation of cardiovascular research, offering renewed hope for advancing CVDs management.

## Immune-based therapeutic strategies for targeting CVDs

Immune-based therapeutic strategies for targeting CVDs represent a growing field aimed at modulating the immune system to prevent and treat these conditions. This approach categorizes potential therapeutic agents into three main groups: biologics, gene and molecular therapies, and chemical Drugs, based on existing preclinical studies. Given the current landscape, there is a pressing need to identify new targets and develop innovative treatment strategies for effective cardiovascular disease management (Table 1).

**Table 1:** Selected published articles on immune-regulating therapeutic agents in preclinical development

Drugs	Indication	Therapeutic agents	Targets
HCW-9302^622^	Atherosclerosis	Peptide-fusion proteins	IL-2R Factor VIII
Anti-CD45 antibody-drug conjugate^628^	Atherosclerosis	Antibody-drug conjugates	CD45
APTA-5278^635,636^	Atherosclerosis	Chemical drugs	NOX
AZ-6983^637^	Atherosclerosis	Small molecule drugs	α7nAChR
VSB-16^625^	Atherosclerosis	Monoclonal antibody	CCL4L1
CD40L/Mac-1 interaction inhibitors^627^	Atherosclerosis	Antibody peptide	CD11b CD40LG ITGB2
BRP-187^638^	Atherosclerosis	Chemical drugs	LOX5 FLAP
Endothelial lipase inhibitors^639^	Atherosclerosis	Chemical drugs	LIPG
NLRP3 inflammasome inhibitor^647^	Myocardial infarction	Chemical drugs	NLRP3
NH2-terminally truncated galectin-3^632,634^	Myocardial infarction	Recombinant peptide	LGALS3
SIM-339^639^	Cerebral hemorrhage Myocardial infarction	Peptide-coupled drug	JNK
RBB-004^624^	Myocardial infarction	Antibody-fusion proteins	HSP70
TNAX-103A^626^	Myocardial infarction	Humanized monoclonal antibodies	CD300A
NWL-283^631^	Myocardial infarction	Peptidomimetic drug	CASP3
APD-588^643,644^	Heart failure	Chemical drugs	S1PR
SR-9009^645^	Myocardial infarction Heart failure	Chemical drugs	REV-ERB
SA-12^641^	Heart failure	Chemical drugs	MPO
GDF15 modulator^646^	Congestive heart failure	Chemical drugs	GDF15
CAR-T cells^45^	Cardiac fibrosis	Adoptive T cell transfer	FAP
In vivo-generated CAR T cells^15^	Cardiac fibrosis	LNP-encapsulated modified mRNA	FAP

*IL-2R* Interleukin-2 Receptor, *CD45* Cluster of Differentiation 45, *NOX* NADPH Oxidase, α7nAChR Alpha-7 Nicotinic Acetylcholine Receptor, *MPO* Myeloperoxidase, *CCL4L1* C-C Motif Chemokine Ligand 4-Like 1, *CD11b* Cluster of Differentiation 11b, *CD40LG* CD40 Ligand, *ITGB2* Integrin Beta-2, *LOX5* 5-Lipoxygenase, *FLAP* 5-Lipoxygenase Activating Protein, *LIPG* Endothelial Lipase (LIPG), *HDAC6* Histone Deacetylase 6, *NLRP3* NOD-like Receptor Pyrin Domain Containing 3, *LGALS3* Galectin-3, *JNK* c-Jun N-terminal Kinase, *HSP70* Heat Shock Protein 70, *CD300A* Cluster of Differentiation 300a, *CASP3* Caspase-3, *S1PR* Sphingosine-1-Phosphate Receptor, *REV-ERB* REV-ERB Nuclear Receptors (including REV-ERBα and REV-ERBβ), *GDF15* Growth Differentiation Factor 15, *FAP* Fibroblast Activation Protein

### Biologics

Biologics, as defined by the FDA, are products made of sugars, proteins, nucleic acids, or their combinations, including live cells and tissues, and encompass vaccines, blood components, allergenic drugs, somatic cells, gene therapies, tissues, and recombinant proteins.^621^ Relevant biologics for CVDs treatment include key subcategories such as fusion proteins, monoclonal antibodies, antibody peptides, CAR-T cell therapy, and peptidomimetic. Notably, fusion proteins like HCW-9302 and peptide-fusion proteins such as RBB-004 have shown promise in preclinical models. HCW-9302 targets IL-2R to promote the expansion of regulatory T cells for treating atherosclerosis in mice.^622^ RBB-004 targets HSP70 in myocardial infarction, where extracellular HSP70 enhances the innate immune response as an immunomodulator.^623,624^ Also, monoclonal antibodies and humanized monoclonal antibodies are essential for modulating immune responses in CVDs. VSB-16 targets CCL4L1 to stabilize atherosclerotic plaques and reduce inflammation by inhibiting metalloproteinases, decreasing pro-inflammatory cytokine production, and suppressing the NF-κB signaling pathway.^625^ TNAX-103A targets CD300A in myocardial infarction, enhancing efferocytosis by infiltrating myeloid cells.^626^ Antibody peptides and antibody-drug conjugates further broaden the range of therapeutic options. CD40L/Mac-1 interaction inhibitors prevent the recruitment of inflammatory leukocytes and reduce inflammation in atherosclerosis by targeting CD11b, CD40LG, and integrin subunit beta 2 (ITGB2).^625,627^ The novel anti-CD45 ADC, initially used for anti-tumor therapy, is now being used to treat atherosclerosis by reducing the atherosclerotic plaque burden.^628^ Moreover, CAR-T cell therapy is currently under development. Antigen-specific CD8 T cells are used for adoptive transfer to effectively target and ablate cardiac fibroblast proteins, thereby reducing cardiac fibrosis.^45^ Additionally, peptidomimetics and peptide-conjugated drugs such as NWL-283 and SIM-339 target Caspase3 and c-Jun NH2-terminal Kinase (JNK), respectively, to reduce apoptosis and inflammatory responses in myocardial infarction and cerebral hemorrhage.^629–631^ Galectin-3 (Gal-3) inhibitors, which target the protein involved in collagen synthesis, macrophage infiltration, and interstitial fibrosis, are being explored to treat myocardial infarction.^632–634^

### Gene and molecular therapies

Gene and molecular therapies involve the manipulation of genetic material to treat diseases. This category includes viral gene delivery systems and LNP-encapsulated modified mRNA. A notable approach includes generating antifibrotic CAR T cells in vivo by delivering modified mRNA via T cell-targeted LNPs. These cells have shown promise in mice, effectively reducing fibrosis and restoring cardiac function.^15^

### Chemical drugs

Chemical drugs encompass a wide range of compounds with diverse mechanisms of action. For atherosclerosis, several targets have been identified to reduce inflammatory responses and arterial plaque formation. APTA-5278, an inhibitor targeting NADPH oxidase-dependent ROS formation, reduces oxidative stress and inflammation.^635,636^ AZ6983 activates α7nAChR to inhibit atherosclerosis and enhance phagocytosis in myeloid cells.^637^ BRP-187 is a leukotriene synthesis inhibitor that prevents the assembly of the FLAP complex, reducing the production of pro-inflammatory leukotrienes.^638^ Endothelial lipase inhibitors target LIPG to reduce pro-inflammatory effects and improve high-density lipoprotein levels.^639,640^ For heart failure, myeloperoxidase (MPO) inhibitors such as SA-12 significantly improve cardiac function and inhibit myocardial structural changes in non-ischemic heart failure mouse models.^641^ APD588, a selective S1P receptor modulator, regulates inflammatory responses and improves cardiac functional recovery following myocardial infarction.^642–644^ SR9009, a synthetic compound activating REV-ERB receptors, aids in long-term cardiac repair following myocardial ischemia-reperfusion.^645^ GDF-15 modulators are used to treat congestive heart failure by mitigating inflammation and tissue damage.^646^ In myocardial infarction, early inhibition of NLRP3 activation can reduce infarct size and protect cardiac function, making inflammasome inhibitors a promising treatment for acute myocardial infarction.^647^ Additionally, DNMT inhibitors and other epigenetic therapies might help modulate macrophage functions to prevent or treat CVDs. Indeed, inhibition of DNMT3b has been found to increase the expression of Tregs while decreasing the levels of pro-inflammatory cytokines such as IL-1β and IFN-γ, thereby regulating the inflammatory response and the development of atherosclerosis.^648–650^ In heart transplantation, FNVs@RAPA utilize a ROS-responsive bio-orthogonal chemistry approach for active targeting delivery to the heart graft site, effectively alleviating IRI and promoting the polarization of Ly6C + Ly6G-inflammatory macrophages towards an anti-inflammatory phenotype.^651^ The MNPs/Alg hydrogel, composed of melanin nanoparticles and alginate, eliminates ROS, promotes macrophage polarization to regenerative M2 macrophages, and provides antioxidant, anti-inflammatory, and proangiogenesis effects, showing great potential for myocardial infarction treatment and cardiac repair.^652^

## Clinical trials

Current clinical trials on immunomodulation in CVDs encompass broad-spectrum immunosuppressants, target-inflammatory treatments, cell therapies, and novel immunomodulatory targets, primarily targeting atherosclerosis and acute myocardial infarction (Table 2).

**Table 2:** Clinical trials with immunomodulatory therapeutics for cardiovascular diseases

Study names	Drugs	Mechanism of action	Targets	Phase	Patient cohort	Primary endpoints	Main outcomes	NCT number
Lodoco2 (2020)^690^	Colchicine	Microtubule inhibitor	TUBB	Phase3	Patients with CAD	MACE	Low-dose colchicine significantly reduced cardiovascular events.	ACTRN12614000093684
COLCOT (2019)^47^	Colchicine	Microtubule inhibitor	TUBB	Phase3	Patients with MI	MACE	0.5 mg daily colchicine significantly reduced ischemic cardiovascular risk	NCT02551094
NA	Colchicine	Microtubule inhibitor	TUBB	Phase 2	Patients with HFpEF	Change in hs-CRP	NA	NCT04857931
NA	Colchicine	Microtubule inhibitor	TUBB	NA	Patients with AAA	Changes in maximum diameter of AAA	NA	NCT05361772
NA	Colchicine	Microtubule inhibitor	TUBB	Phase 3	Patients with CHD requiring PCI	MACE	NA	NCT06472908
COCS (2024)	Colchicine	Microtubule inhibitor	TUBB	Phase4	Patients awaiting elective cardiac surgery (CABG/AVR)	Postoperative atrial fibrillation incidence	NA	NCT04224545
CIRT (2018)^653^	Methotrexate	Broad immuno—suppression dihydrofolate reductase inhibitor	A2AR	Phase3	Patients with prior MI or multivessel CAD by angiography	MACE	Methotrexate didn’t reduce IL-1β, IL-6, CRP, or cardiovascular events	NCT01594333
NA	Methotrexate	Broad immuno—suppression dihydrofolate reductase inhibitor	A2AR	Phase4	Patients with RA	Change in peripheral SBP	NA	NCT03254589
CAPRI (2020)^654^	Ciclosporin	Broad immuno—suppression Calcineurin inhibitors	MPTP	Phase2	Patients with STEMI and undergoing PCI	Change in infarct size	Single ciclosporin bolus had no effect on infarct size or LV remodeling	NCT02390674
Mohd Ali et al. (2018)^655^	Sirolimus	Broad immuno—suppression mTOR inhibitors	FKBP 12	Phase1	Patients with coronary DES restenosis	Late lumen loss	Novel SCB vs. proven PCB for coronary DES ISR shows similar angiographic outcomes	NCT02996318
ORAR^656^	Rapamycin	mTOR inhibitor	mTOR	Phase3	Patients with BMS implantation	Cost differences in revascularization for de novo lesions	No outcome difference between oral rapamycin + BMS and DES for de novo lesions	NCT00552669
CLEVER--ACS^657^	Everolimus	mTOR inhibitor	mTOR	Phase1/2	Patients with STEMI	MI size measured by MRI	Treatment didn’t reduce MI size or MVO at 30 days	NCT01529554
CANTOS (2017)^46^	Canakinumab	Anti--interleukin-1β antibodies	IL-1β	Phase 3	Patients with MI	MACE	150 mg canakinumab every 3 months significantly lowered recurrent cardiovascular events versus placebo	NCT01327846
VCUART3 (2020)^659^	Anakinra	IL-1 receptor antagonist	IL-1Ra	Phase 2	Patients with STEMI	The AUC for hsCRP	Compared to placebo, it significantly reduces the systemic inflammatory response	NCT01950299
MAGiC-ART(2020)	Anakinra	IL-1 receptor antagonist	IL-1Ra	Phase 2	Patients with cardiac sarcoidosis	Change in inflammation marker	NA	NCT04017936
Myachikova et al. ^660^	Goflikicept	IL-1 inhibitor	IL-1β	Phase 2/3	Patients with idiopathic recurrent pericarditis	Time to first pericarditis recurrence was evaluated	Goflikicept reduced recurrence risk vs. placebo	NCT04692766
Sayed et al.^658^	Xilonix	IL-1α inhibitor	IL-1α	Phase 2	Patients after PCI	Target vessel restenosis, time to restenosis, and MACE incidence	At 12 months, no significant difference in MACE or target vessel restenosis between groups	NCT01270945
RESCUE ^661^	Ziltivekimab	IL-6-targeting monoclonal antibody	IL-6	Phase 2	Patients with moderate to severe CKD	12-week change in hs-CRP	Ziltivekimab markedly reduced atherosclerosis--related inflammation and thrombosis biomarkers	NCT03926117
ARTEMIS (2024)	Ziltivekimab	IL-6-targeting monoclonal antibody	IL-6	Phase3	Patients with AMI	Time to first 3-component MACE	NA	NCT06118281
ATHENA (2024)	Ziltivekimab	IL-6-targeting monoclonal antibody	IL-6	Phase3	Patients with HF	Change in KCCQ-CSS	NA	NCT06200207
ASSAIL-MI (2021)^663^	Tocilizumab	IL-6-targeting monoclonal antibody	IL-6	Phase 2	Patients with STEMI within 6 h undergoing PCI	Myocardial salvage index (%)	Tocilizumab increased myocardial salvage in patients with acute STEMI	NCT03004703
Kleveland. et al. ^662^	Tocilizumab	IL-6-targeting monoclonal antibody	IL-6	Phase 2	Patients with NSTEMI	Between-group AUC difference for hs-CRP (days 1–3)	Tocilizumab attenuated the inflammatory response	NCT01491074
IMICA (2021)^664^	Tocilizumab	IL-6-targeting monoclonal antibody	IL-6	Phase2	Patients with out-of-hospital cardiac arrest	Reduction in CRP levels at 72 h	Tocilizumab reduced systemic inflammation and myocardial injury in comatose patients post-cardiac arrest.	NCT03863015
NA	NT-0796	NLRP3 inhibitor	NLRP3	Phase 2	Patients with BMI ≥ 30 and ≤40 kg/m^2^	Change in hsCRP levels	NA	NCT06129409
NA	DFV890	NLRP3 inhibitor	NLRP3	Phase 2	Patients with MI (ages 18–85, BMI 18–45 kg/m², hsCRP ≥ 2 mg/L)	Serum levels of IL-6 and IL-18	NA	NCT06031844
Wohlford et al. ^667^	Dapansutrile	Selective NLRP3 Inflamma--some Inhibitor	NLRP3	Phase 1b	Patients with stable systolic HF, LVEF ≤ 40%, NYHA II-III symptoms	AEs	14-day dapansutrile treatment was safe and well-tolerated in stable HFrEF patients	NCT03534297
CATCH-AMI (2013)	Balixafortide (POL6326)	CXCR4 antagonist	CXCR4	Phase IIa	Patients with reperfused STEMI	Change in LVEF determined by MRI	NA	NCT01905475
NA	Etanercept	TNF-α inhibitor	TNF-α	Phase4	Patients with AMI	MACE	NA	NCT01372930
Colombo et al. ^669^	Bindarit	Selective inhibitor of monocyte chemotactic protein-1 (MCP-1/CCL2)	MCP-1/CCL2	Phase IIa	Patients with coronary BMS	In-segment late loss	Bindarit significantly reduced in-stent late loss, indicating potential vessel wall benefits post-angioplasty	NCT01269242
NA	BRB-002	Novel Anti-CD47 Molecule	CD47	Phase 1	Healthy male volunteers	To evaluate the safety and tolerability of BRB-002	NA	ACTRN12624000405516
NA	Atibuclimab	Chimeric monoclonal antibody targeting CD14	CD14	Phase 1b	Patients with ACM	Safety and efficacy of the drug	NA	NCT06275893
Chen et al. ^675^	RTP-026	Annexin-A1 analog	FPR2	Phase2	Patients with STEMI undergoing PCI, chest pain <12 h, NLR 7-17	cTNT/CK-MB at 24 hours	NA	NCT06465303
Hernández-Jiménez et al. ^671^	ApTOLL	Toll-like receptor 4 antagonist	TLR4	Phase1	Health male volunteers	Assess safety and pharmacokinetics of 30-min IV ApTOLL infusion	No ApTOLL accumulation, confirming safety and supporting clinical trials	NCT04742062
SATELLITE (2023)^670^	AZD4831	Myelo--peroxidase inhibitor	Mpo	Phase2	Patients with HFpEF	Myeloperoxidase specific activity	AZD4831 was safe and effectively inhibited myeloperoxidase.	NCT03756285
RESTORE (2022)	OPL-0301	S1PR1 agonist	S1PR1	Phase 2	Patients with acute STEMI	Infarct size by CMR at Day 90	NA	NCT05327855
HUCV002-01 (2022)^684^	αGCDC	α-galactosylceramide-pulsed dendritic cells (αGCDCs)	INKT cell	Phase 2	Patients with CHF	Change in LVEF from baseline to 24 weeks	NA	jRCT2073210116
Hare et al. (2005)^680^	Adult hMSCs	Cell-based immuno--modulators	DMMI	Phase1	Patients with MI	AEs rates in 0.5, 1.6, and 5.0 million MSC/kg dose cohorts vs. placebo	Similar adverse event rates between hMSC and placebo groups	NCT00114452
Lee et al. ^681^	SEED-MSC (BM-MSCs)	Cell-based immuno--modulators	DMAMI	Phase2/3	Patients with AMI	Absolute changes in global LVEF by SPECT	Safe and tolerable, showing modest LVEF improvement at 6 months by SPECT	NCT01392105
Chullikana et al. ^685^	Stempeuce (BM-MSCs)	Cell-based immuno--modulators	DMAMI	Phase1/2	Patients with STEMI	AEs and ECG parameters	Safe and well-tolerated IV in AMI patients 2 days post-PCI	NCT00883727
Butler (2016)^686^	aMBMC	Cell-based immuno--modulators	NICM	Phase 2a	Patients with non-ischemic Heart Failure	Safety by number of AEs	Safe, immunomodulatory effects, with improved health status and functional capacity	NCT02467387
NA	BM-MSCs	Cell-based immuno--modulators	DMAMI	Phase3	Patients with AMI	Change in LVEF	NA	NCT01652209
TRIDENT (2017)^687^	hMSC	Cell-based immuno--modulators	ICM	Phase 2	Patients with ischemic cardiomyopathy	Number of Participants With TE-SAEs	100 million dose increased ejection fraction; both doses reduced scar size	NCT02013674
TAC-HFT-II (2020)	hmsc/hCSC	Cell-based immuno--modulators	DMMI	Phase1/2	Patients with chronic ischemic LV dysfunction and HF post-MI	Incidence of any TE-SAEs	NA	NCT02503280
WJ-MSC-AMI (2015)^682^	WJMSCs	Cell-based immuno--modulators	DMSTEMI	Phase2	Patients with AMI	Myocardium metabolic and perfusion measurements, global LVEF by echocardiography	Intracoronary WJMSCs safe and effective in AMI, clinically relevant therapy	NCT01291329
RIMECARD (2016)^688^	UC-MSC	Cell-based immuno--modulators	LV function in HFrEF	Phase1/2	Patients with compensated HF (dilated phase)	Change in global LVEF	IV UC-MSCs safe in stable HF with reduced LVEF	NCT01739777
HUC-HEART Trial ^689^	HUC-MSCs	Cell-based immuno--modulators	ICM	Phase1/2	Patients with chronic ischemic CM	Ventricular remodeling	Intramyocardial HUC-MSCs effective in CIC	NCT02323477
NA	UC-MSCs	Cell-based immuno--modulators	DMMI	Phase1	Patients with MI	MACE	NA	NCT03902067
NA	Clinical-grade WJ-MSCs	Cell-based immuno--modulators	DMSTEMI	Phase1/2	Patients with STEMI	MI size	NA	NCT03533153
Qayyum. et al. ^683^	ADSCS	Cell-based immuno--modulators	LV function in HFrEF	Phase2	Patients with HFrEF	Change in LVESV	Safe but no improvement in myocardial function or symptoms	NCT03092284

*CAD* Coronary Artery Disease, *MACE* Major Adverse Cardiovascular Events, *MI* Myocardial Infarction, *HFpEF* Heart Failure with Preserved Ejection Fraction, *hs-CRP* High-Sensitivity C-Reactive Protein, *AEs* Adverse event, *TE-SAEs* Treatment emergent serious adverse events, *AAA* Abdominal Aortic Aneurysm, *CHD* Coronary Heart Disease, *PCI* Percutaneous Coronary Intervention, CABG Coronary Artery Bypass Grafting, AVR Aortic Valve Replacement, *RA* Rheumatoid Arthritis, DES Drug-Eluting Stent, BMS Bare Metal Stent, *NSTEMI* Non-ST-Elevation Myocardial Infarction, *CKD* Chronic Kidney Disease, *KCCQ-CSS* Kansas City Cardiomyopathy Questionnaire Clinical Summary Score, AUC Area Under the Curve, *CRP* C-Reactive Protein, IL-6 Interleukin-6, IL-18 Interleukin-18, LVEF Left Ventricular Ejection Fraction, NLR Neutrophil-to-Lymphocyte Ratio, SPECT Single Photon Emission Computed Tomography, *CMR* Cardiac Magnetic Resonance, *cTNT* Cardiac Troponin T, *CK-MB* Creatine Kinase-MB, UC-MSC Umbilical Cord Mesenchymal Stem Cells, HUC-MSC Human Umbilical Cord Mesenchymal Stem Cells, ACM Arrhythmogenic Cardiomyopathy, CHF Congestive Heart Failure, LVESV Left Ventricular End-Systolic Volume, MVO Myocardial Viability Outcome, CM Cardiomyopathy, AMI Acute Myocardial Infarction, *AE* Adverse Event, STEMI ST-Elevation Myocardial Infarction, *Tubb* Tubulin Beta Chain, *A2AR* Adenosine *A2A* Receptor, *MPTP* Mitochondrial Permeability Transition Pore, *FKBP12* FK506 Binding Protein 12, *mTOR* Serine/Threonine-Protein Kinase mTOR, *FPR2* N-Formyl Peptide Receptor 2, *TLR4* Toll-Like Receptor 4, *DMMI* Damaged Myocardium in Myocardial Infarction, *DMAMI* Damaged Myocardium in Acute Myocardial Infarction, *NICM* Myocardium in Nonischemic Cardiomyopathy, *ICM* Myocardium in Ischemic Cardiomyopathy, *DMSTEMI* Damaged Myocardium in ST-Elevation Myocardial Infarction, *HFrEF* Heart Failure with Reduced Ejection Fraction

### Broad-spectrum immunosuppressants

The efficacy of broad-spectrum immunosuppressants like Ciclosporin, sirolimus, rapamycin, and everolimus in CVD has been limited. The CIRT study (NCT01594333) revealed that methotrexate did not reduce inflammation markers or major adverse cardiovascular events (MACE) in coronary artery disease patients.^653^ Ciclosporin (NCT02390674) did not significantly impact infarct size or left ventricular remodeling in acute myocardial infarction patients.^654^ Studies on sirolimus (NCT02996318) and rapamycin (NCT00552669) for drug-eluting stents (DES) to inhibit restenosis also reported negative outcomes.^655,656^ Everolimus (NCT01529554) failed to reduce myocardial infarction size in acute ST-elevation myocardial infarction patients.^657^ These findings suggest that while these immunosuppressants hold potential in other diseases, their effectiveness in CVDs, particularly in acute myocardial infarction and stent-related treatments, is limited.

### Target-inflammatory treatments

IL-1 and IL-6 are central to the inflammatory response, with varying outcomes in related studies. El Sayed et al. found no significant differences in MACE and target vessel restenosis rates between groups using the IL-1α inhibitor Xilonix after percutaneous coronary intervention.^658^ The CANTOS study (NCT01327846) on 10,061 post-myocardial infarction patients showed that quarterly administration of 150 mg canakinumab significantly reduced recurrent cardiovascular events.^46^ The VCUART3 study demonstrated that anakinra (an IL-1 receptor antagonist) significantly reduced systemic inflammation in 99 ST-elevation myocardial infarction patients.^659^ Studies have shown that IL-1α and IL-1β are key cytokines in the pathophysiology of acute pericarditis and its recurrence, with rilonacept preventing recurrences and maintaining remission in idiopathic recurrent pericarditis (IRP).^660^

IL-6, as a downstream inflammatory marker of IL-1, has also been a focus of research. The RESCUE study (NCT02660034) showed that ziltivekimab, an IL-6 targeting monoclonal antibody, significantly reduced atherosclerotic inflammation markers like high-sensitivity CRP and thrombosis markers in patients with moderate to severe chronic kidney disease.^661^ Kleveland et al. (2016) found that tocilizumab, an IL-6 monoclonal antibody, reduced inflammation in patients with acute non-ST-elevation myocardial infarction.^662^ The ASSAIL-MI study (NCT03004703) indicated that tocilizumab improved the myocardial salvage index in acute ST-elevation myocardial infarction patients undergoing PCI.^663^ The IMICA study (NCT03640180) demonstrated that tocilizumab significantly reduced systemic inflammation and myocardial injury in out-of-hospital cardiac arrest patients.^664^

The NLRP3 inflammasome, an upstream activator of IL-1β and IL-18, plays a crucial role in the strong inflammatory response during myocardial ischemic and non-ischemic injury.^329^ The non-selective NLRP3 inflammasome inhibitor colchicine has been shown to reduce cardiovascular events in coronary artery disease patients over the long term in the Lodoco2 (NCT02285360) and COLCOT (NCT02551094) trials, leading to FDA approval for cardiovascular anti-inflammatory treatment.^665^ Selective NLRP3 inflammasome inhibitors like dapansutrile have shown good safety and tolerability in HFrEF patients, warranting further research on their efficacy.^666,667^ NT0796 and DFV890, also selective NLRP3 inflammasome inhibitors, are currently in phase 2 clinical trials for coronary artery disease patients (NCT06129409, NCT06031844).

In addition to classical targeted therapies for inflammation, several non-classical inflammatory-targeted treatments have also emerged. Although, some clinical trials have yet to achieve their primary endpoints or disclose conclusive results. For instance, etanercept (NCT01372930), a TNF-α inhibitor, reduces inflammation by blocking TNF-α produced by macrophages,^668^ though clinical trial results are yet to be revealed. Bindarit (NCT01269242), selectively inhibiting monocyte chemoattractant protein-1 (MCP-1/CCL2), reduces monocyte chemotaxis and infiltration, significantly lowering late lumen loss in coronary bare-metal stent patients, despite not meeting primary endpoints.^669^ AZD4831 (NCT03756285), a myeloperoxidase (MPO) inhibitor, reduces inflammation and improves microvascular function, showing good tolerability in heart failure patients.^670^ ApTOLL (NCT04742062), a TLR4 antagonist, reduces inflammation following acute ischemic stroke and acute myocardial infarction by blocking TLR4 signaling, with good safety and pharmacokinetic profiles.^671^

### Novel immunomodulatory targets

There also strategies focused on precisely modulating immune responses involved in tissue repair, immune cell migration, and receptor signaling. Notably, BRB-002 (ACTRN12624000405516) enhances macrophage phagocytosis and modulates atherosclerotic immune responses by blocking CD47-SIRPα interaction.^672^ RTP-026 (NCT06465303), an Annexin-A1 analog, reduces myocardial injury by regulating immune cell migration and reactivity in acute and chronic cardiovascular disease models.^673–675^ Atibuclimab (NCT06275893) targets CD14, regulating inflammation, apoptosis, and tissue injury responses, with ongoing evaluations of its safety and efficacy.^676^ POL6326 (NCT01905475), a CXCR4 antagonist, mediates angiogenesis and tissue repair through splenic Foxp3 regulatory T cells, improving cardiac function post-myocardial infarction.^677^ S1P receptors regulate cardiac fibroblast remodeling, proliferation, and differentiation, mediating peripheral vascular tone and endothelial responses.^678^ An ongoing clinical trial (NCT05327855) is held to evaluate the efficacy of OPL-0301, a S1PR1 agonist, for myocardial injury in acute myocardial infarction patients.

### Cell therapy

It is widely acknowledged that Mesenchymal stem cells (MSCs) are highly immunomodulatory. MSCs have demonstrated promising potential in myocardial protection, mainly through their abilities to reduce inflammation, promote cardiomyocyte differentiation, enhance angiogenesis, increase anti-apoptotic capacity, and inhibit fibrosis.^679^

The first clinical trial involving adult MSCs in acute myocardial infarction patients, conducted by Joshua Hare et al. (2005), confirmed their safety.^680^Since then, MSCs derived from various sources, such as bone marrow, umbilical cord, and adipose tissue, have been shown to be safe and well-tolerated in clinical trials for acute myocardial infarction and heart failure patients.^681–683^In addition to MSC-based therapies, other immunomodulatory cell therapies, such as α-galactosylceramide-pulsed dendritic cells (αGCDC), have shown potential for chronic heart failure, with research still ongoing (jRCT2073210116).^684^

Several recent studies have focused on MSCs specifically for cardiovascular applications. In a Phase 1/2 trial (NCT00883727), Chullikana et al. demonstrated that intravenous administration of Stempeuce (BM-MSCs) was safe and well-tolerated in STEMI patients, with no significant adverse effects reported 2 days post-PCI.^685^ Similarly, Butler et al. conducted a Phase 2a trial (NCT02467387) using autologous bone marrow MSCs (aMBMC) in non-ischemic heart failure patients, showing that the treatment was not only safe but also had potential immunomodulatory benefits, with improved health status and functional capacity.^686^ Furthermore, a Phase 3 trial (NCT01652209) using BM-MSCs in AMI patients assessed changes in left ventricular ejection fraction (LVEF), although detailed efficacy results were not disclosed. The TRIDENT study (2017) (NCT02013674) extended these findings by demonstrating that a high dose of hMSCs improved ejection fraction and reduced scar size in patients with ischemic cardiomyopathy.^687^ Similarly, the TAC-HFT-II trial (2020) (NCT02503280) investigated a combination of hMSC/hCSC in patients with chronic ischemic LV dysfunction and heart failure post-MI, focusing on treatment-emergent serious adverse events.

Research on MSCs from other sources, such as umbilical cord MSCs (UC-MSCs), has also progressed. The RIMECARD study (2016) (NCT01739777) found that intravenous UC-MSCs were safe in stable heart failure patients with reduced LVEF.^688^ Additionally, the HUC-HEART Trial (2020) (NCT02323477) demonstrated that intramyocardial HUC-MSCs effectively promoted ventricular remodeling in patients with chronic ischemic cardiomyopathy.^689^ Further studies included a Phase 1 trial (NCT0390206) investigating UC-MSCs in MI patients, with MACE as the primary outcome, and a separate trial (NCT03533153) examining clinical-grade WJ-MSCs in STEMI patients, with a focus on reducing myocardial infarct size.

In brief, these studies highlight the safety and therapeutic potential of MSC-based therapies for improving cardiac function and remodeling across various cardiovascular conditions. However, to confirm these benefits and optimize the most effective MSC sources and delivery methods, further large-scale studies are essential.

### Future strategies for immunomodulation in CVDs

In contemporary clinical research addressing CVDs, these conditions are increasingly recognized as being intricately linked to immune responses.Early clinical investigations utilized traditional broad-spectrum immunosuppressive agents, such as methotrexate, cyclosporine, sirolimus, rapamycin, and everolimus, to evaluate their efficacy in the treatment of cardiovascular diseases.^681^ However, these studies did not yield significant reductions in the incidence of MACE, nor did they effectively decrease inflammatory markers or ameliorate myocardial damage following acute myocardial infarction.

The emergence of cell therapies, particularly MSCs treatments, has offered new avenues for managing cardiovascular disease. MSCs have demonstrated potential in modulating immune mechanisms, downregulating immune cell activity, and mitigating inflammatory responses. Despite the promising safety and tolerability observed in clinical trials involving MSCs derived from bone marrow, umbilical cord, and adipose tissue in patients with acute myocardial infarction and heart failure,^681–683^ the long-term benefits of these therapies for cardiovascular patients remain to be fully elucidated.

While the disappointing outcomes associated with broad-spectrum immunosuppressive drugs have prompted a shift in focus, targeted anti-inflammatory medications are gaining prominence. Notably, drugs aimed at IL-1, IL-6, and NLRP3 are under active investigation.^658–663^ Colchicine, a microtubule inhibitor and non-selective NLRP3 inhibitor, has demonstrated significant efficacy in reducing the incidence of MACE in patients with coronary artery disease and myocardial infarction, as evidenced by the results of two large-scale clinical trials, Lodoco2 and COLCOT.^47,690^ Following these findings, the FDA approved colchicine as an anti-inflammatory treatment for cardiovascular disease, marking a significant advancement in targeted anti-inflammatory strategies.

Moreover, clinical research targeting various immune modulation pathways is continuously evolving, with ongoing studies exploring novel targets such as CXCR4, MCP-1/CCL2,^669^ CD14, and CD47. Currently, targeted anti-inflammatory therapy represents a crucial strategy in the immunotherapeutic approach to cardiovascular diseases; however, the number of confirmed effective targets in clinical research remains relatively limited. In the pursuit of targeted anti-inflammatory therapies, it is essential to avoid pro-inflammatory responses that may lead to detrimental effects on the body, underscoring the need to maintain a delicate balance between anti-inflammatory and immunosuppressive mechanisms. Furthermore, the development of effective targeted drug delivery systems aimed at the heart will be a vital direction for future research, enabling more precise anti-inflammatory interventions in the treatment of cardiovascular diseases.

## Conclusion and perspective

This article provides a comprehensive overview of the research history and key milestones in the immunology and immunotherapy of CVDs. It offers a broad perspective on the activation and regulation of the immune response and details the multi-level regulatory signaling pathways and their crosstalk in CVDs. We also described the mechanisms of immune regulation in physiological and pathological processes of CVDs, as well as the advancements in targeted immune response therapy both in preclinical strategies and clinical trials.

Although the immune system plays a crucial role in CVDs, the complexity and variability of inflammatory processes across patients make it difficult to develop universally effective immune-targeted strategies. Several areas with significant gaps in mechanistic research need to be further explored: 1) There is a huge shortfall in characterizing cell-specific mechanistic pathways in CVDs. Meanwhile, understanding the complex network of interactions among various cell types is crucial for regulating CVDs. Utilize state-of-the-art techniques such as advanced in vivo imaging, genome-wide association studies, transgenic lineage tracing mice, making it possible to gain a deeper understanding of the immune landscape in CVDs; 2) The human body is an intricately regulated system of multiple interconnected systems. Therefore, it’s of great importance to explore the circuit among cardiovascular system-immune system-other system; 3) Given the dynamic changes in immune cell populations during CVDs, providing comprehensive information on these shifts, including multi-level regulation and the roles of anti-inflammatory and pro-inflammatory responses, is crucial for developing personalized treatments tailored to different disease stages; 4) Incomplete understanding of sex-specific CVDs mechanisms, especially conditions unique to women, underscores the need for targeted research. Moreover, large-scale studies should be designed separately for men and women to address these distinct pathophysiologies and develop gender-focused research systems; 5) Since morphological and molecular changes in diseases often occur asynchronously, disease phenotyping should incorporate both morphological and molecular classifications. Integrating these classifications is crucial for a comprehensive understanding of CVDs and for advancing clinical applications. Overall, these gaps will be resolved by the continuous development of single-cell technologies and computational analysis, coupled with powerful artificial intelligence-based histology, allowing for mapping disease-specific immune profiles.

From the perspective of cardiovascular immuno-modulatory therapies, the promising research directions are listed as follows: 1) Personalized inflammatory therapies are urgently needed for CVD treatment. Future research should focus on cell-specific mechanisms, intercellular interactions, dynamic immune changes across disease stages, and gender-specific immune regulations to translate immunological advances into clinical precision medicine; 2) Combination therapies targeting multiple immune pathways or combining immunotherapy with conventional treatments show promise in CVDs management. Moreover, considering holistic medicine, immune therapies targeted at other systems may benefit CVDs, making it possible to apply conventional medicine into new use. 3) While current research often emphasizes anti-inflammatory strategies, activating immune repair programs, such as stimulating Treg cells, could complement these therapies and help address their limitations in regulating CVDs; 4) Development of novel biomarker panels is essential to enhance cardiovascular risk prediction and guide treatment decisions. Machine learning and Mendelian randomization can enhance risk stratification and treatment selection using large-scale inflammatory biomarker data.

Overall, the ongoing development of basic and translational researches on immunotherapy in cardiovascular medicine may pave the way for more targeted and efficacious treatments, potentially reducing the burden of CVDs on global health. However, it is imperative to approach this promising field with caution, ensuring that rigorous scientific standards are maintained and patient safety remains paramount. As our understanding of the complex interplay between the immune system and cardiovascular health deepens, we may witness a paradigm shift in how CVDs are treated and managed in the future.

## References

[1] Roth, G. A. et al. Global burden of cardiovascular diseases and risk factors, 1990-2019: update from the GBD 2019 study. *J. Am. Coll. Cardiol.* **76**, 2982–3021 (2020). 33309175 10.1016/j.jacc.2020.11.010 PMC7755038

[2] Benjamin, E. J. et al. Heart disease and stroke statistics-2018 update: a report from the American Heart Association. *Circulation* **137**, e67–e492 (2018). 29386200 10.1161/CIR.0000000000000558

[3] Kazi, D. S. et al. Forecasting the economic burden of cardiovascular disease and stroke in the United States through 2050: a presidential advisory from the American Heart Association. *Circulation* **150**, e89–e101 (2024). 38832515 10.1161/CIR.0000000000001258

[4] Mann, D. L. The emerging role of innate immunity in the heart and vascular system: for whom the cell tolls. *Circ. Res.* **108**, 1133–1145 (2011). 21527743 10.1161/CIRCRESAHA.110.226936 PMC3084988

[5] Hansson, G. K. The heart of immunology: immune mechanisms in cardiovascular medicine. *Cardiovasc. Res.* **117**, e166–e168 (2021). 34849629 10.1093/cvr/cvab314

[6] Roy, P., Orecchioni, M. & Ley, K. How the immune system shapes atherosclerosis: roles of innate and adaptive immunity. *Nat. Rev. Immunol.* **22**, 251–265 (2022). 34389841 10.1038/s41577-021-00584-1 PMC10111155

[7] Bartoli-Leonard, F., Zimmer, J. & Aikawa, E. Innate and adaptive immunity: the understudied driving force of heart valve disease. *Cardiovasc. Res.* **117**, e166–e168 (2021). 34432007 10.1093/cvr/cvab273 PMC8783388

[8] Chen, R. et al. Macrophages in cardiovascular diseases: molecular mechanisms and therapeutic targets. *Signal. Transduct. Target Ther.* **9**, 130 (2024). 38816371 10.1038/s41392-024-01840-1 PMC11139930

[9] Varricchi, G., Marone, G. & Kovanen, P. T. Cardiac mast cells: underappreciated immune cells in cardiovascular homeostasis and disease. *Trends Immunol.* **41**, 734–746 (2020). 32605802 10.1016/j.it.2020.06.006

[10] Fernandez, D. M. et al. Single-cell immune landscape of human atherosclerotic plaques. *Nat. Med.* **25**, 1576–1588 (2019). 31591603 10.1038/s41591-019-0590-4 PMC7318784

[11] Chen, L. et al. Genetic drivers of epigenetic and transcriptional variation in human immune cells. *cell* **167**, 1398–1414 (2016). 27863251 10.1016/j.cell.2016.10.026 PMC5119954

[12] Martini, E. et al. Single-cell sequencing of mouse heart immune infiltrate in pressure overload-driven heart failure reveals extent of immune activation. *Circulation* **140**, 2089–2107 (2019). 31661975 10.1161/CIRCULATIONAHA.119.041694

[13] Abendstein, L., et al. Complement is activated by elevated IgG3 hexameric platforms and deposits C4b onto distinct antibody domains. *Nat. Commun.* **14**, 4027 (2023). 37419978 10.1038/s41467-023-39788-5 PMC10328927

[14] Rurik, J. G., Aghajanian, H. & Epstein, J. A. Immune cells and immunotherapy for cardiac injury and repair. *Circ. Res.* **128**, 1766–1779 (2021). 34043424 10.1161/CIRCRESAHA.121.318005 PMC8171813

[15] Rurik, J. G. et al. CAR T cells produced in vivo to treat cardiac injury. *Science* **375**, 91–96 (2022). 34990237 10.1126/science.abm0594 PMC9983611

[16] Lüscher, T. F. The sooner, the better: anti-inflammation in acute myocardial infarction. *Eur. Heart J.* **41**, 4100–4102 (2020). 33249465 10.1093/eurheartj/ehaa752

[17] Stamler, J. & Katz, L. N. Production of experimental cholesterol-induced atherosclerosis in chicks with minimal hypercholesterolemia and organ lipidosis. *Circulation* **2**, 705–713 (1950). 14783822 10.1161/01.cir.2.5.705

[18] Aqel, N. M., Ball, R. Y., Waldmann, H. & Mitchinson, M. J. Identification of macrophages and smooth muscle cells in human atherosclerosis using monoclonal antibodies. *J. Pathol.* **146**, 197–204 (1985). 3897495 10.1002/path.1711460306

[19] Jonasson, L. et al. Expression of class II transplantation antigen on vascular smooth muscle cells in human atherosclerosis. *J. Clin. Investig.* **76**, 125–131 (1985). 3894417 10.1172/JCI111934 PMC423725

[20] Jonasson, L. et al. Regional accumulations of T cells, macrophages, and smooth muscle cells in the human atherosclerotic plaque. *Arteriosclerosis* **6**, 131–138 (1986). 2937395 10.1161/01.atv.6.2.131

[21] Emeson, E. E. & Robertson, A. L. Jr. T lymphocytes in aortic and coronary intimas. Their potential role in atherogenesis. *Am. J. Pathol.* **130**, 369–376 (1988). 3257650 PMC1880529

[22] Hansson, G. K., Holm, J. & Jonasson, L. Detection of activated T lymphocytes in the human atherosclerotic plaque. *Am. J. Pathol.* **135**, 169–175 (1989). 2505620 PMC1880219

[23] Wedler, F. C., Hoffmann, F. M., Kenney, R. & Carfi, J. Maintainance of specificity, information, and thermostability in thermophilic Bacillus sp. glutamine synthetase. *Experientia Suppl.* **26**, 187–197 (1976). 7467 10.1007/978-3-0348-7675-9_15

[24] Nallamothu, B. K. et al. Relation between hospital specialization with primary percutaneous coronary intervention and clinical outcomes in ST-segment elevation myocardial infarction: National Registry of Myocardial Infarction-4 analysis. *Circulation* **113**, 222–229 (2006). 16401769 10.1161/CIRCULATIONAHA.105.578195

[25] Yellon, D. M. & Hausenloy, D. J. Myocardial reperfusion injury. *N. Engl. J. Med.* **357**, 1121–1135 (2007). 17855673 10.1056/NEJMra071667

[26] Fantone, J. C. & Ward, P. A. Role of oxygen-derived free radicals and metabolites in leukocyte-dependent inflammatory reactions. *Am. J. Pathol.* **107**, 395–418 (1982). 6282132 PMC1916241

[27] Babior, B. M., Kipnes, R. S. & Curnutte, J. T. Biological defense mechanisms. The production by leukocytes of superoxide, a potential bactericidal agent. *J. Clin. Investig.* **52**, 741–744 (1973). 4346473 10.1172/JCI107236 PMC302313

[28] Meerson, F. Z. et al. The role of lipid peroxidation in pathogenesis of ischemic damage and the antioxidant protection of the heart. *Basic Res. Cardiol.* **77**, 465–485 (1982). 7181828 10.1007/BF01907940

[29] Dick, S. A. & Epelman, S. Chronic heart failure and inflammation: what do we really know?. *Circ. Res.* **119**, 159–176 (2016). 27340274 10.1161/CIRCRESAHA.116.308030

[30] Levine, B. et al. Elevated circulating levels of tumor necrosis factor in severe chronic heart failure. *N. Engl. J. Med.* **323**, 236–241 (1990). 2195340 10.1056/NEJM199007263230405

[31] Ferrari, R. et al. Tumor necrosis factor soluble receptors in patients with various degrees of congestive heart failure. *Circulation* **92**, 1479–1486 (1995). 7664430 10.1161/01.cir.92.6.1479

[32] Borrelli, E. et al. Plasma concentrations of cytokines, their soluble receptors, and antioxidant vitamins can predict the development of multiple organ failure in patients at risk. *Crit. Care Med.* **24**, 392–397 (1996). 8625625 10.1097/00003246-199603000-00006

[33] Liuzzo, G. et al. The prognostic value of C-reactive protein and serum amyloid a protein in severe unstable angina. *N. Engl. J. Med.* **331**, 417–424 (1994). 7880233 10.1056/NEJM199408183310701

[34] Ridker, P. M., Hennekens, C. H., Buring, J. E. & Rifai, N. C-reactive protein and other markers of inflammation in the prediction of cardiovascular disease in women. *N. Engl. J. Med.* **342**, 836–843 (2000). 10733371 10.1056/NEJM200003233421202

[35] Ridker, P. M., Rifai, N., Stampfer, M. J. & Hennekens, C. H. Plasma concentration of interleukin-6 and the risk of future myocardial infarction among apparently healthy men. *Circulation* **101**, 1767–1772 (2000). 10769275 10.1161/01.cir.101.15.1767

[36] Ridker, P. M. et al. Elevation of tumor necrosis factor-alpha and increased risk of recurrent coronary events after myocardial infarction. *Circulation* **101**, 2149–2153 (2000). 10801754 10.1161/01.cir.101.18.2149

[37] Roman, M. J. et al. Prevalence and correlates of accelerated atherosclerosis in systemic lupus erythematosus. *N. Engl. J. Med.* **349**, 2399–2406 (2003). 14681505 10.1056/NEJMoa035471

[38] Moos, M. P. et al. The lamina adventitia is the major site of immune cell accumulation in standard chow-fed apolipoprotein E-deficient mice. *Arterioscler Thromb. Vasc. Biol.* **25**, 2386–2391 (2005). 16179593 10.1161/01.ATV.0000187470.31662.fe

[39] Hu, D. et al. Artery tertiary lymphoid organs control aorta immunity and protect against atherosclerosis via vascular smooth muscle cell lymphotoxin β receptors. *Immunity* **42**, 1100–1115 (2015). 26084025 10.1016/j.immuni.2015.05.015 PMC4678289

[40] Depuydt, M. A. C. et al. Microanatomy of the human atherosclerotic plaque by single-cell transcriptomics. *Circ. Res.* **127**, 1437–1455 (2020). 32981416 10.1161/CIRCRESAHA.120.316770 PMC7641189

[41] Sun, X. et al. Meta-analysis of single-cell RNA-Seq data reveals the mechanism of formation and heterogeneity of tertiary lymphoid organ in vascular disease. *Arterioscler Thromb. Vasc. Biol.* **43**, 1867–1886 (2023). 37589134 10.1161/ATVBAHA.123.318762 PMC10521807

[42] Kuppe, C. et al. Spatial multi-omic map of human myocardial infarction. *Nature* **608**, 766–777 (2022). 35948637 10.1038/s41586-022-05060-x PMC9364862

[43] Drobni, Z. D. et al. Association between immune checkpoint inhibitors with cardiovascular events and atherosclerotic plaque. *Circulation* **142**, 2299–2311 (2020). 33003973 10.1161/CIRCULATIONAHA.120.049981 PMC7736526

[44] Gao, T. A. & Chen, Y. Y. T cells to fix a broken heart. *Science* **375**, 23–24 (2022). 34990255 10.1126/science.abn0851

[45] Aghajanian, H. et al. Targeting cardiac fibrosis with engineered T cells. *Nature* **573**, 430–433 (2019). 31511695 10.1038/s41586-019-1546-z PMC6752964

[46] Ridker, P. M. et al. Antiinflammatory therapy with canakinumab for atherosclerotic disease. *N. Engl. J. Med.* **377**, 1119–1131 (2017). 28845751 10.1056/NEJMoa1707914

[47] Tardif, J. C. et al. Efficacy and safety of low-dose colchicine after myocardial infarction. *N. Engl. J. Med.* **381**, 2497–2505 (2019). 31733140 10.1056/NEJMoa1912388

[48] Sun, K., Li, Y. Y. & Jin, J. A double-edged sword of immuno-microenvironment in cardiac homeostasis and injury repair. *Signal. Transduct. Target Ther.* **6**, 79 (2021). 33612829 10.1038/s41392-020-00455-6 PMC7897720

[49] Simoes, F. C. & Riley, P. R. Immune cells in cardiac repair and regeneration. *Development* **149**, dev199906 (2022). 35502777 10.1242/dev.199906 PMC9124571

[50] Litvinukova, M. et al. Cells of the adult human heart. *Nature* **588**, 466–472 (2020). 32971526 10.1038/s41586-020-2797-4 PMC7681775

[51] Yu, X. et al. Innate lymphoid cells promote recovery of ventricular function after myocardial infarction. *J. Am. Coll. Cardiol.* **78**, 1127–1142 (2021). 34503682 10.1016/j.jacc.2021.07.018 PMC8434674

[52] Adamo, L., et al. Modulation of subsets of cardiac B lymphocytes improves cardiac function after acute injury. *JCI Insight* **3**, e120137 (2018). 29875326 10.1172/jci.insight.120137 PMC6124442

[53] Epelman, S. et al. Embryonic and adult-derived resident cardiac macrophages are maintained through distinct mechanisms at steady state and during inflammation. *Immunity* **40**, 91–104 (2014). 24439267 10.1016/j.immuni.2013.11.019 PMC3923301

[54] Perdiguero, E. G. et al. The origin of tissue-resident macrophages: when an erythro-myeloid progenitor is an erythro-myeloid progenitor. *Immunity* **43**, 1023–1024 (2015). 26682973 10.1016/j.immuni.2015.11.022

[55] Molawi, K. et al. Progressive replacement of embryo-derived cardiac macrophages with age. *J. Exp. Med.* **211**, 2151–2158 (2014). 25245760 10.1084/jem.20140639 PMC4203946

[56] Lee, C. Z. W. & Ginhoux, F. Biology of resident tissue macrophages. *Development* **149**, 8 (2022). 10.1242/dev.200270 35502781

[57] Davies, L. C., Jenkins, S. J., Allen, J. E. & Taylor, P. R. Tissue-resident macrophages. *Nat. Immunol.* **14**, 986–995 (2013). 24048120 10.1038/ni.2705 PMC4045180

[58] Daems, W. T. & Brederoo, P. The fine structure and peroxidase activity of resident and exudate peritoneal macrophages in the guinea pig. In The Reticuloendothelial System and Immune Phenomena (eds. Di Luzio, N. R. & Flemming, K. B. P) Advances in Experimental Medicine and Biology 15, 19–31 (Springer, Boston, MA, 1971). 10.1007/978-1-4684-3204-6_3.

[59] Sabin, F., Doan, C. A. & Cunningham, R. S. Discrimination of two types of phagocytic cells in the connective tissues by the supravital technique. *Embryol* **16**, 125–162 (1925).

[60] Randolph, G. J., Ochando, J. & Partida-Sanchez, S. Migration of dendritic cell subsets and their precursors. *Annu. Rev. Immunol.* **26**, 293–316 (2008). 18045026 10.1146/annurev.immunol.26.021607.090254

[61] van Furth, R. & Cohn, Z. A. The origin and kinetics of mononuclear phagocytes. *J. Exp. Med.* **128**, 415–435 (1968). 5666958 10.1084/jem.128.3.415 PMC2138527

[62] van Furth, R. et al. The mononuclear phagocyte system: a new classification of macrophages, monocytes, and their precursor cells. *Bull. World Health Organ* **46**, 845–852 (1972). 4538544 PMC2480884

[63] Parwaresch, M. R. & Wacker, H. H. Origin and kinetics of resident tissue macrophages. Parabiosis studies with radiolabelled leucocytes. *Cell Tissue Kinet.* **17**, 25–39 (1984). 6692464 10.1111/j.1365-2184.1984.tb00565.x

[64] Lafuse, W. P., Wozniak, D. J. & Rajaram, M. V. S. Role of cardiac macrophages on cardiac inflammation, fibrosis and tissue repair. *Cells* **10**, 51 (2020). 33396359 10.3390/cells10010051 PMC7824389

[65] Hulsmans, M. et al. Macrophages facilitate electrical conduction in the heart. *Cell* **169**, 510–522 e520 (2017). 28431249 10.1016/j.cell.2017.03.050 PMC5474950

[66] Sugita, J., et al. Cardiac macrophages prevent sudden death during heart stress. *Nat. Commun.* **12**, 1910 (2021). 33771995 10.1038/s41467-021-22178-0 PMC7997915

[67] Wang, H. X. et al. CD1d-dependent natural killer T cells attenuate angiotensin II-induced cardiac remodelling via IL-10 signalling in mice. *Cardiovasc. Res.* **115**, 83–93 (2019). 29939225 10.1093/cvr/cvy164

[68] Ong, S., Rose, N. R. & Cihakova, D. Natural killer cells in inflammatory heart disease. *Clin. Immunol.* **175**, 26–33 (2017). 27894980 10.1016/j.clim.2016.11.010 PMC5315604

[69] Bouchentouf, M. et al. Induction of cardiac angiogenesis requires killer cell lectin-like receptor 1 and alpha4beta7 integrin expression by NK cells. *J. Immunol.* **185**, 7014–7025 (2010). 20971926 10.4049/jimmunol.1001888

[70] Bouvain, P. et al. Non-invasive mapping of systemic neutrophil dynamics upon cardiovascular injury. *Nat. Cardiovasc. Res.* **2**, 126–143 (2023). 39196054 10.1038/s44161-022-00210-w PMC11357992

[71] Phillipson, M. & Kubes, P. The neutrophil in vascular inflammation. *Nat. Med.* **17**, 1381–1390 (2011). 22064428 10.1038/nm.2514 PMC7095830

[72] Ofori, E. A. et al. Human blood neutrophils generate ROS through FcgammaR-signaling to mediate protection against febrile P. falciparum malaria. *Commun. Biol.* **6**, 743 (2023). 37463969 10.1038/s42003-023-05118-0 PMC10354059

[73] Malamud, M. et al. Recognition and control of neutrophil extracellular trap formation by MICL. *Nature* **633**, 442–450 (2024). 39143217 10.1038/s41586-024-07820-3 PMC11390483

[74] Cowan, K. N., Jones, P. L. & Rabinovitch, M. Elastase and matrix metalloproteinase inhibitors induce regression, and tenascin-C antisense prevents progression, of vascular disease. *J. Clin. Investig.* **105**, 21–34 (2000). 10619858 10.1172/JCI6539 PMC382582

[75] Christoffersson, G. et al. VEGF-A recruits a proangiogenic MMP-9-delivering neutrophil subset that induces angiogenesis in transplanted hypoxic tissue. *Blood* **120**, 4653–4662 (2012). 22966168 10.1182/blood-2012-04-421040 PMC3512240

[76] Kim, C., et al. Spatiotemporal control of neutrophil fate to tune inflammation and repair for myocardial infarction therapy. *Nat. Commun.* **15**, 8481 (2024). 39353987 10.1038/s41467-024-52812-6 PMC11445496

[77] Dao Nyesiga, G., et al. Tolerogenic dendritic cells generated in vitro using a novel protocol mimicking mucosal tolerance mechanisms represent a potential therapeutic cell platform for induction of immune tolerance. *Front. Immunol.* **14**, 1045183 (2023). 37901231 10.3389/fimmu.2023.1045183 PMC10613069

[78] Forte, E. et al. Cross-priming dendritic cells exacerbate immunopathology after ischemic tissue damage in the heart. *Circulation* **143**, 821–836 (2021). 33297741 10.1161/CIRCULATIONAHA.120.044581 PMC7899721

[79] Ma, Y. et al. Efferocytosis in dendritic cells: an overlooked immunoregulatory process. *Front. Immunol.* **15**, 1415573 (2024). 38835772 10.3389/fimmu.2024.1415573 PMC11148234

[80] Nayer, B., et al. Local administration of regulatory T cells promotes tissue healing. *Nat. Commun.* **15**, 7863 (2024). 39251592 10.1038/s41467-024-51353-2 PMC11383969

[81] Blanton, R. M., Carrillo-Salinas, F. J. & Alcaide, P. T-cell recruitment to the heart: friendly guests or unwelcome visitors?. *Am. J. Physiol. Heart Circ. Physiol.* **317**, H124–H140 (2019). 31074651 10.1152/ajpheart.00028.2019 PMC6692732

[82] Huang, F. et al. B cell subsets contribute to myocardial protection by inducing neutrophil apoptosis after ischemia and reperfusion. *JCI Insight*. **9**, (2024). 10.1172/jci.insight.167201 PMC10967377 38290007

[83] Porsch, F., Mallat, Z. & Binder, C. J. Humoral immunity in atherosclerosis and myocardial infarction: from B cells to antibodies. *Cardiovasc. Res.* **117**, 2544–2562 (2021). 34450620 10.1093/cvr/cvab285

[84] Garcia-Rivas, G. et al. The role of B cells in heart failure and implications for future immunomodulatory treatment strategies. *ESC Heart Fail.* **7**, 1387–1399 (2020). 32533765 10.1002/ehf2.12744 PMC7373901

[85] Becker, N. P., Goettel, P., Mueller, J., Wallukat, G. & Schimke, I. Functional autoantibody diseases: Basics and treatment related to cardiomyopathies. *Front. Biosci.* **24**, 48–95 (2019). 10.2741/4709 30468647

[86] Jiao, J. et al. Regulatory B cells improve ventricular remodeling after myocardial infarction by modulating monocyte migration. *Basic Res. Cardiol.* **116**, 46 (2021). 34302556 10.1007/s00395-021-00886-4 PMC8310480

[87] Tan, Y. et al. Murine neonatal cardiac B cells promote cardiomyocyte proliferation and heart regeneration. *NPJ Regen. Med.* **8**, 7 (2023). 36774363 10.1038/s41536-023-00282-7 PMC9922252

[88] Bermea, K. C. et al. Myocardial B cells have specific gene expression and predicted interactions in dilated cardiomyopathy and arrhythmogenic right ventricular cardiomyopathy. *Front. Immunol.* **15**, 1327372 (2024). 38736889 10.3389/fimmu.2024.1327372 PMC11082303

[89] Oduro, P. K. et al. The cGAS-STING signaling in cardiovascular and metabolic diseases: Future novel target option for pharmacotherapy. *Acta Pharmacol. Sin. B* **12**, 50–75 (2022). 10.1016/j.apsb.2021.05.011 PMC8799861 35127372

[90] Bullen, C. K. et al. MDA5 RNA-sensing pathway activation by Mycobacterium tuberculosis promotes innate immune subversion and pathogen survival. *JCI Insight* **8**, e166242 (2023). 37725440 10.1172/jci.insight.166242 PMC10619499

[91] Caneparo, V., Landolfo, S., Gariglio, M. & De Andrea, M. The absent in melanoma 2-like receptor IFN-inducible protein 16 as an inflammasome regulator in systemic lupus erythematosus: the dark side of sensing microbes. *Front. Immunol.* **9**, 1180 (2018). 29892303 10.3389/fimmu.2018.01180 PMC5985366

[92] Doran, A. C., Yurdagul, A. Jr. & Tabas, I. Efferocytosis in health and disease. *Nat. Rev. Immunol.* **20**, 254–267 (2020). 31822793 10.1038/s41577-019-0240-6 PMC7667664

[93] Arandjelovic, S. & Ravichandran, K. S. Phagocytosis of apoptotic cells in homeostasis. *Nat. Immunol.* **16**, 907–917 (2015). 26287597 10.1038/ni.3253 PMC4826466

[94] Wang, X. et al. Targeting regulatory T cells for cardiovascular diseases. *Front. Immunol.* **14**, 1126761 (2023). 36911741 10.3389/fimmu.2023.1126761 PMC9995594

[95] Vignali, D. A., Collison, L. W. & Workman, C. J. How regulatory T cells work. *Nat. Rev. Immunol.* **8**, 523–532 (2008). 18566595 10.1038/nri2343 PMC2665249

[96] Zhang, N. et al. The role of apoptosis in the development and function of T lymphocytes. *Cell Res.* **15**, 749–769 (2005). 16246265 10.1038/sj.cr.7290345

[97] Holodick, N. E., Rodriguez-Zhurbenko, N. & Hernandez, A. M. Defining natural antibodies. *Front. Immunol.* **8**, 872 (2017). 28798747 10.3389/fimmu.2017.00872 PMC5526850

[98] Harris, E. Majority of People Live With Uncontrolled Hypertension Worldwide. *JAMA* **330**, 1515 (2023). 37792401 10.1001/jama.2023.19204

[99] Wen, X. et al. The Minhang Pediatric Biobank cohort study: protocol overview and baseline characteristics. *BMC Pediatr.* **24**, 282 (2024). 38678186 10.1186/s12887-024-04763-6 PMC11055290

[100] Schutte, A. E. et al. Addressing global disparities in blood pressure control: perspectives of the International Society of Hypertension. *Cardiovasc. Res.* **119**, 381–409 (2023). 36219457 10.1093/cvr/cvac130 PMC9619669

[101] NCD Risk Factor Collaboration (NCD-RisC) Worldwide trends in hypertension prevalence and progress in treatment and control from 1990 to 2019: a pooled analysis of 1201 population-representative studies with 104 million participants. *Lancet* **398**, 957–980 (2021). 34450083 10.1016/S0140-6736(21)01330-1 PMC8446938

[102] Evangelou, E. et al. Genetic analysis of over 1 million people identifies 535 new loci associated with blood pressure traits. *Nat. Genet.* **50**, 1412–1425 (2018). 30224653 10.1038/s41588-018-0205-x PMC6284793

[103] Garshick, M. S., Ward, N. L., Krueger, J. G. & Berger, J. S. Cardiovascular risk in patients with psoriasis: JACC review topic of the week. *J. Am. Coll. Cardiol.* **77**, 1670–1680 (2021). 33795041 10.1016/j.jacc.2021.02.009 PMC8168628

[104] Panoulas, V. F. et al. Prevalence and associations of hypertension and its control in patients with rheumatoid arthritis. *Rheumatology* **46**, 1477–1482 (2007). 17704521 10.1093/rheumatology/kem169

[105] Munoz Aguilera, E. et al. Periodontitis is associated with hypertension: a systematic review and meta-analysis. *Cardiovasc. Res.* **116**, 28–39 (2020). 31549149 10.1093/cvr/cvz201

[106] Eke, P. I. et al. Periodontitis in US Adults: National health and nutrition examination survey 2009-2014. *J. Am. Dent. Assoc.* **149**, 576–588.e576 (2018). 29957185 10.1016/j.adaj.2018.04.023 PMC8094373

[107] Meng, X. et al. Regulatory T cells in cardiovascular diseases. *Nat. Rev. Cardiol.* **13**, 167–179 (2016). 26525543 10.1038/nrcardio.2015.169 PMC11849084

[108] Xia, Y., et al. Role of Treg cell subsets in cardiovascular disease pathogenesis and potential therapeutic targets. *Front. Immunol.* **15**, 1331609 (2024). 38558816 10.3389/fimmu.2024.1331609 PMC10978666

[109] Guzik, T. J., Nosalski, R., Maffia, P. & Drummond, G. R. Immune and inflammatory mechanisms in hypertension. *Nat. Rev. Cardiol.* **21**, 396–416 (2024). 38172242 10.1038/s41569-023-00964-1

[110] Steinman, R. M., Hawiger, D. & Nussenzweig, M. C. Tolerogenic dendritic cells. *Annu. Rev. Immunol.* **21**, 685–711 (2003). 12615891 10.1146/annurev.immunol.21.120601.141040

[111] Higaki, A. & Mogi, M. Dendritic cells as potential initiators of immune-mediated hypertensive disorders. *Hypertens. Res.* **45**, 527–529 (2021). 34961789 10.1038/s41440-021-00830-y

[112] Barbaro, N. R. et al. Dendritic cell amiloride-sensitive channels mediate sodium-induced inflammation and hypertension. *Cell Rep.* **21**, 1009–1020 (2017). 29069584 10.1016/j.celrep.2017.10.002 PMC5674815

[113] Carnevale, D., et al. A cholinergic-sympathetic pathway primes immunity in hypertension and mediates brain-to-spleen communication. *Nat. Commun.* **7**, 13035 (2016). 27676657 10.1038/ncomms13035 PMC5052663

[114] Pavlov, V. A. & Tracey, K. J. Neural regulation of immunity: molecular mechanisms and clinical translation. *Nat. Neurosci.* **20**, 156–166 (2017). 28092663 10.1038/nn.4477

[115] Nguyen, B. A., Alexander, M. R. & Harrison, D. G. Immune mechanisms in the pathophysiology of hypertension. *Nat. Rev. Nephrol.* **20**, 530–540 (2024). 38658669 10.1038/s41581-024-00838-w PMC12060254

[116] Hahn, C. & Schwartz, M. A. Mechanotransduction in vascular physiology and atherogenesis. *Nat. Rev. Mol. Cell Biol.* **10**, 53–62 (2009). 19197332 10.1038/nrm2596 PMC2719300

[117] Harrison, D. G. & Patrick, D. M. Immune mechanisms in hypertension. *Hypertension* **81**, 1659–1674 (2024). 38881474 10.1161/HYPERTENSIONAHA.124.21355 PMC11254551

[118] Itani, H. A. et al. Activation of human T cells in hypertension: studies of humanized mice and hypertensive humans. *Hypertension* **68**, 123–132 (2016). 27217403 10.1161/HYPERTENSIONAHA.116.07237 PMC4900908

[119] Wang, X., et al. Single-cell transcriptome profiling reveals enriched memory T-cell subpopulations in hypertension. *Front. Cell Dev. Biol.* **11**, 1132040 (2023). 37009484 10.3389/fcell.2023.1132040 PMC10060952

[120] Pober, J. S., Merola, J., Liu, R. & Manes, T. D. Antigen presentation by vascular cells. *Front. Immunol.* **8**, 1907 (2017). 29312357 10.3389/fimmu.2017.01907 PMC5744398

[121] Didion, S. P. et al. Endogenous interleukin-10 inhibits angiotensin II-induced vascular dysfunction. *Hypertension* **54**, 619–624 (2009). 19620507 10.1161/HYPERTENSIONAHA.109.137158 PMC2861503

[122] Libby, P. Inflammation in atherosclerosis. *Nature* **420**, 868–874 (2002). 12490960 10.1038/nature01323

[123] Trimm, E. & Red-Horse, K. Vascular endothelial cell development and diversity. *Nat. Rev. Cardiol.* **20**, 197–210 (2023). 36198871 10.1038/s41569-022-00770-1 PMC9533272

[124] Neubauer, K. & Zieger, B. Endothelial cells and coagulation. *Cell Tissue Res.* **387**, 391–398 (2022). 34014399 10.1007/s00441-021-03471-2 PMC8975780

[125] Pober, J. S. & Sessa, W. C. Evolving functions of endothelial cells in inflammation. *Nat. Rev. Immunol.* **7**, 803–815 (2007). 17893694 10.1038/nri2171

[126] He, C. et al. The role of immune cells in different stages of atherosclerosis. *Int. J. Med. Sci.* **21**, 1129–1143 (2024). 38774746 10.7150/ijms.94570 PMC11103388

[127] Hansson, G. K. & Hermansson, A. The immune system in atherosclerosis. *Nat. Immunol.* **12**, 204–212 (2011). 21321594 10.1038/ni.2001

[128] Zernecke, A. et al. Meta-analysis of leukocyte diversity in atherosclerotic mouse aortas. *Circ. Res.* **127**, 402–426 (2020). 32673538 10.1161/CIRCRESAHA.120.316903 PMC7371244

[129] Pan, H. et al. Single-cell genomics reveals a novel cell state during smooth muscle cell phenotypic switching and potential therapeutic targets for atherosclerosis in mouse and human. *Circulation* **142**, 2060–2075 (2020). 32962412 10.1161/CIRCULATIONAHA.120.048378 PMC8104264

[130] Wirka, R. C. et al. Atheroprotective roles of smooth muscle cell phenotypic modulation and the TCF21 disease gene as revealed by single-cell analysis. *Nat. Med.* **25**, 1280–1289 (2019). 31359001 10.1038/s41591-019-0512-5 PMC7274198

[131] Feil, S. et al. Transdifferentiation of vascular smooth muscle cells to macrophage-like cells during atherogenesis. *Circ. Res.* **115**, 662–667 (2014). 25070003 10.1161/CIRCRESAHA.115.304634

[132] Libby, P. The changing landscape of atherosclerosis. *Nature* **592**, 524–533 (2021). 33883728 10.1038/s41586-021-03392-8

[133] Bairey Merz, C. N., Pepine, C. J., Walsh, M. N. & Fleg, J. L. Ischemia and no obstructive coronary artery disease (INOCA): developing evidence-based therapies and research agenda for the next decade. *Circulation* **135**, 1075–1092 (2017). 28289007 10.1161/CIRCULATIONAHA.116.024534 PMC5385930

[134] Nishimiya, K. et al. Mechanisms of coronary artery spasm. *Eur. Cardiol.* **18**, e39 (2023). 37456775 10.15420/ecr.2022.55 PMC10345984

[135] Mensah, G. A. et al. Global burden of cardiovascular diseases and risks, 1990-2022. *J. Am. Coll. Cardiol.* **82**, 2350–2473 (2023). 38092509 10.1016/j.jacc.2023.11.007 PMC7615984

[136] Dittrich, A. & Lauridsen, H. Myocardial infarction and the immune response - Scarring or regeneration? A comparative look at mammals and popular regenerating animal models. *J. Immunol. Regen. Med.* **4**, 100016 (2019).

[137] Santos-Zas, I., Lemarie, J., Tedgui, A. & Ait-Oufella, H. Adaptive immune responses contribute to post-ischemic cardiac remodeling. *Front. Cardiovasc. Med.* **5**, 198 (2018). 30687720 10.3389/fcvm.2018.00198 PMC6335242

[138] Xu, S. W., Xu, C., Xu, J. H., Zhang, K. & Zhang, H. J. Macrophage heterogeneity and its impact on myocardial ischemia-reperfusion injury: an integrative review. *J. Inflamm. Res.* **16**, 5971–5987 (2023). 38088942 10.2147/JIR.S436560 PMC10712254

[139] Zouggari, Y. et al. B lymphocytes trigger monocyte mobilization and impair heart function after acute myocardial infarction. *Nat. Med.* **19**, 1273–1280 (2013). 24037091 10.1038/nm.3284 PMC4042928

[140] Feng, Q., et al. The role of major immune cells in myocardial infarction. *Front. Immunol.* **13**, 1084460 (2022). 36741418 10.3389/fimmu.2022.1084460 PMC9892933

[141] Tang, T. T. et al. Regulatory T cells ameliorate cardiac remodeling after myocardial infarction. *Basic Res. Cardiol.* **107**, 232 (2012). 22189560 10.1007/s00395-011-0232-6

[142] Kumar, V., Prabhu, S. D. & Bansal, S. S. CD4(+) T-lymphocytes exhibit biphasic kinetics post-myocardial infarction. *Front. Cardiovasc. Med.* **9**, 992653 (2022). 36093172 10.3389/fcvm.2022.992653 PMC9452745

[143] Nakamura, M. & Sadoshima, J. Mechanisms of physiological and pathological cardiac hypertrophy. *Nat. Rev. Cardiol.* **15**, 387–407 (2018). 29674714 10.1038/s41569-018-0007-y

[144] Frantz, S. et al. Left ventricular remodelling post-myocardial infarction: pathophysiology, imaging, and novel therapies. *Eur. Heart J.* **43**, 2549–2561 (2022). 35511857 10.1093/eurheartj/ehac223 PMC9336586

[145] Robson, P. M. et al. MR/PET imaging of the cardiovascular system. *JACC Cardiovasc. Imaging* **10**, 1165–1179 (2017). 28982570 10.1016/j.jcmg.2017.07.008 PMC6415529

[146] Barros-Gomes, S. et al. Cardiac remodeling in acute myocardial infarction: Prospective insights from multimodality ultrasound imaging. *Echocardiography* **38**, 2032–2042 (2021). 34845767 10.1111/echo.15245 PMC9059245

[147] Martinez, M. W. et al. Exercise-induced cardiovascular adaptations and approach to exercise and cardiovascular disease: JACC state-of-the-art review. *J. Am. Coll. Cardiol.* **78**, 1453–1470 (2021). 34593128 10.1016/j.jacc.2021.08.003

[148] De Haas, S. et al. Cardiac remodeling in normotensive pregnancy and in pregnancy complicated by hypertension: systematic review and meta-analysis. *Ultrasound Obstet. Gynecol.* **50**, 683–696 (2017). 28078751 10.1002/uog.17410

[149] Deb, A. & Ubil, E. Cardiac fibroblast in development and wound healing. *J. Mol. Cell Cardiol.* **70**, 47–55 (2014). 24625635 10.1016/j.yjmcc.2014.02.017 PMC4028446

[150] Jitmana, R. et al. Role of cardiac mast cells in exercise training-mediated cardiac remodeling in angiotensin II-infused ovariectomized rats. *Life Sci.* **219**, 209–218 (2019). 30658099 10.1016/j.lfs.2019.01.018

[151] Lavine, K. J. et al. Distinct macrophage lineages contribute to disparate patterns of cardiac recovery and remodeling in the neonatal and adult heart. *Proc. Natl. Acad. Sci. USA* **111**, 16029–16034 (2014). 25349429 10.1073/pnas.1406508111 PMC4234568

[152] Wang, Z. et al. Mechanistic basis of neonatal heart regeneration revealed by transcriptome and histone modification profiling. *Proc. Natl. Acad. Sci. USA* **116**, 18455–18465 (2019). 31451669 10.1073/pnas.1905824116 PMC6744882

[153] Borges, D. et al. Exercise training and cardiac remodeling sports, health and exercise medicine. *Exerc. Sport Sci. Rev*. **50**, 137–144 (2019).

[154] Yin, A., et al. Exercise-derived peptide protects against pathological cardiac remodeling. *EBioMedicine* **82**, 104164 (2022). 35843176 10.1016/j.ebiom.2022.104164 PMC9297110

[155] Du, H. et al. Tuning immunity through tissue mechanotransduction. *Nat. Rev. Immunol.* **23**, 174–188 (2023). 35974148 10.1038/s41577-022-00761-w PMC9379893

[156] Yap, J. et al. Macrophages in cardiac remodelling after myocardial infarction. *Nat. Rev. Cardiol.* **20**, 373–385 (2023). 36627513 10.1038/s41569-022-00823-5

[157] Nian, M., Lee, P., Khaper, N. & Liu, P. Inflammatory cytokines and postmyocardial infarction remodeling. *Circ. Res.* **94**, 1543–1553 (2004). 15217919 10.1161/01.RES.0000130526.20854.fa

[158] Strassheim, D., et al. Role of inflammatory cell subtypes in heart Failure. *J. Immunol. Res.* **2019**, 2164017 (2019). 31565659 10.1155/2019/2164017 PMC6745095

[159] Kologrivova, I., Shtatolkina, M., Suslova, T. & Ryabov, V. Cells of the immune system in cardiac remodeling: main players in resolution of inflammation and repair after myocardial infarction. *Front. Immunol.* **12**, 664457 (2021). 33868315 10.3389/fimmu.2021.664457 PMC8050340

[160] De Angelis, E. et al. Cross-talk between neurohormonal pathways and the immune system in heart failure: a review of the literature. *Int. J. Mol. Sci*. **20**, 1698 (2019). 10.3390/ijms20071698 PMC6480265 30959745

[161] Nishida, K. & Otsu, K. Inflammation and metabolic cardiomyopathy. *Cardiovasc. Res.* **113**, 389–398 (2017). 28395010 10.1093/cvr/cvx012

[162] Hotamisligil, G. S. Inflammation and metabolic disorders. *Nature* **444**, 860–867 (2006). 17167474 10.1038/nature05485

[163] Wellen, K. E. Inflammation, stress, and diabetes. *J. Clin. Investig.* **115**, 1111–1119 (2005). 15864338 10.1172/JCI25102 PMC1087185

[164] Bahrar, H. et al. Innate immune memory in cardiometabolic disease. *Cardiovasc. Res.* **119**, 2774–2786 (2024). 36795085 10.1093/cvr/cvad030 PMC10874278

[165] Jia, G. et al. Uric acid promotes left ventricular diastolic dysfunction in mice fed a Western diet. *Hypertension* **65**, 531–539 (2015). 25489061 10.1161/HYPERTENSIONAHA.114.04737 PMC4370431

[166] Eguchi, K. & Nagai, R. Islet inflammation in type 2 diabetes and physiology. *J. Clin. Investig.* **127**, 14–23 (2017). 28045399 10.1172/JCI88877 PMC5199688

[167] Knapp, M., Tu, X. & Wu, R. Vascular endothelial dysfunction, a major mediator in diabetic cardiomyopathy. *Acta Pharmacol. Sin.* **40**, 1–8 (2019). 29867137 10.1038/s41401-018-0042-6 PMC6318313

[168] Abumrad, N. A. et al. Endothelial cell receptors in tissue lipid uptake and metabolism. *Circ. Res.* **128**, 433–450 (2021). 33539224 10.1161/CIRCRESAHA.120.318003 PMC7959116

[169] Zhang, Y. et al. Fibroblast-specific activation of Rnd3 protects against cardiac remodeling in diabetic cardiomyopathy via suppression of Notch and TGF-beta signaling. *Theranostics* **12**, 7250–7266 (2022). 36438502 10.7150/thno.77043 PMC9691359

[170] Ballasy, N. N., et al. Potential role of epicardial adipose tissue in coronary artery endothelial cell dysfunction in type 2 diabetes. *FASEB J.* **35**, e21878 (2021). 34469050 10.1096/fj.202100684RR

[171] Wegner, M., Neddermann, D., Piorunska-Stolzmann, M. & Jagodzinski, P. P. Role of epigenetic mechanisms in the development of chronic complications of diabetes. *Diabetes Res. Clin. Pr.* **105**, 164–175 (2014). 10.1016/j.diabres.2014.03.019 24814876

[172] Kenny, H. C. & Abel, E. D. Heart failure in type 2 diabetes mellitus. *Circ. Res.* **124**, 121–141 (2019). 30605420 10.1161/CIRCRESAHA.118.311371 PMC6447311

[173] Isselbacher, E. M. et al. 2022 ACC/AHA guideline for the diagnosis and management of aortic disease: a report of the American Heart Association/American College of Cardiology Joint Committee on Clinical Practice Guidelines. *Circulation* **146**, e334–e482 (2022). 10.1161/CIR.0000000000001106 PMC9876736 36322642

[174] Cho, M. J., Lee, M. R. & Park, J. G. Aortic aneurysms: current pathogenesis and therapeutic targets. *Exp. Mol. Med.* **55**, 2519–2530 (2023). 38036736 10.1038/s12276-023-01130-w PMC10766996

[175] Zhao, G. et al. Single-cell RNA sequencing reveals the cellular heterogeneity of aneurysmal infrarenal abdominal aorta. *Cardiovasc. Res.* **117**, 1402–1416 (2021). 32678909 10.1093/cvr/cvaa214 PMC8064434

[176] Li, Y. et al. Single-cell transcriptome analysis reveals dynamic cell populations and differential gene expression patterns in control and aneurysmal human aortic tissue. *Circulation* **142**, 1374–1388 (2020). 33017217 10.1161/CIRCULATIONAHA.120.046528 PMC7539140

[177] Smigiel, K. S., Srivastava, S., Stolley, J. M. & Campbell, D. J. Regulatory T-cell homeostasis: steady-state maintenance and modulation during inflammation. *Immunol. Rev.* **259**, 40–59 (2014). 24712458 10.1111/imr.12170 PMC4083836

[178] Dri, E. et al. Inflammatory mediators of endothelial dysfunction. *Life*. **13**, 1420 (2023). 10.3390/life13061420 PMC10305352 37374202

[179] Bobryshev, Y. V. Dendritic cells and their role in atherogenesis. *Lab Investig.* **90**, 970–984 (2010). 20458277 10.1038/labinvest.2010.94

[180] Meng, Q. et al. Laminar shear stress inhibits inflammation by activating autophagy in human aortic endothelial cells through HMGB1 nuclear translocation. *Commun. Biol.* **5**, 425 (2022). 35523945 10.1038/s42003-022-03392-y PMC9076621

[181] Xue, Y. et al. Macrophages regulate vascular smooth muscle cell function during atherosclerosis progression through IL-1beta/STAT3 signaling. *Commun. Biol.* **5**, 1316 (2022). 36456628 10.1038/s42003-022-04255-2 PMC9715630

[182] Kenney, M. J. & Ganta, C. K. Autonomic nervous system and immune system interactions. *Compr. Physiol.* **4**, 1177–1200 (2014). 24944034 10.1002/cphy.c130051 PMC4374437

[183] Yuan, Z. et al. Abdominal aortic aneurysm: roles of inflammatory cells. *Front. Immunol.* **11**, 609161 (2020). 33613530 10.3389/fimmu.2020.609161 PMC7886696

[184] Davis, F. M. & Gallagher, K. A. Epigenetic mechanisms in monocytes/macrophages regulate inflammation in cardiometabolic and vascular disease. *Arterioscler Thromb. Vasc. Biol.* **39**, 623–634 (2019). 30760015 10.1161/ATVBAHA.118.312135 PMC6438376

[185] Hernandez, G. E. et al. Aortic intimal resident macrophages are essential for maintenance of the non-thrombogenic intravascular state. *Nat. Cardiovasc. Res.* **1**, 67–84 (2022). 35599984 10.1038/s44161-021-00006-4 PMC9121812

[186] Choi, J. H. et al. Identification of antigen-presenting dendritic cells in mouse aorta and cardiac valves. *J. Exp. Med.* **206**, 497–505 (2009). 19221394 10.1084/jem.20082129 PMC2699134

[187] Banchereau, J. & Steinman, R. M. Dendritic cells and the control of immunity. *Nature* **392**, 245–252 (1998). 9521319 10.1038/32588

[188] Ma-Krupa, W. et al. Activation of arterial wall dendritic cells and breakdown of self-tolerance in giant cell arteritis. *J. Exp. Med.* **199**, 173–183 (2004). 14734523 10.1084/jem.20030850 PMC2211768

[189] Jauhiainen, S., Kiema, M., Hedman, M. & Laakkonen, J. P. Large Vessel Cell Heterogeneity and plasticity: focus in aortic aneurysms. *Arterioscler. Thromb. Vasc. Biol.* **42**, 811–818 (2022). 35587695 10.1161/ATVBAHA.121.316237

[190] Li, J., et al. Aorta regulatory T cells with a tissue-specific phenotype and function promote tissue repair through Tff1 in abdominal aortic aneurysms. *Adv. Sci.* **9**, e2104338 (2022). 10.1002/advs.202104338 PMC8948580 35332699

[191] Luan, Y. et al. Cardiac cell senescence: molecular mechanisms, key proteins and therapeutic targets. *Cell Death Discov.* **10**, 78 (2024). 38355681 10.1038/s41420-023-01792-5 PMC10866973

[192] Desdín-Micó, G. et al. T cells with dysfunctional mitochondria induce multimorbidity and premature senescence. *Science* **368**, 1371–1376 (2020). 32439659 10.1126/science.aax0860 PMC7616968

[193] Faust, H. J. et al. IL-17 and immunologically induced senescence regulate response to injury in osteoarthritis. *J. Clin. Investig.* **130**, 5493–5507 (2020). 32955487 10.1172/JCI134091 PMC7524483

[194] Ovadya, Y., et al. Impaired immune surveillance accelerates accumulation of senescent cells and aging. *Nat. Commun.* **9**, 5435 (2018). 30575733 10.1038/s41467-018-07825-3 PMC6303397

[195] Elyahu, Y., et al. Aging promotes reorganization of the CD4 T cell landscape toward extreme regulatory and effector phenotypes. *Sci. Adv.* **5**, eaaw8330 (2019). 31457092 10.1126/sciadv.aaw8330 PMC6703865

[196] Pereira, B. I. et al. Sestrins induce natural killer function in senescent-like CD8(+) T cells. *Nat. Immunol.* **21**, 684–694 (2020). 32231301 10.1038/s41590-020-0643-3 PMC10249464

[197] Tang, X., Li, P. H. & Chen, H. Z. Cardiomyocyte senescence and cellular communications within myocardial microenvironments. *Front. Endocrinol.* **11**, 280 (2020). 10.3389/fendo.2020.00280 PMC7253644 32508749

[198] He, A. & Shi, G. P. Mast cell chymase and tryptase as targets for cardiovascular and metabolic diseases. *Curr. Pharmacol. Des.* **19**, 1114–1125 (2013). 10.2174/1381612811319060012 PMC3921624 23016684

[199] Grim, J. C. et al. Secreted factors from proinflammatory macrophages promote an osteoblast-like phenotype in valvular interstitial cells. *Arterioscler. Thromb. Vasc. Biol.* **40**, e296–e308 (2020). 32938214 10.1161/ATVBAHA.120.315261 PMC7578003

[200] Bloom, S. I., Islam, M. T., Lesniewski, L. A. & Donato, A. J. Mechanisms and consequences of endothelial cell senescence. *Nat. Rev. Cardiol.* **20**, 38–51 (2023). 35853997 10.1038/s41569-022-00739-0 PMC10026597

[201] Gardner, S. E., Humphry, M., Bennett, M. R. & Clarke, M. C. Senescent vascular smooth muscle cells drive inflammation through an interleukin-1α-dependent senescence-associated secretory phenotype. *Arterioscler. Thromb. Vasc. Biol.* **35**, 1963–1974 (2015). 26139463 10.1161/ATVBAHA.115.305896 PMC4548545

[202] Grune, J., Yamazoe, M. & Nahrendorf, M. Electroimmunology and cardiac arrhythmia. *Nat. Rev. Cardiol.* **18**, 547–564 (2021). 33654273 10.1038/s41569-021-00520-9 PMC9703448

[203] Baksi, A. J., Kanaganayagam, G. S. & Prasad, S. K. Arrhythmias in viral myocarditis and pericarditis. *Card. Electrophysiol. Clin.* **7**, 269–281 (2015). 26002391 10.1016/j.ccep.2015.03.009

[204] Singer, M. et al. The third international consensus definitions for sepsis and septic shock (Sepsis-3). *JAMA* **315**, 801 (2016). 26903338 10.1001/jama.2016.0287 PMC4968574

[205] Dobrev, D. et al. Inflammatory signalling in atrial cardiomyocytes: a novel unifying principle in atrial fibrillation pathophysiology. *Nat. Rev. Cardiol.* **20**, 145–167 (2023). 36109633 10.1038/s41569-022-00759-w PMC9477170

[206] Lazzerini, P. E. et al. Cardioimmunology of arrhythmias: the role of autoimmune and inflammatory cardiac channelopathies. *Nat. Rev. Immunol.* **19**, 63–64 (2019). 30552386 10.1038/s41577-018-0099-y

[207] Xiao, G. Q., Hu, K. & Boutjdir, M. Direct inhibition of expressed cardiac L- and T-type calcium channels by IgG from mothers whose children have congenital heart block. *Circulation* **103**, 1599–1604 (2001). 11257091 10.1161/01.cir.103.11.1599

[208] Karnabi, E. et al. Congenital heart block: identification of autoantibody binding site on the extracellular loop (domain I, S5-S6) of α1D L-type Ca channel. *J. Autoimmun.* **34**, 80–86 (2010). 19640679 10.1016/j.jaut.2009.06.005 PMC2822065

[209] Lazzerini, P. E. et al. Anti-Ro/SSA antibodies blocking calcium channels as a potentially reversible cause of atrioventricular block in adults. *JACC Clin. Electrophysiol.* **9**, 1631–1648 (2023). 37227349 10.1016/j.jacep.2023.03.007

[210] Sethi, N. et al. Noninvasive fetal electrocardiography in the diagnosis of long QT syndrome: a case series. *Fetal Diagn. Ther.* **47**, 711–716 (2020). 32615554 10.1159/000508043 PMC7483932

[211] Yue, Y., Casadei, B. & Marín-García, J. Pathogenesis of the novel autoimmune-associated long-QT syndrome. *Circulation* **132**, 230–240 (2015). 25995318 10.1161/CIRCULATIONAHA.115.009800

[212] Lazzerini, P. E., et al. Arrhythmogenicity of anti-Ro/SSA antibodies in patients with torsades de pointes. *Circ. Arrhythm. Electrophysiol.* **9**, e003419 (2016). 27030700 10.1161/CIRCEP.115.003419

[213] Suzuki, S. et al. Cardiac involvements in myasthenia gravis associated with anti-KV1.4 antibodies. *Eur. J. Neurol.* **21**, 223–230 (2014). 23829303 10.1111/ene.12234

[214] Li, J. et al. Anti-KCNQ1 K channel autoantibodies increase IKs current and are associated with QT interval shortening in dilated cardiomyopathy. *Cardiovasc. Res.* **98**, 496–503 (2013). 23447643 10.1093/cvr/cvt046

[215] Pollack, A., Kontorovich, A. R., Fuster, V. & Dec, G. W. Viral myocarditis-diagnosis, treatment options, and current controversies. *Nat. Rev. Cardiol.* **12**, 670–680 (2015). 26194549 10.1038/nrcardio.2015.108

[216] Tschöpe, C. et al. Myocarditis and inflammatory cardiomyopathy: current evidence and future directions. *Nat. Rev. Cardiol.* **18**, 169–193 (2021). 33046850 10.1038/s41569-020-00435-x PMC7548534

[217] Koc, A. & Cagavi, E. Cardiac immunology: a new era for immune cells in the heart. *Adv. Exp. Med. Biol.* **1312**, 75–95 (2021). 32910424 10.1007/5584_2020_576

[218] Fang, M. et al. TRIM18 is a critical regulator of viral myocarditis and organ inflammation. *J. Biomed. Sci.* **29**, 55 (2022). 35909127 10.1186/s12929-022-00840-z PMC9339186

[219] Wang, J., et al. Loss of TRIM29 mitigates viral myocarditis by attenuating PERK-driven ER stress response in male mice. *Nat. Commun.* **15**, 3481 (2024). 38664417 10.1038/s41467-024-44745-x PMC11045800

[220] Pappritz, K. et al. Immunomodulation by adoptive regulatory T-cell transfer improves Coxsackievirus B3-induced myocarditis. *FASEB J.* **32**, 6066–6078 (2018). 10.1096/fj.201701408R 29863913

[221] Dubin, K., et al. Intestinal microbiome analyses identify melanoma patients at risk for checkpoint-blockade-induced colitis. *Nat. Commun.* **7**, 10391 (2016). 26837003 10.1038/ncomms10391 PMC4740747

[222] Kindermann, I. et al. Update on myocarditis. *J. Am. Coll. Cardiol.* **59**, 779–792 (2012). 22361396 10.1016/j.jacc.2011.09.074

[223] Sury, K., Perazella, M. A. & Shirali, A. C. Cardiorenal complications of immune checkpoint inhibitors. *Nat. Rev. Nephrol.* **14**, 571–588 (2018). 30013100 10.1038/s41581-018-0035-1

[224] Wang, S. et al. Perspectives of tumor-infiltrating lymphocyte treatment in solid tumors. *BMC Med.* **19**, 140 (2021). 34112147 10.1186/s12916-021-02006-4 PMC8194199

[225] Kantarjian, H. et al. Blinatumomab versus chemotherapy for Advanced Acute Lymphoblastic Leukemia. *N. Engl. J. Med.* **376**, 836–847 (2017). 28249141 10.1056/NEJMoa1609783 PMC5881572

[226] Grupp, S. A. et al. Chimeric antigen receptor-modified T cells for acute lymphoid leukemia. *N. Engl. J. Med.* **368**, 1509–1518 (2013). 23527958 10.1056/NEJMoa1215134 PMC4058440

[227] Cameron, B. J. et al. Identification of a Titin-derived HLA-A1-presented peptide as a cross-reactive target for engineered MAGE A3-directed T cells. *Sci. Transl. Med.* **5**, ra103 (2013). 10.1126/scitranslmed.3006034 PMC6002776 23926201

[228] Minotti, G., Menna, P., Salvatorelli, E., Cairo, G. & Gianni, L. Anthracyclines: molecular advances and pharmacologic developments in antitumor activity and cardiotoxicity. *Pharm. Rev.* **56**, 185–229 (2004). 15169927 10.1124/pr.56.2.6

[229] Bhagat, A., Shrestha, P. & Kleinerman, E. S. The innate immunesystem in cardiovascular diseases and Its role in doxorubicin-induced cardiotoxicity. *Int. J. Mol. Sci.* **23**, 14649 (2022). 36498974 10.3390/ijms232314649 PMC9739741

[230] Bayer, A. L. et al. Cytotoxic T cells drive doxorubicin-induced cardiac fibrosis and systolic dysfunction. *Nat. Cardiovasc. Res.* **3**, 970–986 (2024). 39196030 10.1038/s44161-024-00507-y PMC12324073

[231] Xi, Y. et al. Triptolide induces p53-dependent cardiotoxicity through mitochondrial membrane permeabilization in cardiomyocytes. *Toxicol. Appl. Pharmacol.* **355**, 269–285 (2018). 30009776 10.1016/j.taap.2018.07.011

[232] Wang, S. R. et al. MicroRNA expression, targeting, release dynamics and early-warning biomarkers in acute cardiotoxicity induced by triptolide in rats. *Biomed. Pharmacother.* **111**, 1467–1477 (2019). 30841462 10.1016/j.biopha.2018.12.109

[233] Dent, S. F., Morse, A., Burnette, S., Guha, A. & Moore, H. Cardiovascular Toxicity of Novel HER2-Targeted Therapies in the Treatment of Breast Cancer. *Curr. Oncol. Rep.* **23**, 128 (2021). 34453232 10.1007/s11912-021-01114-x PMC8395382

[234] Mahalingaiah, P. K. et al. Potential mechanisms of target-independent uptake and toxicity of antibody-drug conjugates. *Pharm. Ther.* **200**, 110–125 (2019). 10.1016/j.pharmthera.2019.04.008 31028836

[235] Ho, R. J. & Chien, J. Trends in translational medicine and drug targeting and delivery: new insights on an old concept-targeted drug delivery with antibody-drug conjugates for cancers. *J. Pharm. Sci.* **103**, 71–77 (2014). 24186148 10.1002/jps.23761 PMC4634700

[236] Behrens, G. M. & Reiss, P. Abacavir and cardiovascular risk. *Curr. Opin. Infect. Dis.* **23**, 9–14 (2010). 19996748 10.1097/QCO.0b013e328334fe84

[237] Fresse, A. et al. Spontaneous reported cardiotoxicity induced by lopinavir/ritonavir in COVID-19. An alleged past-resolved problem. *Int. J. Cardiol.* **324**, 255–260 (2021). 33075384 10.1016/j.ijcard.2020.10.028 PMC7566676

[238] Marzolini, C. et al. Effect of systemic inflammatory response to SARS-CoV-2 on lopinavir and hydroxychloroquine plasma concentrations. *Antimicrob. Agents Chemother.* **64**, e01177–01120 (2020). 32641296 10.1128/AAC.01177-20 PMC7449226

[239] Durrington, C. et al. Systematic pulmonary embolism follow-up increases diagnostic rates of chronic thromboembolic pulmonary hypertension and identifies less severe disease: results from the ASPIRE Registry. *Eur. Respir. J.* **63**, 2300846 (2024). 38302154 10.1183/13993003.00846-2023 PMC7615743

[240] Kang, Y. H. et al. Complement-coagulation cross-talk:factor H-mediated regulation of the complement classical pathway activation by fibrin clots. *Front. Immunol.* **15**, 1368852 (2024). 38933264 10.3389/fimmu.2024.1368852 PMC11199686

[241] Stark, K. & Massberg, S. Interplay between inflammation and thrombosis in cardiovascular pathology. *Nat. Rev. Cardiol.* **18**, 666–682 (2021). 33958774 10.1038/s41569-021-00552-1 PMC8100938

[242] Engelmann, B. & Massberg, S. Thrombosis as an intravascular effector of innate immunity. *Nat. Rev. Immunol.* **13**, 34–45 (2013). 23222502 10.1038/nri3345

[243] Renne, T. & Stavrou, E. X. Roles of factor XII in innate immunity. *Front. Immunol.* **10**, 2011 (2019). 31507606 10.3389/fimmu.2019.02011 PMC6713930

[244] Kale, S. et al. The effects of age on inflammatory and coagulation-fibrinolysis response in patients hospitalized for pneumonia. *PLoS ONE* **5**, e13852 (2010). 21085465 10.1371/journal.pone.0013852 PMC2973976

[245] Michels, E. H. A., et al. Association between age and the host response in critically ill patients with sepsis. *Crit. Care* **26**, 385 (2022). 36514130 10.1186/s13054-022-04266-9 PMC9747080

[246] Jackson, S. P. Arterial thrombosis-insidious, unpredictable and deadly. *Nat. Med.* **17**, 1423–1436 (2011). 22064432 10.1038/nm.2515

[247] Massberg, S. et al. A critical role of platelet adhesion in the initiation of atherosclerotic lesion formation. *J. Exp. Med.* **196**, 887–896 (2002). 12370251 10.1084/jem.20012044 PMC2194025

[248] Massberg, S. et al. Platelet adhesion via glycoprotein IIb integrin is critical for atheroprogression and focal cerebral ischemia: an in vivo study in mice lacking glycoprotein IIb. *Circulation* **112**, 1180–1188 (2005). 16103235 10.1161/CIRCULATIONAHA.105.539221

[249] Gerdes, N. et al. Platelet CD40 exacerbates atherosclerosis by transcellular activation of endothelial cells and leukocytes. *Arterioscler Thromb. Vasc. Biol.* **36**, 482–490 (2016). 26821950 10.1161/ATVBAHA.115.307074

[250] Drechsler, M. et al. Hyperlipidemia-triggered neutrophilia promotes early atherosclerosis. *Circulation* **122**, 1837–1845 (2010). 20956207 10.1161/CIRCULATIONAHA.110.961714

[251] Koenen, R. R. et al. Disrupting functional interactions between platelet chemokines inhibits atherosclerosis in hyperlipidemic mice. *Nat. Med.* **15**, 97–103 (2009). 19122657 10.1038/nm.1898

[252] Massberg, S. et al. Platelets secrete stromal cell-derived factor 1alpha and recruit bone marrow-derived progenitor cells to arterial thrombi in vivo. *J. Exp. Med.* **203**, 1221–1233 (2006). 16618794 10.1084/jem.20051772 PMC2121205

[253] Gaertner, F. et al. Migrating platelets are mechano-scavengers that collect and bundle bacteria. *Cell* **171**, 1368–1382.e1323 (2017). 29195076 10.1016/j.cell.2017.11.001

[254] Zuo, Y. et al. Neutrophil extracellular traps in COVID-19. *JCI Insight* **5**, e138999 (2020). 32329756 10.1172/jci.insight.138999 PMC7308057

[255] Martinod, K. et al. Neutrophil histone modification by peptidylarginine deiminase 4 is critical for deep vein thrombosis in mice. *Proc. Natl. Acad. Sci. USA* **110**, 8674–8679 (2013). 23650392 10.1073/pnas.1301059110 PMC3666755

[256] Li, P. et al. PAD4 is essential for antibacterial innate immunity mediated by neutrophil extracellular traps. *J. Exp. Med.* **207**, 1853–1862 (2010). 20733033 10.1084/jem.20100239 PMC2931169

[257] Stark, K. et al. Disulfide HMGB1 derived from platelets coordinates venous thrombosis in mice. *Blood* **128**, 2435–2449 (2016). 27574188 10.1182/blood-2016-04-710632 PMC5147023

[258] Jaen, R. I. et al. Innate immune receptors, key actors in cardiovascular diseases. *JACC Basic Transl. Sci.* **5**, 735–749 (2020). 32760860 10.1016/j.jacbts.2020.03.015 PMC7393405

[259] Fernandez-Ruiz, I. Immune system and cardiovascular disease. *Nat. Rev. Cardiol.* **13**, 503 (2016). 27516069 10.1038/nrcardio.2016.127

[260] Medzhitov, R., Preston-Hurlburt, P. & Janeway, C. A. Jr A human homologue of the Drosophila Toll protein signals activation of adaptive immunity. *Nature* **388**, 394–397 (1997). 9237759 10.1038/41131

[261] Liu, T., Liu, S. & Zhou, X. Innate immune responses and pulmonary diseases. *Adv. Exp. Med. Biol.* **1304**, 53–71 (2021). 34019263 10.1007/978-3-030-68748-9_4

[262] Ionita, M. G., Arslan, F., de Kleijn, D. P. & Pasterkamp, G. Endogenous inflammatory molecules engage Toll-like receptors in cardiovascular disease. *J. Innate Immun.* **2**, 307–315 (2010). 20431283 10.1159/000314270

[263] Steinberg, D. & Witztum, J. L. Oxidized low-density lipoprotein and atherosclerosis. *Arterioscler Thromb. Vasc. Biol.* **30**, 2311–2316 (2010). 21084697 10.1161/ATVBAHA.108.179697

[264] Akira, S., Uematsu, S. & Takeuchi, O. Pathogen recognition and innate immunity. *Cell* **124**, 783–801 (2006). 16497588 10.1016/j.cell.2006.02.015

[265] Lin, S. C., Lo, Y. C. & Wu, H. Helical assembly in the MyD88-IRAK4-IRAK2 complex in TLR/IL-1R signalling. *Nature* **465**, 885–890 (2010). 20485341 10.1038/nature09121 PMC2888693

[266] Motshwene, P. G. et al. An oligomeric signaling platform formed by the Toll-like receptor signal transducers MyD88 and IRAK-4. *J. Biol. Chem.* **284**, 25404–25411 (2009). 19592493 10.1074/jbc.M109.022392 PMC2757241

[267] Kollewe, C. et al. Sequential autophosphorylation steps in the interleukin-1 receptor-associated kinase-1 regulate its availability as an adapter in interleukin-1 signaling. *J. Biol. Chem.* **279**, 5227–5236 (2004). 14625308 10.1074/jbc.M309251200

[268] Verstak, B. et al. The TLR signaling adaptor TRAM interacts with TRAF6 to mediate activation of the inflammatory response by TLR4. *J. Leukoc. Biol.* **96**, 427–436 (2014). 24812060 10.1189/jlb.2A0913-487R PMC4632169

[269] Hu, L., et al. Oligomerization-primed coiled-coil domain interaction with Ubc13 confers processivity to TRAF6 ubiquitin ligase activity. *Nat. Commun.* **8**, 814 (2017). 28993672 10.1038/s41467-017-01290-0 PMC5634496

[270] Fitzgerald, K. A. & Kagan, J. C. Toll-like receptors and the control of immunity. *Cell* **180**, 1044–1066 (2020). 32164908 10.1016/j.cell.2020.02.041 PMC9358771

[271] McWhirter, S. M. et al. IFN-regulatory factor 3-dependent gene expression is defective in Tbk1-deficient mouse embryonic fibroblasts. *Proc. Natl. Acad. Sci. USA* **101**, 233–238 (2004). 14679297 10.1073/pnas.2237236100 PMC314168

[272] Sharma, S. et al. Triggering the interferon antiviral response through an IKK-related pathway. *Science* **300**, 1148–1151 (2003). 12702806 10.1126/science.1081315

[273] Liu, T. et al. Gasdermin B, an asthma-susceptibility gene, promotes MAVS-TBK1 signalling and airway inflammation. *Eur. Respir. J.* **63**, 2301232 (2024). 38514093 10.1183/13993003.01232-2023 PMC11063620

[274] Nishimura, M. & Naito, S. Tissue-specific mRNA expression profiles of human toll-like receptors and related genes. *Biol. Pharmacol. Bull.* **28**, 886–892 (2005). 10.1248/bpb.28.886 15863899

[275] Liu, L. et al. Up-regulated TLR4 in cardiomyocytes exacerbates heart failure after long-term myocardial infarction. *J. Cell Mol. Med.* **19**, 2728–2740 (2015). 26290459 10.1111/jcmm.12659 PMC4687701

[276] Oyama, J. et al. Reduced myocardial ischemia-reperfusion injury in toll-like receptor 4-deficient mice. *Circulation* **109**, 784–789 (2004). 14970116 10.1161/01.CIR.0000112575.66565.84

[277] Shimamoto, A. et al. Inhibition of Toll-like receptor 4 with eritoran attenuates myocardial ischemia-reperfusion injury. *Circulation* **114**, I270–274 (2006). 16820585 10.1161/CIRCULATIONAHA.105.000901

[278] Shishido, T. et al. Toll-like receptor-2 modulates ventricular remodeling after myocardial infarction. *Circulation* **108**, 2905–2910 (2003). 14656915 10.1161/01.CIR.0000101921.93016.1C

[279] Frantz, S. et al. Toll4 (TLR4) expression in cardiac myocytes in normal and failing myocardium. *J. Clin. Investig.* **104**, 271–280 (1999). 10430608 10.1172/JCI6709 PMC408420

[280] Liu, Y. Y. et al. Bacillus Calmette-Guérin and TLR4 agonist prevent cardiovascular hypertrophy and fibrosis by regulating immune microenvironment. *J. Immunol.* **180**, 7349–7357 (2008). 18490734 10.4049/jimmunol.180.11.7349

[281] Gao, W., Xiong, Y., Li, Q. & Yang, H. Inhibition of toll-like receptor signaling as a promising therapy for inflammatory diseases: a journey from molecular to nano therapeutics. *Front. Physiol.* **8**, 508 (2017). 28769820 10.3389/fphys.2017.00508 PMC5516312

[282] Yeh, F. C. et al. TLR7/8 activation induces autoimmune vasculopathy and causes severe pulmonary arterial hypertension. *Eur. Respir. J.* **62**, 2300204 (2023). 37290790 10.1183/13993003.00204-2023 PMC10356963

[283] Levitan, I., Volkov, S. & Subbaiah, P. V. Oxidized LDL: diversity, patterns of recognition, and pathophysiology. *Antioxid. Redox Signal.* **13**, 39–75 (2010). 19888833 10.1089/ars.2009.2733 PMC2877120

[284] Edfeldt, K., Swedenborg, J., Hansson, G. K. & Yan, Z. Q. Expression of toll-like receptors in human atherosclerotic lesions: a possible pathway for plaque activation. *Circulation* **105**, 1158–1161 (2002). 11889007

[285] Singh, R. K. et al. TLR4 (Toll-Like Receptor 4)-dependent signaling drives extracellular catabolism of LDL (Low-Density Lipoprotein) aggregates. *Arterioscler Thromb. Vasc. Biol.* **40**, 86–102 (2020). 31597445 10.1161/ATVBAHA.119.313200 PMC6928397

[286] Lee, G. L. et al. TLR2 promotes vascular smooth muscle cell chondrogenic differentiation and consequent calcification via the concerted actions of 0steoprotegerin suppression and IL-6-mediated RANKL induction. *Arterioscler Thromb. Vasc. Biol.* **39**, 432–445 (2019). 30626205 10.1161/ATVBAHA.118.311874

[287] Sundaram, B., Tweedell, R. E., Prasanth Kumar, S. & Kanneganti, T. D. The NLR family of innate immune and cell death sensors. *Immunity* **57**, 674–699 (2024). 38599165 10.1016/j.immuni.2024.03.012 PMC11112261

[288] Liu, T. et al. USP19 suppresses inflammation and promotes M2-like macrophage polarization by manipulating NLRP3 function via autophagy. *Cell Mol. Immunol.* **18**, 2431–2442 (2021). 33097834 10.1038/s41423-020-00567-7 PMC8484569

[289] Liu, T. et al. NOD-like receptor family, pyrin domain containing 3 (NLRP3) contributes to inflammation, pyroptosis, and mucin production in human airway epithelium on rhinovirus infection. *J. Allergy Clin. Immunol.* **144**, 777–787.e9 (2019). 31102698 10.1016/j.jaci.2019.05.006

[290] Sundaram, B. et al. NLRC5 senses NAD(+) depletion, forming a PANoptosome and driving PANoptosis and inflammation. *Cell* **187**, 4061–4077.E17 (2024). 38878777 10.1016/j.cell.2024.05.034 PMC11283362

[291] Sundaram, B. et al. NLRP12-PANoptosome activates PANoptosis and pathology in response to heme and PAMPs. *Cell* **186**, 2783–2801.e20 (2023). 37267949 10.1016/j.cell.2023.05.005 PMC10330523

[292] Nakamura, N. et al. Endosomes are specialized platforms for bacterial sensing and NOD2 signalling. *Nature* **509**, 240–244 (2014). 24695226 10.1038/nature13133

[293] Girardin, S. E. et al. Nod1 detects a unique muropeptide from gram-negative bacterial peptidoglycan. *Science* **300**, 1584–1587 (2003). 12791997 10.1126/science.1084677

[294] Girardin, S. E. et al. Nod2 is a general sensor of peptidoglycan through muramyl dipeptide (MDP) detection. *J. Biol. Chem.* **278**, 8869–8872 (2003). 12527755 10.1074/jbc.C200651200

[295] Caruso, R., Warner, N., Inohara, N. & Núñez, G. NOD1 and NOD2: signaling, host defense, and inflammatory disease. *Immunity* **41**, 898–908 (2014). 25526305 10.1016/j.immuni.2014.12.010 PMC4272446

[296] Inohara, N. et al. Nod1, an Apaf-1-like activator of caspase-9 and nuclear factor-kappaB. *J. Biol. Chem.* **274**, 14560–14567 (1999). 10329646 10.1074/jbc.274.21.14560

[297] Ogura, Y. et al. Nod2, a Nod1/Apaf-1 family member that is restricted to monocytes and activates NF-kappaB. *J. Biol. Chem.* **276**, 4812–4818 (2001). 11087742 10.1074/jbc.M008072200

[298] Sorbara, M. T. et al. The protein ATG16L1 suppresses inflammatory cytokines induced by the intracellular sensors Nod1 and Nod2 in an autophagy-independent manner. *Immunity* **39**, 858–873 (2013). 24238340 10.1016/j.immuni.2013.10.013

[299] Yeretssian, G. et al. Non-apoptotic role of BID in inflammation and innate immunity. *Nature* **474**, 96–99 (2011). 21552281 10.1038/nature09982

[300] Alvarez-Simon, D. et al. Local Receptor-interacting Protein Kinase 2 inhibition mitigates HDM-induced asthma. *Eur. Respir. J.* **64**, 2302288 (2024). 39117431 10.1183/13993003.02288-2023

[301] Watanabe, T. et al. NOD1 contributes to mouse host defense against Helicobacter pylori via induction of type I IFN and activation of the ISGF3 signaling pathway. *J. Clin. Investig.* **120**, 1645–1662 (2010). 20389019 10.1172/JCI39481 PMC2860924

[302] Bauernfeind, F. G. et al. Cutting edge: NF-kappaB activating pattern recognition and cytokine receptors license NLRP3 inflammasome activation by regulating NLRP3 expression. *J. Immunol.* **183**, 787–791 (2009). 19570822 10.4049/jimmunol.0901363 PMC2824855

[303] Xing, Y. et al. Cutting edge: TRAF6 mediates TLR/IL-1R signaling-induced nontranscriptional priming of the NLRP3 inflammasome. *J. Immunol.* **199**, 1561–1566 (2017). 28739881 10.4049/jimmunol.1700175

[304] Juliana, C. et al. Non-transcriptional priming and deubiquitination regulate NLRP3 inflammasome activation. *J. Biol. Chem.* **287**, 36617–36622 (2012). 22948162 10.1074/jbc.M112.407130 PMC3476327

[305] Py, B. F., Kim, M. S., Vakifahmetoglu-Norberg, H. & Yuan, J. Deubiquitination of NLRP3 by BRCC3 critically regulates inflammasome activity. *Mol. Cell* **49**, 331–338 (2013). 23246432 10.1016/j.molcel.2012.11.009

[306] Song, H., et al. The E3 ubiquitin ligase TRIM31 attenuates NLRP3 inflammasome activation by promoting proteasomal degradation of NLRP3. *Nat. Commun.* **7**, 13727 (2016). 27929086 10.1038/ncomms13727 PMC5155141

[307] Masumoto, J. et al. ASC, a novel 22-kDa protein, aggregates during apoptosis of human promyelocytic leukemia HL-60 cells. *J. Biol. Chem.* **274**, 33835–33838 (1999). 10567338 10.1074/jbc.274.48.33835

[308] Ramos-Junior, E. S. & Morandini, A. C. Gasdermin: a new player to the inflammasome game. *Biomed. J.* **40**, 313–316 (2017). 29433834 10.1016/j.bj.2017.10.002 PMC6138612

[309] He, Y. et al. NEK7 is an essential mediator of NLRP3 activation downstream of potassium efflux. *Nature* **530**, 354–357 (2016). 26814970 10.1038/nature16959 PMC4810788

[310] Shi, H. et al. NLRP3 activation and mitosis are mutually exclusive events coordinated by NEK7, a new inflammasome component. *Nat. Immunol.* **17**, 250–258 (2016). 26642356 10.1038/ni.3333 PMC4862588

[311] Zhou, L. et al. Excessive deubiquitination of NLRP3-R779C variant contributes to very-early-onset inflammatory bowel disease development. *J. Allergy Clin. Immunol.* **147**, 267–279 (2021). 32941940 10.1016/j.jaci.2020.09.003

[312] Liu, T., Woodruff, P. G. & Zhou, X. Advances in non-type 2 severe asthma: from molecular insights to novel treatment strategies. *Eur. Respir. J.* **64**, 2300826 (2024). 38697650 10.1183/13993003.00826-2023 PMC11325267

[313] Perea, L. et al. Airway IL-1β is related to disease severity and mucociliary function in bronchiectasis. *Eur. Respir. J.* **64**, 2301966 (2024). 38811046 10.1183/13993003.01966-2023

[314] Wu, C. H. et al. NLR network mediates immunity to diverse plant pathogens. *Proc. Natl. Acad. Sci. USA* **114**, 8113–8118 (2017). 28698366 10.1073/pnas.1702041114 PMC5544293

[315] Kuriakose, T. et al. ZBP1/DAI is an innate sensor of influenza virus triggering the NLRP3 inflammasome and programmed cell death pathways. *Sci. Immunol.* **1**, aag2045 (2016). 27917412 10.1126/sciimmunol.aag2045 PMC5131924

[316] Christgen, S. et al. Identification of the PANoptosome: a molecular platform triggering pyroptosis, apoptosis, and necroptosis (PANoptosis). *Front. Cell Infect. Microbiol* **10**, 237 (2020). 32547960 10.3389/fcimb.2020.00237 PMC7274033

[317] Yang, H. et al. Activation of NOD1 by DAP contributes to myocardial ischemia/reperfusion injury via multiple signaling pathways. *Apoptosis* **20**, 512–522 (2015). 25608996 10.1007/s10495-015-1089-1

[318] Val-Blasco, A. et al. NOD1 activation in cardiac fibroblasts induces myocardial fibrosis in a murine model of type 2 diabetes. *Biochem. J.* **474**, 399–410 (2017). 27803247 10.1042/BCJ20160556

[319] Shen, L. et al. Silencing of NOD2 protects against diabetic cardiomyopathy in a murine diabetes model. *Int. J. Mol. Med.* **42**, 3017–3026 (2018). 30221681 10.3892/ijmm.2018.3880 PMC6202090

[320] Zong, J. et al. NOD2 deletion promotes cardiac hypertrophy and fibrosis induced by pressure overload. *Lab Investig.* **93**, 1128–1136 (2013). 23958879 10.1038/labinvest.2013.99

[321] Kanno, S. et al. Activation of an innate immune receptor, Nod1, accelerates atherogenesis in Apoe-/- mice. *J. Immunol.* **194**, 773–780 (2015). 25488987 10.4049/jimmunol.1302841

[322] Johansson, M. E. et al. Innate immune receptor NOD2 promotes vascular inflammation and formation of lipid-rich necrotic cores in hypercholesterolemic mice. *Eur. J. Immunol.* **44**, 3081–3092 (2014). 25042478 10.1002/eji.201444755

[323] Yuan, H. et al. Pivotal role of NOD2 in inflammatory processes affecting atherosclerosis and periodontal bone loss. *Proc. Natl. Acad. Sci. USA* **110**, E5059–E5068 (2013). 24324141 10.1073/pnas.1320862110 PMC3876260

[324] Liu, H. Q. et al. NOD2-mediated innate immune signaling regulates the eicosanoids in atherosclerosis. *Arterioscler Thromb. Vasc. Biol.* **33**, 2193–2201 (2013). 23868940 10.1161/ATVBAHA.113.301715

[325] Kim, H. et al. A novel crosstalk between TLR4- and NOD2-mediated signaling in the regulation of intestinal inflammation. *Sci. Rep.* **5**, 12018 (2015). 26153766 10.1038/srep12018 PMC4495563

[326] Kim, H. J. Role of nucleotide-binding and oligomerization domain 2 protein (NOD2) in the development of atherosclerosis. *Korean J. Physiol. Pharmacol.* **19**, 479–484 (2015). 26557013 10.4196/kjpp.2015.19.6.479 PMC4637349

[327] Liu, Y. et al. TXNIP mediates NLRP3 inflammasome activation in cardiac microvascular endothelial cells as a novel mechanism in myocardial ischemia/reperfusion injury. *Basic Res. Cardiol.* **109**, 415 (2014). 25015733 10.1007/s00395-014-0415-z

[328] van Hout, G. P. et al. The selective NLRP3-inflammasome inhibitor MCC950 reduces infarct size and preserves cardiac function in a pig model of myocardial infarction. *Eur. Heart J.* **38**, 828–836 (2017). 27432019 10.1093/eurheartj/ehw247

[329] Toldo, S. & Abbate, A. The role of the NLRP3 inflammasome and pyroptosis in cardiovascular diseases. *Nat. Rev. Cardiol.* **21**, 219–237 (2024). 37923829 10.1038/s41569-023-00946-3 PMC11550901

[330] Kawaguchi, M. et al. Inflammasome activation of cardiac fibroblasts is essential for myocardial ischemia/reperfusion injury. *Circulation* **123**, 594–604 (2011). 21282498 10.1161/CIRCULATIONAHA.110.982777

[331] Li, X. et al. TAK1 Activation by NLRP3 Deficiency Confers Cardioprotection Against Pressure Overload-Induced Cardiomyocyte Pyroptosis and Hypertrophy. *JACC Basic Transl. Sci.* **8**, 1555–1573 (2023). 38205342 10.1016/j.jacbts.2023.05.008 PMC10774584

[332] Li, J. et al. Landscape of RNA-binding proteins in diagnostic utility, immune cell infiltration and PANoptosis features of heart failure. *Front. Genet* **13**, 1004163 (2022). 36313471 10.3389/fgene.2022.1004163 PMC9614340

[333] Bi, Y., et al. FUNDC1 protects against doxorubicin-induced cardiomyocyte PANoptosis through stabilizing mtDNA via interaction with TUFM. *Cell Death Dis.* **13**, 1020 (2022). 36470869 10.1038/s41419-022-05460-x PMC9723119

[334] Duewell, P. et al. NLRP3 inflammasomes are required for atherogenesis and activated by cholesterol crystals. *Nature* **464**, 1357–1361 (2010). 20428172 10.1038/nature08938 PMC2946640

[335] Yin, R. et al. MicroRNA-155 promotes the ox-LDL-induced activation of NLRP3 inflammasomes via the ERK1/2 pathway in THP-1 macrophages and aggravates atherosclerosis in ApoE-/- mice. *Ann. Palliat. Med.* **8**, 676–689 (2019). 31865729 10.21037/apm.2019.10.11

[336] Li, W. et al. Humanin ameliorates free fatty acid-induced endothelial inflammation by suppressing the NLRP3 inflammasome. *ACS Omega* **5**, 22039–22045 (2020). 32923762 10.1021/acsomega.0c01778 PMC7482084

[337] Bai, Y. et al. circACTA2 inhibits NLRP3 inflammasome-mediated inflammation via interacting with NF-κB in vascular smooth muscle cells. *Cell Mol. Life Sci.* **80**, 229 (2023). 37498354 10.1007/s00018-023-04840-6 PMC10374705

[338] Guo, S. et al. Endothelial nucleotide-binding oligomerization domain-like receptor protein 3 inflammasome regulation in atherosclerosis. *Cardiovasc. Res.* **120**, 883–898 (2024). 38626254 10.1093/cvr/cvae071

[339] Batool, M., Kim, M. S. & Choi, S. Structural insights into the distinctive RNA recognition and therapeutic potentials of RIG-I-like receptors. *Med. Res. Rev.* **42**, 399–425 (2022). 34287999 10.1002/med.21845

[340] Ferrage, F. et al. Structure and dynamics of the second CARD of human RIG-I provide mechanistic insights into regulation of RIG-I activation. *Structure* **20**, 2048–2061 (2012). 23063562 10.1016/j.str.2012.09.003 PMC3625992

[341] Satoh, T. et al. LGP2 is a positive regulator of RIG-I- and MDA5-mediated antiviral responses. *Proc. Natl. Acad. Sci. USA* **107**, 1512–1517 (2010). 20080593 10.1073/pnas.0912986107 PMC2824407

[342] Tang, X. et al. PARP9 is overexpressed in human breast cancer and promotes cancer cell migration. *Oncol. Lett.* **16**, 4073–4077 (2018). 30128030 10.3892/ol.2018.9124 PMC6096171

[343] Camicia, R. et al. BAL1/ARTD9 represses the anti-proliferative and pro-apoptotic IFNgamma-STAT1-IRF1-p53 axis in diffuse large B-cell lymphoma. *J. Cell Sci.* **126**, 1969–1980 (2013). 23487038 10.1242/jcs.118174

[344] Iwata, H. et al. PARP9 and PARP14 cross-regulate macrophage activation via STAT1 ADP-ribosylation. *Nat. Commun.* **7**, 12849 (2016). 27796300 10.1038/ncomms12849 PMC5095532

[345] Xing, J., et al. Identification of poly(ADP-ribose) polymerase 9 (PARP9) as a noncanonical sensor for RNA virus in dendritic cells. *Nat. Commun.* **12**, 2681 (2021). 33976210 10.1038/s41467-021-23003-4 PMC8113569

[346] Hornung, V. et al. 5’-Triphosphate RNA is the ligand for RIG-I. *Science* **314**, 994–997 (2006). 17038590 10.1126/science.1132505

[347] Myong, S. et al. Cytosolic viral sensor RIG-I is a 5’-triphosphate-dependent translocase on double-stranded RNA. *Science* **323**, 1070–1074 (2009). 19119185 10.1126/science.1168352 PMC3567915

[348] Hayden, M. S. & Ghosh, S. Signaling to NF-kappaB. *Genes Dev.* **18**, 2195–2224 (2004). 15371334 10.1101/gad.1228704

[349] Zhang, E. et al. Mechanisms involved in controlling RNA virus-induced intestinal inflammation. *Cell Mol. Life Sci.* **79**, 313 (2022). 35604464 10.1007/s00018-022-04332-z PMC9125963

[350] Li, Z., Nguyen, T. T. & Valaperti, A. Human cardiac fibroblasts produce pro-inflammatory cytokines upon TLRs and RLRs stimulation. *Mol. Cell Biochem.* **476**, 3241–3252 (2021). 33881711 10.1007/s11010-021-04157-7 PMC8059428

[351] Yu, P. et al. The function, role and process of DDX58 in heart failure and human cancers. *Front. Oncol.* **12**, 911309 (2022). 35814394 10.3389/fonc.2022.911309 PMC9257035

[352] Imaizumi, T. et al. Expression of retinoic acid-inducible gene-I (RIG-I) in macrophages: possible involvement of RIG-I in atherosclerosis. *J. Atheroscler. Thromb.* **14**, 51–55 (2007). 17485888 10.5551/jat.14.51

[353] Xu, S., Jin, T. & Weng, J. Endothelial cells as a key cell type for innate immunity: a focused review on RIG-I signaling pathway. *Front. Immunol.* **13**, 951614 (2022). 35865527 10.3389/fimmu.2022.951614 PMC9294349

[354] Chen, N. et al. PARP9 affects myocardial function through TGF-beta/Smad axis and pirfenidone. *Biomol. Biomed.* **24**, 1199–1215 (2024). 39213416 10.17305/bb.2024.10246 PMC11379006

[355] Xiao, C. Y. et al. Poly(ADP-Ribose) polymerase promotes cardiac remodeling, contractile failure, and translocation of apoptosis-inducing factor in a murine experimental model of aortic banding and heart failure. *J. Pharmacol. Exp. Ther.* **312**, 891–898 (2005). 15523000 10.1124/jpet.104.077164

[356] Halmosi, R. et al. PARP inhibition and postinfarction myocardial remodeling. *Int. J. Cardiol.* **217**, S52–S59 (2016). 27392900 10.1016/j.ijcard.2016.06.223

[357] Erbel, C. et al. PARP inhibition in atherosclerosis and its effects on dendritic cells, T cells and auto-antibody levels. *Eur. J. Med. Res.* **16**, 367–374 (2011). 21813379 10.1186/2047-783X-16-8-367 PMC3351988

[358] Sun, Z. & Hornung, V. cGAS-STING signaling. *Curr. Biol.* **32**, R730–r734 (2022). 35820380 10.1016/j.cub.2022.05.027

[359] Hu, M. M. & Shu, H. B. Mitochondrial DNA-triggered innate immune response: mechanisms and diseases. *Cell Mol. Immunol.* **20**, 1403–1412 (2023). 37932533 10.1038/s41423-023-01086-x PMC10687031

[360] Decout, A., Katz, J. D., Venkatraman, S. & Ablasser, A. The cGAS-STING pathway as a therapeutic target in inflammatory diseases. *Nat. Rev. Immunol.* **21**, 548–569 (2021). 33833439 10.1038/s41577-021-00524-z PMC8029610

[361] Zhang, Z. D. et al. Disulfiram ameliorates STING/MITA-dependent inflammation and autoimmunity by targeting RNF115. *Cell Mol. Immunol.* **21**, 275–291 (2024). 38267694 10.1038/s41423-024-01131-3 PMC10901794

[362] Liu, T. et al. The asthma risk gene, GSDMB, promotes mitochondrial DNA-induced ISGs expression. *J. Respir. Biol. Transl. Med.* **1**, 10005 (2024). 38737375 10.35534/jrbtm.2024.10005 PMC11086750

[363] Shang, G. et al. Cryo-EM structures of STING reveal its mechanism of activation by cyclic GMP-AMP. *Nature* **567**, 389–393 (2019). 30842659 10.1038/s41586-019-0998-5 PMC6859894

[364] Zhang, Z. et al. The helicase DDX41 senses intracellular DNA mediated by the adaptor STING in dendritic cells. *Nat. Immunol.* **12**, 959–965 (2011). 21892174 10.1038/ni.2091 PMC3671854

[365] Parvatiyar, K. et al. The helicase DDX41 recognizes the bacterial secondary messengers cyclic di-GMP and cyclic di-AMP to activate a type I interferon immune response. *Nat. Immunol.* **13**, 1155–1161 (2012). 23142775 10.1038/ni.2460 PMC3501571

[366] Motani, K., et al. The Golgi-resident protein ACBD3 concentrates STING at ER-Golgi contact sites to drive export from the ER. *Cell Rep.* **41**, 111868 (2022). 36543137 10.1016/j.celrep.2022.111868

[367] Sun, X. et al. Targeting STING in dendritic cells alleviates psoriatic inflammation by suppressing IL-17A production. *Cell Mol. Immunol.* **21**, 738–751 (2024). 38806624 10.1038/s41423-024-01160-y PMC11214627

[368] Ishikawa, H. & Barber, G. N. STING is an endoplasmic reticulum adaptor that facilitates innate immune signalling. *Nature* **455**, 674–678 (2008). 18724357 10.1038/nature07317 PMC2804933

[369] Tanaka, Y. & Chen, Z. J. STING specifies IRF3 phosphorylation by TBK1 in the cytosolic DNA signaling pathway. *Sci. Signal* **5**, ra20 (2012). 22394562 10.1126/scisignal.2002521 PMC3549669

[370] Lee, K. G. et al. Bruton’s tyrosine kinase phosphorylates DDX41 and activates its binding of dsDNA and STING to initiate type 1 interferon response. *Cell Rep.* **10**, 1055–1065 (2015). 25704810 10.1016/j.celrep.2015.01.039

[371] Cao, D. J. et al. Cytosolic DNA sensing promotes macrophage transformation and governs myocardial ischemic injury. *Circulation* **137**, 2613–2634 (2018). 29437120 10.1161/CIRCULATIONAHA.117.031046 PMC5997506

[372] Lai, L., et al. Plasmacytoid dendritic cells mediate myocardial ischemia/reperfusion injury by secreting type I interferons. *J. Am. Heart Assoc.* **10**, e020754 (2021). 34325534 10.1161/JAHA.121.020754 PMC8475660

[373] Hu, D. et al. Cytosolic DNA sensor cGAS plays an essential pathogenetic role in pressure overload-induced heart failure. *Am. J. Physiol. Heart Circ. Physiol.* **318**, H1525–h1537 (2020). 32383996 10.1152/ajpheart.00097.2020

[374] Luo, W. et al. Critical role of the cGAS-STING pathway in doxorubicin-induced cardiotoxicity. *Circ. Res.* **132**, e223–e242 (2023). 37154056 10.1161/CIRCRESAHA.122.321587

[375] Hayashi, C. et al. Porphyromonas gingivalis accelerates inflammatory atherosclerosis in the innominate artery of ApoE deficient mice. *Atherosclerosis* **215**, 52–59 (2011). 21251656 10.1016/j.atherosclerosis.2010.12.009 PMC3057233

[376] Olejarz, W., Łacheta, D. & Kubiak-Tomaszewska, G. Matrix metalloproteinases as biomarkers of atherosclerotic plaque instability. *Int. J. Mol. Sci*. **21**, 3946 (2020). 10.3390/ijms21113946 PMC7313469 32486345

[377] Pham, P. T. et al. STING, a cytosolic DNA sensor, plays a critical role in atherogenesis: a link between innate immunity and chronic inflammation caused by lifestyle-related diseases. *Eur. Heart J.* **42**, 4336–4348 (2021). 34226923 10.1093/eurheartj/ehab249

[378] Cai, D. et al. Balasubramide derivative 3C attenuates atherosclerosis in apolipoprotein E-deficient mice: role of AMPK-STAT1-STING signaling pathway. *Aging* **13**, 12160–12178 (2021). 33901014 10.18632/aging.202929 PMC8109080

[379] Kwak, H., Lee, E. & Karki, R. DNA sensors in metabolic and cardiovascular diseases: Molecular mechanisms and therapeutic prospects. *Immunol. Rev.* **329**, e13382 (2024). 39158380 10.1111/imr.13382 PMC11744256

[380] Venkat, V., et al. Investigating genes associated with heart failure, atrial fibrillation, and other cardiovascular diseases, and predicting disease using machine learning techniques for translational research and precision medicine. *Genomics* **115**, 110584 (2023). 36813091 10.1016/j.ygeno.2023.110584

[381] Smith, J. R., et al. MEF2A suppresses stress responses that trigger DDX41-dependent IFN production. *Cell Rep.* **42**, 112805 (2023). 37467105 10.1016/j.celrep.2023.112805 PMC10652867

[382] Liu, T. et al. TRIM11 suppresses AIM2 inflammasome by degrading AIM2 via p62-dependent selective autophagy. *Cell Rep.* **16**, 1988–2002 (2016). 27498865 10.1016/j.celrep.2016.07.019

[383] Baran, M., et al. PYHIN protein IFI207 regulates cytokine transcription and IRF7 and contributes to the establishment of K. pneumoniae infection. *Cell Rep.* **42**, 112341 (2023). 37018072 10.1016/j.celrep.2023.112341

[384] Yu, T. et al. TRIM11 attenuates Treg cell differentiation by p62-selective autophagic degradation of AIM2. *Cell Rep.* **42**, 113231 (2023). 37804507 10.1016/j.celrep.2023.113231

[385] Zheng, Y. et al. Zika virus elicits inflammation to evade antiviral response by cleaving cGAS via NS1-caspase-1 axis. *EMBO J.* **37**, e99347 (2018). 30065070 10.15252/embj.201899347 PMC6138430

[386] Hornung, V. et al. AIM2 recognizes cytosolic dsDNA and forms a caspase-1-activating inflammasome with ASC. *Nature* **458**, 514–518 (2009). 19158675 10.1038/nature07725 PMC2726264

[387] Fernandes-Alnemri, T. et al. AIM2 activates the inflammasome and cell death in response to cytoplasmic DNA. *Nature* **458**, 509–513 (2009). 19158676 10.1038/nature07710 PMC2862225

[388] Kerur, N. et al. IFI16 acts as a nuclear pathogen sensor to induce the inflammasome in response to Kaposi Sarcoma-associated herpesvirus infection. *Cell Host Microbe* **9**, 363–375 (2011). 21575908 10.1016/j.chom.2011.04.008 PMC3113467

[389] Fidler, T. P. et al. The AIM2 inflammasome exacerbates atherosclerosis in clonal haematopoiesis. *Nature* **592**, 296–301 (2021). 33731931 10.1038/s41586-021-03341-5 PMC8038646

[390] Onódi, Z. et al. AIM2-driven inflammasome activation in heart failure. *Cardiovasc. Res.* **117**, 2639–2651 (2021). 34117866 10.1093/cvr/cvab202

[391] Fahrländer, H. [Salazosulfapyridine in pregnancy]. *Dtsch Med. Wochenschr.* **103**, 1429 (1978). 28935

[392] Zhao, T. et al. Ginsenoside Rd promotes cardiac repair after myocardial infarction by modulating monocytes/macrophages subsets conversion. *Drug Des. Devel Ther.* **16**, 2767–2782 (2022). 36033133 10.2147/DDDT.S377624 PMC9416535

[393] Soehnlein, O. & Tall, A. R. AIMing 2 treat atherosclerosis. *Nat. Rev. Cardiol.* **19**, 567–568 (2022). 35882998 10.1038/s41569-022-00755-0 PMC9315844

[394] Lüsebrink, E., et al. AIM2 stimulation impairs reendothelialization and promotes the development of atherosclerosis in mice. *Front. Cardiovasc. Med.* **7**, 582482 (2020). 33263007 10.3389/fcvm.2020.582482 PMC7685997

[395] Pan, J. et al. AIM2 regulates vascular smooth muscle cell migration in atherosclerosis. *Biochem. Biophys. Res. Commun.* **497**, 401–409 (2018). 29448104 10.1016/j.bbrc.2018.02.094

[396] Paulin, N. et al. Double-strand DNA sensing Aim2 inflammasome regulates atherosclerotic plaque vulnerability. *Circulation* **138**, 321–323 (2018). 30012706 10.1161/CIRCULATIONAHA.117.033098

[397] Ugurlar, D. et al. Structures of C1-IgG1 provide insights into how danger pattern recognition activates complement. *Science* **359**, 794–797 (2018). 29449492 10.1126/science.aao4988

[398] Martin, M., Leffler, J. & Blom, A. M. Annexin A2 and A5 serve as new ligands for C1q on apoptotic cells. *J. Biol. Chem.* **287**, 33733–33744 (2012). 22879587 10.1074/jbc.M112.341339 PMC3460470

[399] Leffler, J. et al. Annexin-II, DNA, and histones serve as factor H ligands on the surface of apoptotic cells. *J. Biol. Chem.* **285**, 3766–3776 (2010). 19951950 10.1074/jbc.M109.045427 PMC2823518

[400] Mortensen, S. et al. Structural basis for the function of complement component C4 within the classical and lectin pathways of complement. *J. Immunol.* **194**, 5488–5496 (2015). 25911760 10.4049/jimmunol.1500087

[401] Sharp, T. H. et al. Insights into IgM-mediated complement activation based on in situ structures of IgM-C1-C4b. *Proc. Natl. Acad. Sci. USA* **116**, 11900–11905 (2019). 31147461 10.1073/pnas.1901841116 PMC6575175

[402] Zarantonello, A., Revel, M., Grunenwald, A. & Roumenina, L. T. C3-dependent effector functions of complement. *Immunol. Rev.* **313**, 120–138 (2023). 36271889 10.1111/imr.13147 PMC10092904

[403] Howard, M., Farrar, C. A. & Sacks, S. H. Structural and functional diversity of collectins and ficolins and their relationship to disease. *Semin. Immunopathol.* **40**, 75–85 (2018). 28894916 10.1007/s00281-017-0642-0 PMC5794833

[404] Fujita, T., Matsushita, M. & Endo, Y. The lectin-complement pathway-its role in innate immunity and evolution. *Immunol. Rev.* **198**, 185–202 (2004). 15199963 10.1111/j.0105-2896.2004.0123.x

[405] Fujita, T. Evolution of the lectin-complement pathway and its role in innate immunity. *Nat. Rev. Immunol.* **2**, 346–353 (2002). 12033740 10.1038/nri800

[406] Weis, W. I., Drickamer, K. & Hendrickson, W. A. Structure of a C-type mannose-binding protein complexed with an oligosaccharide. *Nature* **360**, 127–134 (1992). 1436090 10.1038/360127a0

[407] Hansen, S. et al. Collectin 11 (CL-11, CL-K1) is a MASP-1/3-associated plasma collectin with microbial-binding activity. *J. Immunol.* **185**, 6096–6104 (2010). 20956340 10.4049/jimmunol.1002185

[408] Jensen, M. L. et al. Ficolin-2 recognizes DNA and participates in the clearance of dying host cells. *Mol. Immunol.* **44**, 856–865 (2007). 16730064 10.1016/j.molimm.2006.04.002

[409] Choteau, L. et al. Role of mannose-binding lectin in intestinal homeostasis and fungal elimination. *Mucosal Immunol.* **9**, 767–776 (2016). 26442658 10.1038/mi.2015.100

[410] Medzhitov, R. & Janeway, C. A. Jr Decoding the patterns of self and nonself by the innate immune system. *Science* **296**, 298–300 (2002). 11951031 10.1126/science.1068883

[411] Turner, M. W. Mannose-binding lectin: the pluripotent molecule of the innate immune system. *Immunol. Today* **17**, 532–540 (1996). 8961631 10.1016/0167-5699(96)10062-1

[412] Wallis, R. Structural and functional aspects of complement activation by mannose-binding protein. *Immunobiology* **205**, 433–445 (2002). 12396005 10.1078/0171-2985-00144

[413] Wallis, R. Interactions between mannose-binding lectin and MASPs during complement activation by the lectin pathway. *Immunobiology* **212**, 289–299 (2007). 17544814 10.1016/j.imbio.2006.11.004 PMC2592599

[414] Ambrus, G. et al. Natural substrates and inhibitors of mannan-binding lectin-associated serine protease-1 and -2: a study on recombinant catalytic fragments. *J. Immunol.* **170**, 1374–1382 (2003). 12538697 10.4049/jimmunol.170.3.1374

[415] Gao, T. et al. Highly pathogenic coronavirus N protein aggravates inflammation by MASP-2-mediated lectin complement pathway overactivation. *Signal. Transduct. Target Ther.* **7**, 318 (2022). 36100602 10.1038/s41392-022-01133-5 PMC9470675

[416] Hallström, T. & Riesbeck, K. Haemophilus influenzae and the complement system. *Trends Microbiol.* **18**, 258–265 (2010). 20399102 10.1016/j.tim.2010.03.007

[417] de Boer, E. C. et al. The contribution of the alternative pathway in complement activation on cell surfaces depends on the strength of classical pathway initiation. *Clin. Transl. Immunol.* **12**, e1436 (2023). 10.1002/cti2.1436 PMC9881211 36721662

[418] Pangburn, M. K. Spontaneous reformation of the intramolecular thioester in complement protein C3 and low temperature capture of a conformational intermediate capable of reformation. *J. Biol. Chem.* **267**, 8584–8590 (1992). 1569104

[419] Pangburn, M. K., Schreiber, R. D. & Müller-Eberhard, H. J. Formation of the initial C3 convertase of the alternative complement pathway. Acquisition of C3b-like activities by spontaneous hydrolysis of the putative thioester in native C3. *J. Exp. Med.* **154**, 856–867 (1981). 6912277 10.1084/jem.154.3.856 PMC2186450

[420] Michels, M., Volokhina, E. B., van de Kar, N. & van den Heuvel, L. The role of properdin in complement-mediated renal diseases: a new player in complement-inhibiting therapy?. *Pediatr. Nephrol.* **34**, 1349–1367 (2019). 30141176 10.1007/s00467-018-4042-z PMC6579773

[421] Ricklin, D., Hajishengallis, G., Yang, K. & Lambris, J. D. Complement: a key system for immune surveillance and homeostasis. *Nat. Immunol.* **11**, 785–797 (2010). 20720586 10.1038/ni.1923 PMC2924908

[422] Verdeguer, F. et al. Complement regulation in murine and human hypercholesterolemia and role in the control of macrophage and smooth muscle cell proliferation. *Cardiovasc. Res.* **76**, 340–350 (2007). 17673191 10.1016/j.cardiores.2007.06.028

[423] Schepers, A. et al. Inhibition of complement component C3 reduces vein graft atherosclerosis in apolipoprotein E3-Leiden transgenic mice. *Circulation* **114**, 2831–2838 (2006). 17145993 10.1161/CIRCULATIONAHA.106.619502

[424] Wang, Y. et al. Clonally expanding smooth muscle cells promote atherosclerosis by escaping efferocytosis and activating the complement cascade. *Proc. Natl. Acad. Sci. USA* **117**, 15818–15826 (2020). 32541024 10.1073/pnas.2006348117 PMC7354942

[425] Hill, J. H. & Ward, P. A. The phlogistic role of C3 leukotactic fragments in myocardial infarcts of rats. *J. Exp. Med.* **133**, 885–900 (1971). 4993831 10.1084/jem.133.4.885 PMC2138969

[426] Nijmeijer, R. et al. C-reactive protein and complement depositions in human infarcted myocardium are more extensive in patients with reinfarction or upon treatment with reperfusion. *Eur. J. Clin. Investig.* **34**, 803–810 (2004). 15606722 10.1111/j.1365-2362.2004.01425.x

[427] Yasojima, K., Schwab, C., McGeer, E. G. & McGeer, P. L. Human heart generates complement proteins that are upregulated and activated after myocardial infarction. *Circ. Res.* **83**, 860–869 (1998). 9776733 10.1161/01.res.83.8.860

[428] Frey, A., et al. Complement C3c as a biomarker in heart failure. *Mediat. Inflamm.* **2013**, 716902 (2013). 10.1155/2013/716902 PMC3892932 24489446

[429] Nityanand, S. et al. Circulating immune complexes and complement C4 null alleles in patients in patients operated on for premature atherosclerotic peripheral vascular disease. *J. Clin. Immunol.* **19**, 406–413 (1999). 10634214 10.1023/a:1020506901117

[430] Bhatia, V. K. et al. Complement C1q reduces early atherosclerosis in low-density lipoprotein receptor-deficient mice. *Am. J. Pathol.* **170**, 416–426 (2007). 17200212 10.2353/ajpath.2007.060406 PMC1762701

[431] Jordan, J. E., Montalto, M. C. & Stahl, G. L. Inhibition of mannose-binding lectin reduces postischemic myocardial reperfusion injury. *Circulation* **104**, 1413–1418 (2001). 11560858 10.1161/hc3601.095578

[432] Walsh, M. C. et al. Mannose-binding lectin is a regulator of inflammation that accompanies myocardial ischemia and reperfusion injury. *J. Immunol.* **175**, 541–546 (2005). 15972690 10.4049/jimmunol.175.1.541

[433] Schwaeble, W. J. et al. Targeting of mannan-binding lectin-associated serine protease-2 confers protection from myocardial and gastrointestinal ischemia/reperfusion injury. *Proc. Natl. Acad. Sci. USA* **108**, 7523–7528 (2011). 21502512 10.1073/pnas.1101748108 PMC3088599

[434] Markiewski, M. M. & Lambris, J. D. The role of complement in inflammatory diseases from behind the scenes into the spotlight. *Am. J. Pathol.* **171**, 715–727 (2007). 17640961 10.2353/ajpath.2007.070166 PMC1959484

[435] Ritis, K. et al. A novel C5a receptor-tissue factor cross-talk in neutrophils links innate immunity to coagulation pathways. *J. Immunol.* **177**, 4794–4802 (2006). 16982920 10.4049/jimmunol.177.7.4794

[436] Delvaeye, M. & Conway, E. M. Coagulation and innate immune responses: can we view them separately?. *Blood* **114**, 2367–2374 (2009). 19584396 10.1182/blood-2009-05-199208

[437] Lam, N., Lee, Y. & Farber, D. L. A guide to adaptive immune memory. *Nat. Rev. Immunol.* **24**, 810–829 (2024). 38831162 10.1038/s41577-024-01040-6

[438] Chi, H., Pepper, M. & Thomas, P. G. Principles and therapeutic applications of adaptive immunity. *Cell* **187**, 2052–2078 (2024). 38670065 10.1016/j.cell.2024.03.037 PMC11177542

[439] Eiz-Vesper, B. & Schmetzer, H. M. Antigen-presenting cells: potential of proven and new players in immune therapies. *Transfus. Med. Hemother* **47**, 429–431 (2020). 33442337 10.1159/000512729 PMC7768096

[440] Joffre, O. P., Segura, E., Savina, A. & Amigorena, S. Cross-presentation by dendritic cells. *Nat. Rev. Immunol.* **12**, 557–569 (2012). 22790179 10.1038/nri3254

[441] Blum, J. S., Wearsch, P. A. & Cresswell, P. Pathways of antigen processing. *Annu. Rev. Immunol.* **31**, 443–473 (2013). 23298205 10.1146/annurev-immunol-032712-095910 PMC4026165

[442] Bonilla, F. A. & Oettgen, H. C. Adaptive immunity. *J. Allergy Clin. Immunol.* **125**, S33–S40 (2010). 20061006 10.1016/j.jaci.2009.09.017

[443] Wolf, D. & Ley, K. Immunity and inflammation in atherosclerosis. *Circ. Res.* **124**, 315–327 (2019). 30653442 10.1161/CIRCRESAHA.118.313591 PMC6342482

[444] Akinyemi, D. E., Chevre, R. & Soehnlein, O. Neuro-immune crosstalk in hematopoiesis, inflammation, and repair. *Trends Immunol.* **45**, 597–608 (2024). 39030115 10.1016/j.it.2024.06.005

[445] Barry, M. & Bleackley, R. C. Cytotoxic T lymphocytes: all roads lead to death. *Nat. Rev. Immunol.* **2**, 401–409 (2002). 12093006 10.1038/nri819

[446] Zhu, J. T Helper Cell Differentiation, Heterogeneity, and Plasticity. *Cold Spring Harb. Perspect. Biol.* **10**, a030338 (2018). 28847903 10.1101/cshperspect.a030338 PMC6169815

[447] Wigren, M., Nilsson, J. & Kolbus, D. Lymphocytes in atherosclerosis. *Clin. Chim. Acta* **413**, 1562–1568 (2012). 22565046 10.1016/j.cca.2012.04.031

[448] Kong, P. et al. Inflammation and atherosclerosis: signaling pathways and therapeutic intervention. *Signal. Transduct. Target Ther.* **7**, 131 (2022). 35459215 10.1038/s41392-022-00955-7 PMC9033871

[449] Shah, K., Al-Haidari, A., Sun, J. & Kazi, J. U. T cell receptor (TCR) signaling in health and disease. *Signal Transduct. Target Ther.* **6**, 412 (2021). 34897277 10.1038/s41392-021-00823-w PMC8666445

[450] Wucherpfennig, K. W. et al. Structural biology of the T-cell receptor: insights into receptor assembly, ligand recognition, and initiation of signaling. *Cold Spring Harb. Perspect. Biol.* **2**, a005140 (2010). 20452950 10.1101/cshperspect.a005140 PMC2845206

[451] Liew, F. Y. T(H)1 and T(H)2 cells: a historical perspective. *Nat. Rev. Immunol.* **2**, 55–60 (2002). 11905838 10.1038/nri705

[452] Coffman, R. L. Origins of the T(H)1-T(H)2 model: a personal perspective. *Nat. Immunol.* **7**, 539–541 (2006). 16715060 10.1038/ni0606-539

[453] Malissen, B., Grégoire, C., Malissen, M. & Roncagalli, R. Integrative biology of T cell activation. *Nat. Immunol.* **15**, 790–797 (2014). 25137453 10.1038/ni.2959

[454] Cantor, H. & Boyse, E. A. Functional subclasses of T-lymphocytes bearing different Ly antigens. I. The generation of functionally distinct T-cell subclasses is a differentiative process independent of antigen. *J. Exp. Med.* **141**, 1376–1389 (1975). 1092798 10.1084/jem.141.6.1376 PMC2189856

[455] Cerottini, J. C., Nordin, A. A. & Brunner, K. T. Specific in vitro cytotoxicity of thymus-derived lymphocytes sensitized to alloantigens. *Nature* **228**, 1308–1309 (1970). 4922690 10.1038/2281308a0

[456] Zhang, N. & Bevan, M. J. CD8(+) T cells: foot soldiers of the immune system. *Immunity* **35**, 161–168 (2011). 21867926 10.1016/j.immuni.2011.07.010 PMC3303224

[457] Yannelli, J. R., Sullivan, J. A., Mandell, G. L. & Engelhard, V. H. Reorientation and fusion of cytotoxic T lymphocyte granules after interaction with target cells as determined by high resolution cinemicrography. *J. Immunol.* **136**, 377–382 (1986). 3510248

[458] Trenn, G., Takayama, H. & Sitkovsky, M. V. Exocytosis of cytolytic granules may not be required for target cell lysis by cytotoxic T-lymphocytes. *Nature* **330**, 72–74 (1987). 3118213 10.1038/330072a0

[459] Boag, S. E. et al. T lymphocytes and fractalkine contribute to myocardial ischemia/reperfusion injury in patients. *J. Clin. Investig.* **125**, 3063–3076 (2015). 26168217 10.1172/JCI80055 PMC4563749

[460] Ilatovskaya, D. V. et al. CD8(+) T-cells negatively regulate inflammation post-myocardial infarction. *Am. J. Physiol. Heart Circ. Physiol.* **317**, H581–h596 (2019). 31322426 10.1152/ajpheart.00112.2019 PMC6766723

[461] Branchetti, E. et al. Oxidative stress modulates vascular smooth muscle cell phenotype via CTGF in thoracic aortic aneurysm. *Cardiovasc. Res.* **100**, 316–324 (2013). 23985903 10.1093/cvr/cvt205 PMC4192047

[462] Curato, C. et al. Identification of noncytotoxic and IL-10-producing CD8+AT2R+ T cell population in response to ischemic heart injury. *J. Immunol.* **185**, 6286–6293 (2010). 20935205 10.4049/jimmunol.0903681

[463] Elhage, R. et al. Deleting TCR alpha beta+ or CD4+ T lymphocytes leads to opposite effects on site-specific atherosclerosis in female apolipoprotein E-deficient mice. *Am. J. Pathol.* **165**, 2013–2018 (2004). 15579444 10.1016/s0002-9440(10)63252-x PMC1618721

[464] Sage, A. P. et al. X-box binding protein-1 dependent plasma cell responses limit the development of atherosclerosis. *Circ. Res.* **121**, 270–281 (2017). 28620068 10.1161/CIRCRESAHA.117.310884

[465] Taleb, S., Tedgui, A. & Mallat, Z. IL-17 and Th17 cells in atherosclerosis: subtle and contextual roles. *Arterioscler Thromb. Vasc. Biol.* **35**, 258–264 (2015). 25234818 10.1161/ATVBAHA.114.303567

[466] Weirather, J. et al. Foxp3+ CD4+ T cells improve healing after myocardial infarction by modulating monocyte/macrophage differentiation. *Circ. Res.* **115**, 55–67 (2014). 24786398 10.1161/CIRCRESAHA.115.303895

[467] Hofmann, U. et al. Activation of CD4+ T lymphocytes improves wound healing and survival after experimental myocardial infarction in mice. *Circulation* **125**, 1652–1663 (2012). 22388323 10.1161/CIRCULATIONAHA.111.044164

[468] Murphy, T. J. et al. CD4+CD25+ regulatory T cells control innate immune reactivity after injury. *J. Immunol.* **174**, 2957–2963 (2005). 15728508 10.4049/jimmunol.174.5.2957

[469] Jung, M. et al. IL-10 improves cardiac remodeling after myocardial infarction by stimulating M2 macrophage polarization and fibroblast activation. *Basic Res. Cardiol.* **112**, 33 (2017). 28439731 10.1007/s00395-017-0622-5 PMC5575998

[470] Ikeuchi, M. et al. Inhibition of TGF-beta signaling exacerbates early cardiac dysfunction but prevents late remodeling after infarction. *Cardiovasc. Res.* **64**, 526–535 (2004). 15537506 10.1016/j.cardiores.2004.07.017

[471] Akkaya, M., Kwak, K. & Pierce, S. K. B cell memory: building two walls of protection against pathogens. *Nat. Rev. Immunol.* **20**, 229–238 (2020). 31836872 10.1038/s41577-019-0244-2 PMC7223087

[472] Kwak, K., Akkaya, M. & Pierce, S. K. B cell signaling in context. *Nat. Immunol.* **20**, 963–969 (2019). 31285625 10.1038/s41590-019-0427-9

[473] Labeur-Iurman, L. & Harker, J. A. Mechanisms of antibody mediated immunity - Distinct in early life. *Int. J. Biochem. Cell Biol.* **172**, 106588 (2024). 38768890 10.1016/j.biocel.2024.106588

[474] Casadevall, A. & Pirofski, L. A. A new synthesis for antibody-mediated immunity. *Nat. Immunol.* **13**, 21–28 (2011). 22179281 10.1038/ni.2184 PMC3589717

[475] Hoehn, K. B., Fowler, A., Lunter, G. & Pybus, O. G. The diversity and molecular evolution of B-cell receptors during infection. *Mol. Biol. Evol.* **33**, 1147–1157 (2016). 26802217 10.1093/molbev/msw015 PMC4839220

[476] Sharma, R. et al. Distinct metabolic requirements regulate B cell activation and germinal center responses. *Nat. Immunol.* **24**, 1358–1369 (2023). 37365386 10.1038/s41590-023-01540-y PMC11262065

[477] Hägglöf, T. et al. Continuous germinal center invasion contributes to the diversity of the immune response. *Cell* **186**, 147–161.e115 (2023). 36565698 10.1016/j.cell.2022.11.032 PMC9825658

[478] Horckmans, M. et al. Pericardial adipose tissue regulates granulopoiesis, fibrosis, and cardiac function after myocardial infarction. *Circulation* **137**, 948–960 (2018). 29167227 10.1161/CIRCULATIONAHA.117.028833

[479] Wu, L. et al. IL-10-producing B cells are enriched in murine pericardial adipose tissues and ameliorate the outcome of acute myocardial infarction. *Proc. Natl. Acad. Sci. USA* **116**, 21673–21684 (2019). 31591231 10.1073/pnas.1911464116 PMC6815157

[480] Ma, S., Meng, Z., Chen, R. & Guan, K. L. The hippo pathway: biology and pathophysiology. *Annu. Rev. Biochem.* **88**, 577–604 (2019). 30566373 10.1146/annurev-biochem-013118-111829

[481] Yu, F. X., Zhao, B. & Guan, K. L. Hippo pathway in organ size control, tissue homeostasis, and cancer. *Cell* **163**, 811–828 (2015). 26544935 10.1016/j.cell.2015.10.044 PMC4638384

[482] Zhang, Q. et al. Hippo signalling governs cytosolic nucleic acid sensing through YAP/TAZ-mediated TBK1 blockade. *Nat. Cell Biol.* **19**, 362–374 (2017). 28346439 10.1038/ncb3496 PMC5398908

[483] Wang, S. et al. YAP antagonizes innate antiviral immunity and is targeted for lysosomal degradation through IKKvarepsilon-mediated phosphorylation. *Nat. Immunol.* **18**, 733–743 (2017). 28481329 10.1038/ni.3744

[484] Liu, B. et al. Toll receptor-mediated hippo signaling controls innate immunity in drosophila. *Cell* **164**, 406–419 (2016). 26824654 10.1016/j.cell.2015.12.029 PMC4733248

[485] Du, X. et al. Hippo/Mst signalling couples metabolic state and immune function of CD8alpha(+) dendritic cells. *Nature* **558**, 141–145 (2018). 29849151 10.1038/s41586-018-0177-0 PMC6292204

[486] Shi, H. et al. Hippo kinases Mst1 and Mst2 sense and amplify IL-2R-STAT5 signaling in regulatory T cells to establish stable regulatory activity. *Immunity* **49**, 899–914.e6 (2018). 30413360 10.1016/j.immuni.2018.10.010 PMC6249059

[487] Odashima, M. et al. Inhibition of endogenous Mst1 prevents apoptosis and cardiac dysfunction without affecting cardiac hypertrophy after myocardial infarction. *Circ. Res.* **100**, 1344–1352 (2007). 17395874 10.1161/01.RES.0000265846.23485.7a

[488] Del Re, D. P. et al. Mst1 promotes cardiac myocyte apoptosis through phosphorylation and inhibition of Bcl-xL. *Mol. Cell* **54**, 639–650 (2014). 24813943 10.1016/j.molcel.2014.04.007 PMC4074544

[489] Gao, Y. et al. YAP/TEAD1 complex is a default repressor of cardiac toll-like receptor genes. *Int. J. Mol. Sci.* **22**, 6649 (2021). 34206257 10.3390/ijms22136649 PMC8268263

[490] Wang, X. et al. TLR3 mediates repair and regeneration of damaged neonatal heart through glycolysis dependent YAP1 regulated miR-152 expression. *Cell Death Differ.* **25**, 966–982 (2018). 29358670 10.1038/s41418-017-0036-9 PMC5943401

[491] Lin, Z. et al. Cardiac-specific YAP activation improves cardiac function and survival in an experimental murine MI model. *Circ. Res.* **115**, 354–363 (2014). 24833660 10.1161/CIRCRESAHA.115.303632 PMC4104149

[492] Chen, J. et al. aYAP modRNA reduces cardiac inflammation and hypertrophy in a murine ischemia-reperfusion model. *Life Sci. Alliance* **3**, e201900424 (2020). 31843959 10.26508/lsa.201900424 PMC6918510

[493] Wang, P. et al. The alteration of Hippo/YAP signaling in the development of hypertrophic cardiomyopathy. *Basic Res. Cardiol.* **109**, 435 (2014). 25168380 10.1007/s00395-014-0435-8

[494] Xiong, Z. et al. Mst1 knockdown alleviates cardiac lipotoxicity and inhibits the development of diabetic cardiomyopathy in db/db mice. *Biochim. Biophys. Acta Mol. Basis Dis.* **1866**, 165806 (2020). 32320827 10.1016/j.bbadis.2020.165806

[495] Wang, K. C. et al. Flow-dependent YAP/TAZ activities regulate endothelial phenotypes and atherosclerosis. *Proc. Natl. Acad. Sci. USA* **113**, 11525–11530 (2016). 27671657 10.1073/pnas.1613121113 PMC5068257

[496] Wang, L. et al. Integrin-YAP/TAZ-JNK cascade mediates atheroprotective effect of unidirectional shear flow. *Nature* **540**, 579–582 (2016). 27926730 10.1038/nature20602

[497] Xu, Q. et al. Activation of yes-associated protein/PDZ-binding motif pathway contributes to endothelial dysfunction and vascular inflammation in angiotensinII hypertension. *Front. Physiol.* **12**, 732084 (2021). 34650444 10.3389/fphys.2021.732084 PMC8505766

[498] Niehrs, C. The complex world of WNT receptor signalling. *Nat. Rev. Mol. Cell Biol.* **13**, 767–779 (2012). 23151663 10.1038/nrm3470

[499] Liu, J. et al. Wnt/beta-catenin signalling: function, biological mechanisms, and therapeutic opportunities. *Signal. Transduct. Target Ther.* **7**, 3 (2022). 34980884 10.1038/s41392-021-00762-6 PMC8724284

[500] Nusse, R. & Clevers, H. Wnt/beta-catenin signaling, disease, and emerging therapeutic modalities. *Cell* **169**, 985–999 (2017). 28575679 10.1016/j.cell.2017.05.016

[501] Ma, B. & Hottiger, M. O. Crosstalk between Wnt/beta-catenin and NF-kappaB signaling pathway during inflammation. *Front. Immunol.* **7**, 378 (2016). 27713747 10.3389/fimmu.2016.00378 PMC5031610

[502] Trinath, J. et al. The WNT signaling pathway contributes to dectin-1-dependent inhibition of Toll-like receptor-induced inflammatory signature. *Mol. Cell Biol.* **34**, 4301–4314 (2014). 25246634 10.1128/MCB.00641-14 PMC4248746

[503] Aisagbonhi, O. et al. Experimental myocardial infarction triggers canonical Wnt signaling and endothelial-to-mesenchymal transition. *Dis. Model Mech.* **4**, 469–483 (2011). 21324930 10.1242/dmm.006510 PMC3124051

[504] Moon, J. et al. Blockade to pathological remodeling of infarcted heart tissue using a porcupine antagonist. *Proc. Natl. Acad. Sci. USA* **114**, 1649–1654 (2017). 28143939 10.1073/pnas.1621346114 PMC5320972

[505] Blumenthal, A. et al. The Wingless homolog WNT5A and its receptor Frizzled-5 regulate inflammatory responses of human mononuclear cells induced by microbial stimulation. *Blood* **108**, 965–973 (2006). 16601243 10.1182/blood-2005-12-5046

[506] Barandon, L. et al. Secreted frizzled-related protein-1 improves postinfarction scar formation through a modulation of inflammatory response. *Arterioscler Thromb. Vasc. Biol.* **31**, e80–e87 (2011). 21836067 10.1161/ATVBAHA.111.232280

[507] Lin, J. C. et al. beta-Catenin overexpression causes an increase in inflammatory cytokines and NF-kappaB activation in cardiomyocytes. *Cell Mol. Biol.* **63**, 17–22 (2016). 28234620 10.14715/cmb/2017.63.1.4

[508] van de Schans, V. A. et al. Interruption of Wnt signaling attenuates the onset of pressure overload-induced cardiac hypertrophy. *Hypertension* **49**, 473–480 (2007). 17210832 10.1161/01.HYP.0000255946.55091.24

[509] Awan, S. et al. Wnt5a promotes lysosomal cholesterol egress and protects against atherosclerosis. *Circ. Res.* **130**, 184–199 (2022). 34886684 10.1161/CIRCRESAHA.121.318881 PMC8776607

[510] Bhatt, P. M. & Malgor, R. Wnt5a: a player in the pathogenesis of atherosclerosis and other inflammatory disorders. *Atherosclerosis* **237**, 155–162 (2014). 25240110 10.1016/j.atherosclerosis.2014.08.027 PMC4252768

[511] Schaale, K. et al. Wnt signaling in macrophages: augmenting and inhibiting mycobacteria-induced inflammatory responses. *Eur. J. Cell Biol.* **90**, 553–559 (2011). 21185106 10.1016/j.ejcb.2010.11.004

[512] Borrell-Pages, M., Romero, J. C., Juan-Babot, O. & Badimon, L. Wnt pathway activation, cell migration, and lipid uptake is regulated by low-density lipoprotein receptor-related protein 5 in human macrophages. *Eur. Heart J.* **32**, 2841–2850 (2011). 21398644 10.1093/eurheartj/ehr062

[513] Wang, F. et al. Myeloid beta-catenin deficiency exacerbates atherosclerosis in low-density lipoprotein receptor-deficient mice. *Arterioscler Thromb. Vasc. Biol.* **38**, 1468–1478 (2018). 29724817 10.1161/ATVBAHA.118.311059 PMC6023740

[514] Schwartz, D. M. et al. JAK inhibition as a therapeutic strategy for immune and inflammatory diseases. *Nat. Rev. Drug Discov.* **16**, 843–862 (2017). 29104284 10.1038/nrd.2017.201

[515] O’Shea, J. J. et al. Janus kinase inhibitors in autoimmune diseases. *Ann. Rheum. Dis.* **72**, ii111–ii115 (2013). 23532440 10.1136/annrheumdis-2012-202576 PMC3616338

[516] Brooks, A. J. et al. Mechanism of activation of protein kinase JAK2 by the growth hormone receptor. *Science* **344**, 1249783 (2014). 24833397 10.1126/science.1249783

[517] Schwartz, D. M., Bonelli, M., Gadina, M. & O’Shea, J. J. Type I/II cytokines, JAKs, and new strategies for treating autoimmune diseases. *Nat. Rev. Rheumatol.* **12**, 25–36 (2016). 26633291 10.1038/nrrheum.2015.167 PMC4688091

[518] O’Shea, J. J., Holland, S. M. & Staudt, L. M. JAKs and STATs in immunity, immunodeficiency, and cancer. *N. Engl. J. Med.* **368**, 161–170 (2013). 23301733 10.1056/NEJMra1202117 PMC7604876

[519] Banerjee, S. et al. JAK-STAT signaling as a target for inflammatory and autoimmune diseases: current and future prospects. *Drugs* **77**, 521–546 (2017). 28255960 10.1007/s40265-017-0701-9 PMC7102286

[520] McCormick, J. et al. Free radical scavenging inhibits STAT phosphorylation following in vivo ischemia/reperfusion injury. *FASEB J.* **20**, 2115–2117 (2006). 16935931 10.1096/fj.06-6188fje

[521] Negoro, S. et al. Activation of JAK/STAT pathway transduces cytoprotective signal in rat acute myocardial infarction. *Cardiovasc. Res.* **47**, 797–805 (2000). 10974228 10.1016/s0008-6363(00)00138-3

[522] Kunisada, K. et al. Signal transducer and activator of transcription 3 in the heart transduces not only a hypertrophic signal but a protective signal against doxorubicin-induced cardiomyopathy. *Proc. Natl. Acad. Sci. USA* **97**, 315–319 (2000). 10618415 10.1073/pnas.97.1.315 PMC26660

[523] Hilfiker-Kleiner, D. et al. Signal transducer and activator of transcription 3 is required for myocardial capillary growth, control of interstitial matrix deposition, and heart protection from ischemic injury. *Circ. Res.* **95**, 187–195 (2004). 15192020 10.1161/01.RES.0000134921.50377.61

[524] Dawn, B. et al. IL-6 plays an obligatory role in late preconditioning via JAK-STAT signaling and upregulation of iNOS and COX-2. *Cardiovasc. Res.* **64**, 61–71 (2004). 15364614 10.1016/j.cardiores.2004.05.011 PMC3691700

[525] Xuan, Y. T. et al. Nuclear factor-kappaB plays an essential role in the late phase of ischemic preconditioning in conscious rabbits. *Circ. Res.* **84**, 1095–1109 (1999). 10325247 10.1161/01.res.84.9.1095

[526] Dotan, I. et al. Macrophage Jak2 deficiency accelerates atherosclerosis through defects in cholesterol efflux. *Commun. Biol.* **5**, 132 (2022). 35169231 10.1038/s42003-022-03078-5 PMC8847578

[527] An, H. J. et al. STAT3/NF‑kappaB decoy oligodeoxynucleotides inhibit atherosclerosis through regulation of the STAT/NF‑kappaB signaling pathway in a mouse model of atherosclerosis. *Int. J. Mol. Med.* **51**, 37 (2023). 37026512 10.3892/ijmm.2023.5240 PMC10094942

[528] Dor, Y. & Cedar, H. Principles of DNA methylation and their implications for biology and medicine. *Lancet* **392**, 777–786 (2018). 30100054 10.1016/S0140-6736(18)31268-6

[529] Moore, L. D., Le, T. & Fan, G. DNA methylation and its basic function. *Neuropsychopharmacology* **38**, 23–38 (2013). 22781841 10.1038/npp.2012.112 PMC3521964

[530] Ying, Z. et al. Enhanced CD19 activity in B cells contributes to immunodeficiency in mice deficient in the ICF syndrome gene Zbtb24. *Cell Mol. Immunol.* **20**, 1487–1498 (2023). 37990035 10.1038/s41423-023-01106-w PMC10687020

[531] Cardenas, A., Fadadu, R. & Bunyavanich, S. Climate change and epigenetic biomarkers in allergic and airway diseases. *J. Allergy Clin. Immunol.* **152**, 1060–1072 (2023). 37741554 10.1016/j.jaci.2023.09.011 PMC10843253

[532] Perez-Garcia, J., Cardenas, A., Lorenzo-Diaz, F. & Pino-Yanes, M. Precision medicine for asthma treatment: Unlocking the potential of the epigenome and microbiome. *J. Allergy Clin. Immunol.* **S0091-6749**, 00634–1 (2024). 10.1016/j.jaci.2024.06.010 PMC12002393 38906272

[533] Zheng, Y. et al. Association of cardiovascular health through young adulthood with genome-wide DNA methylation patterns in midlife: the CARDIA study. *Circulation* **146**, 94–109 (2022). 35652342 10.1161/CIRCULATIONAHA.121.055484 PMC9348746

[534] Kuznetsova, T., Prange, K. H. M., Glass, C. K. & de Winther, M. P. J. Transcriptional and epigenetic regulation of macrophages in atherosclerosis. *Nat. Rev. Cardiol.* **17**, 216–228 (2020). 31578516 10.1038/s41569-019-0265-3 PMC7770754

[535] Chang, C. P., Su, Y. C., Hu, C. W. & Lei, H. Y. TLR2-dependent selective autophagy regulates NF-κB lysosomal degradation in hepatoma-derived M2 macrophage differentiation. *Cell Death Differ.* **20**, 515–523 (2013). 23175187 10.1038/cdd.2012.146 PMC3569990

[536] Zhao, C. et al. DNA methyltransferase 1 deficiency improves macrophage motility and wound healing by ameliorating cholesterol accumulation. *NPJ Regen. Med.* **8**, 29 (2023). 37291182 10.1038/s41536-023-00306-2 PMC10250321

[537] Millán-Zambrano, G., Burton, A., Bannister, A. J. & Schneider, R. Histone post-translational modifications - cause and consequence of genome function. *Nat. Rev. Genet.* **23**, 563–580 (2022). 35338361 10.1038/s41576-022-00468-7

[538] Bannister, A. J. & Kouzarides, T. Regulation of chromatin by histone modifications. *Cell Res.* **21**, 381–395 (2011). 21321607 10.1038/cr.2011.22 PMC3193420

[539] Li, Y. et al. Id2 epigenetically controls CD8(+) T-cell exhaustion by disrupting the assembly of the Tcf3-LSD1 complex. *Cell Mol. Immunol.* **21**, 292–308 (2024). 38287103 10.1038/s41423-023-01118-6 PMC10902300

[540] Jacobs, M. M. E. et al. Trained immunity is regulated by T cell-induced CD40-TRAF6 signaling. *Cell Rep.* **43**, 114664 (2024). 39178113 10.1016/j.celrep.2024.114664 PMC11536040

[541] Wang, N. et al. Histone lactylation boosts reparative gene activation post-myocardial infarction. *Circ. Res.* **131**, 893–908 (2022). 36268709 10.1161/CIRCRESAHA.122.320488

[542] Lan, C. et al. Inhibition of DYRK1A, via histone modification, promotes cardiomyocyte cell cycle activation and cardiac repair after myocardial infarction. *EBioMedicine* **82**, 104139 (2022). 35810562 10.1016/j.ebiom.2022.104139 PMC9278077

[543] Zhang, S. et al. Targeting NPM1 epigenetically promotes postinfarction cardiac repair by reprogramming reparative macrophage metabolism. *Circulation* **149**, 1982–2001 (2024). 38390737 10.1161/CIRCULATIONAHA.123.065506 PMC11175795

[544] Hoeksema, M. A. et al. Targeting macrophage Histone deacetylase 3 stabilizes atherosclerotic lesions. *EMBO Mol. Med.* **6**, 1124–1132 (2014). 25007801 10.15252/emmm.201404170 PMC4197860

[545] Vlad, M. L. et al. Histone acetyltransferase-dependent pathways mediate upregulation of NADPH oxidase 5 in human macrophages under inflammatory conditions: a potential mechanism of reactive oxygen species overproduction in atherosclerosis. *Oxid. Med. Cell Longev.* **2019**, 3201062 (2019). 10.1155/2019/3201062 PMC6745143 31565149

[546] Gao, Y. et al. LNCGM1082-mediated NLRC4 activation drives resistance to bacterial infection. *Cell Mol. Immunol.* **20**, 475–488 (2023). 36941318 10.1038/s41423-023-00995-1 PMC10203293

[547] Kim, J., et al. An enhancer RNA recruits KMT2A to regulate transcription of Myb. *Cell Rep.* **43**, 114378 (2024). 38889007 10.1016/j.celrep.2024.114378 PMC11369905

[548] Lu, Y. et al. The NF-κB-responsive long noncoding RNA FIRRE regulates posttranscriptional regulation of inflammatory gene expression through interacting with hnRNPU. *J. Immunol.* **199**, 3571–3582 (2017). 28993514 10.4049/jimmunol.1700091 PMC5672816

[549] Li, J. et al. A novel piwi-interacting RNA associates with type 2-high asthma phenotypes. *J. Allergy Clin. Immunol.* **153**, 695–704 (2024). 38056635 10.1016/j.jaci.2023.10.032

[550] Nemeth, K., Bayraktar, R., Ferracin, M. & Calin, G. A. Non-coding RNAs in disease: from mechanisms to therapeutics. *Nat. Rev. Genet.* **25**, 211–232 (2024). 37968332 10.1038/s41576-023-00662-1

[551] Gomes, C. P. C. et al. Regulatory RNAs in heart failure. *Circulation* **141**, 313–328 (2020). 31986093 10.1161/CIRCULATIONAHA.119.042474 PMC7012349

[552] Han, Y., Ma, J., Wang, J. & Wang, L. Silencing of H19 inhibits the adipogenesis and inflammation response in ox-LDL-treated Raw264.7 cells by up-regulating miR-130b. *Mol. Immunol.* **93**, 107–114 (2018). 29172088 10.1016/j.molimm.2017.11.017

[553] Fasolo, F. et al. Long noncoding RNA MIAT controls advanced atherosclerotic lesion formation and plaque destabilization. *Circulation* **144**, 1567–1583 (2021). 34647815 10.1161/CIRCULATIONAHA.120.052023 PMC8570347

[554] Shin, J. J. et al. Roles of lncRNAs in NF-κB-mediated macrophage inflammation and their implications in the pathogenesis of human diseases. *Int. J. Mol. Sci*. **25**, 2670 (2024). 10.3390/ijms25052670 PMC10931578 38473915

[555] Cynn, E. et al. Human macrophage long intergenic noncoding RNA, SIMALR, suppresses inflammatory macrophage apoptosis via NTN1 (Netrin-1). *Arterioscler Thromb. Vasc. Biol.* **43**, 286–299 (2023). 36546321 10.1161/ATVBAHA.122.318353 PMC10162399

[556] Wang, S. et al. LncRNA CCRR attenuates postmyocardial infarction inflammatory response by inhibiting the TLR signalling pathway. *Can. J. Cardiol.* **40**, 710–725 (2024). 38081511 10.1016/j.cjca.2023.12.003

[557] Ramanujam, D. et al. MicroRNA-21-dependent macrophage-to-fibroblast signaling determines the cardiac response to pressure overload. *Circulation* **143**, 1513–1525 (2021). 33550817 10.1161/CIRCULATIONAHA.120.050682 PMC8032214

[558] Nosalski, R. et al. T-cell-derived miRNA-214 mediates perivascular fibrosis in hypertension. *Circ. Res.* **126**, 988–1003 (2020). 32065054 10.1161/CIRCRESAHA.119.315428 PMC7147427

[559] Li, H. et al. Circular RNA circRNA_000203 aggravates cardiac hypertrophy via suppressing miR-26b-5p and miR-140-3p binding to Gata4. *Cardiovasc. Res.* **116**, 1323–1334 (2020). 31397837 10.1093/cvr/cvz215 PMC7243276

[560] Wang, Y. et al. CircUbe3a from M2 macrophage-derived small extracellular vesicles mediates myocardial fibrosis after acute myocardial infarction. *Theranostics* **11**, 6315–6333 (2021). 33995660 10.7150/thno.52843 PMC8120198

[561] Liu, Y. et al. The RNA m(6)A demethylase ALKBH5 drives emergency granulopoiesis and neutrophil mobilization by upregulating G-CSFR expression. *Cell Mol. Immunol.* **21**, 6–18 (2024). 38114747 10.1038/s41423-023-01115-9 PMC10757716

[562] Li, B. et al. TMK4-mediated FIP37 phosphorylation regulates auxin-triggered N(6)-methyladenosine modification of auxin biosynthetic genes in Arabidopsis. *Cell Rep.* **43**, 114597 (2024). 39106180 10.1016/j.celrep.2024.114597

[563] Wang, C. et al. RNA modification in cardiovascular disease: implications for therapeutic interventions. *Signal Transduct. Target Ther.* **8**, 412 (2023). 37884527 10.1038/s41392-023-01638-7 PMC10603151

[564] Cui, L. et al. RNA modifications: importance in immune cell biology and related diseases. *Signal Transduct. Target Ther.* **7**, 334 (2022). 36138023 10.1038/s41392-022-01175-9 PMC9499983

[565] Han, D. & Xu, M. M. RNA modification in the immune system. *Annu. Rev. Immunol.* **41**, 73–98 (2023). 37126422 10.1146/annurev-immunol-101921-045401

[566] Wu, H. et al. Dendritic cells with METTL3 gene knockdown exhibit immature properties and prolong allograft survival. *Genes Immun.* **21**, 193–202 (2020). 32457372 10.1038/s41435-020-0099-3

[567] Li, Y. et al. Low RNA stability signifies increased post-transcriptional regulation of cell identity genes. *Nucleic Acids Res.* **51**, 6020–6038 (2023). 37125636 10.1093/nar/gkad300 PMC10325912

[568] Li, Q. et al. METTL3 (Methyltransferase Like 3)-dependent N6-methyladenosine modification on braf mRNA promotes macrophage inflammatory response and atherosclerosis in mice. *Arterioscler Thromb. Vasc. Biol.* **43**, 755–773 (2023). 36951060 10.1161/ATVBAHA.122.318451

[569] Jian, D. et al. METTL14 aggravates endothelial inflammation and atherosclerosis by increasing FOXO1 N6-methyladeosine modifications. *Theranostics* **10**, 8939–8956 (2020). 32802173 10.7150/thno.45178 PMC7415798

[570] Zheng, Y. et al. Mettl14 mediates the inflammatory response of macrophages in atherosclerosis through the NF-κB/IL-6 signaling pathway. *Cell Mol. Life Sci.* **79**, 311 (2022). 35598196 10.1007/s00018-022-04331-0 PMC9124663

[571] Zheng, P. F., et al. m6A regulator-mediated RNA methylation modification patterns are involved in the regulation of the immune microenvironment in ischaemic cardiomyopathy. *Sci. Rep.* **13**, 5904 (2023). 37041267 10.1038/s41598-023-32919-4 PMC10090050

[572] Wang, K. et al. HNEAP regulates necroptosis of cardiomyocytes by suppressing the m(5) C methylation of Atf7 mRNA. *Adv. Sci.* **10**, e2304329 (2023). 10.1002/advs.202304329 PMC10700171 37870216

[573] Hou, J., et al. TGM1/3-mediated transamidation of Exo70 promotes tumor metastasis upon LKB1 inactivation. *Cell Rep.* **43**, 114604 (2024). 39146185 10.1016/j.celrep.2024.114604

[574] Guo, J., Zheng, H. & Xiong, S. SENP6 restricts the IFN-I-induced signaling pathway and antiviral activity by deSUMOylating USP8. *Cell Mol. Immunol.* **21**, 892–904 (2024). 38906982 10.1038/s41423-024-01193-3 PMC11291505

[575] Liu, J., Qian, C. & Cao, X. Post-translational modification control of innate immunity. *Immunity* **45**, 15–30 (2016). 27438764 10.1016/j.immuni.2016.06.020

[576] Xia, B. & Zhao, J. Unraveling novel strategies: targeting Miz1 for degradation to enhance antiviral defense against influenza A virus. *J. Respir. Biol. Transl. Med.* **1**, 10009 (2024). 39086612 10.35534/jrbtm.2024.10009 PMC11290323

[577] Taleb, S. J. et al. Molecular regulation of transforming growth factor-β1-induced thioredoxin-interacting protein ubiquitination and proteasomal degradation in lung fibroblasts: implication in pulmonary fibrosis. *J. Respir. Biol. Transl. Med.* **1**, 10002 (2024). 38529321 10.35534/jrbtm.2024.10002 PMC10962057

[578] An, Z. et al. Neutrophil extracellular traps induced by IL-8 aggravate atherosclerosis via activation NF-κB signaling in macrophages. *Cell Cycle* **18**, 2928–2938 (2019). 31496351 10.1080/15384101.2019.1662678 PMC6791689

[579] Yang, B., et al. Macrophage DCLK1 promotes obesity-induced cardiomyopathy via activating RIP2/TAK1 signaling pathway. *Cell Death Dis.* **14**, 419 (2023). 37443105 10.1038/s41419-023-05960-4 PMC10345119

[580] Liu, M., et al. Macrophage K63-linked ubiquitination of YAP promotes its nuclear localization and exacerbates atherosclerosis. *Cell Rep.* **32**, 107990 (2020). 32755583 10.1016/j.celrep.2020.107990

[581] Chen, H., et al. The E3 ubiquitin ligase WWP2 regulates pro-fibrogenic monocyte infiltration and activity in heart fibrosis. *Nat. Commun.* **13**, 7375 (2022). 36450710 10.1038/s41467-022-34971-6 PMC9712659

[582] Yang, L. L., et al. E3 ubiquitin ligase RNF5 attenuates pathological cardiac hypertrophy through STING. *Cell Death Dis.* **13**, 889 (2022). 36270989 10.1038/s41419-022-05231-8 PMC9587004

[583] González-Amor, M. et al. Interferon-stimulated gene 15 pathway is a novel mediator of endothelial dysfunction and aneurysms development in angiotensin II infused mice through increased oxidative stress. *Cardiovasc. Res.* **118**, 3250–3268 (2022). 34672341 10.1093/cvr/cvab321 PMC9799052

[584] Zhou, W., et al. Local thiamet-G delivery by a thermosensitive hydrogel confers ischemic cardiac repair via myeloid M2-like activation in a STAT6 O-GlcNAcylation-dependent manner. *Int. Immunopharmacol.* **131**, 111883 (2024). 38503016 10.1016/j.intimp.2024.111883

[585] Venkatakrishnan, A. J. et al. Molecular signatures of G-protein-coupled receptors. *Nature* **494**, 185–194 (2013). 23407534 10.1038/nature11896

[586] Park, J. C. et al. Fine-tuning GPCR-mediated neuromodulation by biasing signaling through different G protein subunits. *Mol. Cell* **83**, 2540–2558.e2512 (2023). 37390816 10.1016/j.molcel.2023.06.006 PMC10527995

[587] Liu, K. et al. Structural basis of CXC chemokine receptor 2 activation and signalling. *Nature* **585**, 135–140 (2020). 32610344 10.1038/s41586-020-2492-5

[588] Bajpai, G. et al. Tissue resident CCR2- and CCR2+ cardiac macrophages differentially orchestrate monocyte recruitment and fate specification following myocardial injury. *Circ. Res.* **124**, 263–278 (2019). 30582448 10.1161/CIRCRESAHA.118.314028 PMC6626616

[589] Wong, N. R. et al. Resident cardiac macrophages mediate adaptive myocardial remodeling. *Immunity* **54**, 2072–2088.e2077 (2021). 34320366 10.1016/j.immuni.2021.07.003 PMC8446343

[590] Klein, K. R. et al. Decoy receptor CXCR7 modulates adrenomedullin-mediated cardiac and lymphatic vascular development. *Dev. cell* **30**, 528–540 (2014). 25203207 10.1016/j.devcel.2014.07.012 PMC4166507

[591] Lill, N. L. & Sever, N. I. Where EGF receptors transmit their signals. *Sci. Signal.* **5**, pe41 (2012). 23012653 10.1126/scisignal.2003341 PMC3507515

[592] Okyere, A. D. et al. Myeloid cell-specific deletion of epidermal growth factor receptor aggravates acute cardiac injury. *Clin. Sci.* **137**, 1513–1531 (2023). 10.1042/CS20230804 PMC10758753 37728308

[593] Korf-Klingebiel, M. et al. Myeloid-derived growth factor protects against pressure overload-induced heart failure by preserving sarco/endoplasmic reticulum Ca(2+)-ATPase expression in cardiomyocytes. *Circulation* **144**, 1227–1240 (2021). 34372689 10.1161/CIRCULATIONAHA.120.053365

[594] Zeboudj, L. et al. Selective EGF-receptor inhibition in CD4(+) T Cells Induces anergy and limits atherosclerosis. *J. Am. Coll. Cardiol.* **71**, 160–172 (2018). 29325640 10.1016/j.jacc.2017.10.084

[595] Jia, D. et al. Interleukin-35 promotes macrophage survival and improves wound healing after myocardial infarction in mice. *Circ. Res.* **124**, 1323–1336 (2019). 30832557 10.1161/CIRCRESAHA.118.314569

[596] Singla, B. et al. CD47 activation by thrombospondin-1 in lymphatic endothelial cells suppresses lymphangiogenesis and promotes atherosclerosis. *Arterioscler Thromb. Vasc. Biol.* **43**, 1234–1250 (2023). 37259865 10.1161/ATVBAHA.122.318904 PMC10281185

[597] Higashi, Y. et al. Insulin-like growth factor-1 receptor deficiency in macrophages accelerates atherosclerosis and induces an unstable plaque phenotype in apolipoprotein E-deficient mice. *Circulation* **133**, 2263–2278 (2016). 27154724 10.1161/CIRCULATIONAHA.116.021805 PMC4899151

[598] Meng, Z. et al. Cationic proteins from eosinophils bind bone morphogenetic protein receptors promoting vascular calcification and atherogenesis. *Eur. Heart J.* **44**, 2763–2783 (2023). 37279475 10.1093/eurheartj/ehad262 PMC10393071

[599] Chen, B. et al. Macrophage Smad3 protects the infarcted heart, stimulating phagocytosis and regulating inflammation. *Circ. Res.* **125**, 55–70 (2019). 31092129 10.1161/CIRCRESAHA.119.315069 PMC6681442

[600] Wang, Z. et al. Myocardial protection by heparin-based coacervate of FGF10. *Bioact. Mater.* **6**, 1867–1877 (2021). 33336117 10.1016/j.bioactmat.2020.12.002 PMC7732874

[601] Shi, S., et al. Role of oxidative stress and inflammation-related signaling pathways in doxorubicin-induced cardiomyopathy. *Cell Commun. Signal.* **21**, 61 (2023). 36918950 10.1186/s12964-023-01077-5 PMC10012797

[602] Cai, S. et al. Mitochondrial dysfunction in macrophages promotes inflammation and suppresses repair after myocardial infarction. *J. Clin. Invest*. **133**, e159498 (2023). 10.1172/JCI159498 PMC9927948 36480284

[603] Dikalova, A. E. et al. Mitochondrial deacetylase Sirt3 reduces vascular dysfunction and hypertension while Sirt3 depletion in essential hypertension is linked to vascular inflammation and oxidative stress. *Circ. Res.* **126**, 439–452 (2020). 31852393 10.1161/CIRCRESAHA.119.315767 PMC7035170

[604] Wang, Y. P. & Lei, Q. Y. Metabolite sensing and signaling in cell metabolism. *Signal. Transduct. Target Ther.* **3**, 30 (2018). 30416760 10.1038/s41392-018-0024-7 PMC6224561

[605] Bekkering, S. et al. Innate immune cell activation and epigenetic remodeling in symptomatic and asymptomatic atherosclerosis in humans in vivo. *Atherosclerosis* **254**, 228–236 (2016). 27764724 10.1016/j.atherosclerosis.2016.10.019

[606] Mouton, A. J., Li, X., Hall, M. E. & Hall, J. E. Obesity, hypertension, and cardiac dysfunction: novel roles of immunometabolism in macrophage activation and inflammation. *Circ. Res.* **126**, 789–806 (2020). 32163341 10.1161/CIRCRESAHA.119.312321 PMC7255054

[607] Shirai, T. et al. The glycolytic enzyme PKM2 bridges metabolic and inflammatory dysfunction in coronary artery disease. *J. Exp. Med.* **213**, 337–354 (2016). 26926996 10.1084/jem.20150900 PMC4813677

[608] Watanabe, R. et al. Glucose metabolism controls disease-specific signatures of macrophage effector functions. *JCI Insight* **3**, e123047 (2018). 30333306 10.1172/jci.insight.123047 PMC6237479

[609] Liu, T. Regulation of inflammasome by autophagy. *Adv. Exp. Med. Biol.* **1209**, 109–123 (2019). 31728867 10.1007/978-981-15-0606-2_7

[610] Del Re, D. P. et al. Fundamental mechanisms of regulated cell death and implications for heart disease. *Physiol. Rev.* **99**, 1765–1817 (2019). 31364924 10.1152/physrev.00022.2018 PMC6890986

[611] Linton, M. F. et al. Macrophage apoptosis and efferocytosis in the pathogenesis of atherosclerosis. *Circ. J.* **80**, 2259–2268 (2016). 27725526 10.1253/circj.CJ-16-0924 PMC5459487

[612] Kojima, Y. et al. CD47-blocking antibodies restore phagocytosis and prevent atherosclerosis. *Nature* **536**, 86–90 (2016). 27437576 10.1038/nature18935 PMC4980260

[613] Gerlach, B. D. et al. Efferocytosis induces macrophage proliferation to help resolve tissue injury. *Cell Metab.* **33**, 2445–2463.e2448 (2021). 34784501 10.1016/j.cmet.2021.10.015 PMC8665147

[614] Lv, J. J. et al. CD147 sparks atherosclerosis by driving M1 phenotype and impairing efferocytosis. *Circ. Res.* **134**, 165–185 (2024). 38166463 10.1161/CIRCRESAHA.123.323223

[615] Fang, S. et al. IRGM/Irgm1 facilitates macrophage apoptosis through ROS generation and MAPK signal transduction: Irgm1(+/-) mice display increases atherosclerotic plaque stability. *Theranostics* **11**, 9358–9375 (2021). 34646375 10.7150/thno.62797 PMC8490524

[616] Guntupalli, V. et al. Solute carrier family 26 member 4 (SLC26A4), a potential therapeutic target for asthma. *J. Respir. Biol. Transl. Med*. **1**, (2024). 10.35534/jrbtm.2024.10011 PMC11296660 39100210

[617] Tang, L., Yu, X., Zheng, Y. & Zhou, N. Inhibiting SLC26A4 reverses cardiac hypertrophy in H9C2 cells and in rats. *PeerJ* **8**, e8253 (2020). 31998553 10.7717/peerj.8253 PMC6979409

[618] Xu, X. et al. Macrophage migration inhibitory factor plays a permissive role in the maintenance of cardiac contractile function under starvation through regulation of autophagy. *Cardiovasc. Res.* **99**, 412–421 (2013). 23674514 10.1093/cvr/cvt116 PMC3732061

[619] Tang, Y. et al. Autophagy protects mitochondrial health in heart failure. *Heart Fail Rev.* **29**, 113–123 (2024). 37823952 10.1007/s10741-023-10354-x

[620] Chan, S. H. et al. SIRT1 inhibition causes oxidative stress and inflammation in patients with coronary artery disease. *Redox Biol.* **13**, 301–309 (2017). 28601780 10.1016/j.redox.2017.05.027 PMC5466584

[621] Administration., U. S. F. A. D. *What are biologics? Questions and answers*., https://www.fda.gov/about-fda/center-biologics-evaluation-and-research-cber/what-are-biologics-questions-and-answers (2018).

[622] Zhu, X. et al. A novel interleukin-2-based fusion molecule, HCW9302, differentially promotes regulatory T cell expansion to treat atherosclerosis in mice. *Front. Immunol.* **14**, e159498 (2023). 10.3389/fimmu.2023.1114802 PMC9907325 36761778

[623] De Maio, A. Extracellular heat shock proteins, cellular export vesicles, and the stress Observation System: a form of communication during injury, infection, and cell damage. It is never known how far a controversial finding will go! Dedicated to Ferruccio Ritossa. *Cell Stress Chaperones* **16**, 235–249 (2011). 20963644 10.1007/s12192-010-0236-4 PMC3077223

[624] Bochaton, T. et al. Heat shock protein 70 as a biomarker of clinical outcomes after STEMI. *J. Am. Coll. Cardiol.* **75**, 122–124 (2020). 31918818 10.1016/j.jacc.2019.10.044

[625] Chang, T. T., Yang, H. Y., Chen, C. & Chen, J. W. CCL4 Inhibition in Atherosclerosis: Effects on Plaque Stability, Endothelial Cell Adhesiveness, and Macrophages Activation. *Int. J. Mol. Sci.* **21**, 6567 (2020). 32911750 10.3390/ijms21186567 PMC7555143

[626] Nakahashi-Oda, C. et al. CD300a blockade enhances efferocytosis by infiltrating myeloid cells and ameliorates neuronal deficit after ischemic stroke. *Sci. Immunol.* **6**, eabe7915 (2021). 34652960 10.1126/sciimmunol.abe7915

[627] Wolf, D. et al. A ligand-specific blockade of the integrin Mac-1 selectively targets pathologic inflammation while maintaining protective host-defense. *Nat. Commun.* **9**, 525 (2018). 29410422 10.1038/s41467-018-02896-8 PMC5802769

[628] Gustafsson, K. et al. Clearing and replacing tissue-resident myeloid cells with an anti-CD45 antibody-drug conjugate. *Blood Adv.* **7**, 6964–6973 (2023). 37748049 10.1182/bloodadvances.2023010561 PMC10690556

[629] Shvedova, M. et al. c-Jun N-terminal kinases (JNKs) in myocardial and cerebral ischemia/reperfusion injury. *Front. Pharmacol.* **9**, 715 (2018). 30026697 10.3389/fphar.2018.00715 PMC6041399

[630] Zidar, N., Dolenc-Strazar, Z., Jeruc, J. & Stajer, D. Immunohistochemical expression of activated caspase-3 in human myocardial infarction. *Virchows Arch.* **448**, 75–79 (2006). 16205944 10.1007/s00428-005-0073-5

[631] Balsam, L. B., Kofidis, T. & Robbins, R. C. Caspase-3 inhibition preserves myocardial geometry and long-term function after infarction. *J. Surg. Res.* **124**, 194–200 (2005). 15820248 10.1016/j.jss.2004.09.017

[632] Seropian, I. M., Cassaglia, P., Miksztowicz, V. & González, G. E. Unraveling the role of galectin-3 in cardiac pathology and physiology. *Front. Physiol.* **14**, 1304735 (2023). 38170009 10.3389/fphys.2023.1304735 PMC10759241

[633] Li, M. et al. Value of galectin-3 in acute myocardial infarction. *Am. J. Cardiovasc. Drugs* **20**, 333–342 (2020). 31784887 10.1007/s40256-019-00387-9

[634] Frangogiannis, N. G. Targeting galectin-3 in myocardial infarction: a unique opportunity for biomarker-guided therapy. *Cardiovasc. Res.* **119**, 2495–2496 (2023). 37772841 10.1093/cvr/cvad156 PMC10676455

[635] Poznyak, A. V. et al. NADPH oxidases and their role in atherosclerosis. *Biomedicines* **8**, 206 (2020). 32664404 10.3390/biomedicines8070206 PMC7399834

[636] Zhang, Y., Murugesan, P., Huang, K. & Cai, H. NADPH oxidases and oxidase crosstalk in cardiovascular diseases: novel therapeutic targets. *Nat. Rev. Cardiol.* **17**, 170–194 (2020). 31591535 10.1038/s41569-019-0260-8 PMC7880919

[637] Ulleryd, M. A. et al. Stimulation of alpha 7 nicotinic acetylcholine receptor (α7nAChR) inhibits atherosclerosis via immunomodulatory effects on myeloid cells. *Atherosclerosis* **287**, 122–133 (2019). 31260875 10.1016/j.atherosclerosis.2019.06.903

[638] Garscha, U. et al. BRP-187: A potent inhibitor of leukotriene biosynthesis that acts through impeding the dynamic 5-lipoxygenase/5-lipoxygenase-activating protein (FLAP) complex assembly. *Biochem. Pharmacol.* **119**, 17–26 (2016). 27592027 10.1016/j.bcp.2016.08.023

[639] Abdel-Magid, A. F. Endothelial lipase inhibitors for the treatment of atherosclerosis and cardiovascular disorders. *ACS Med. Chem. Lett.* **4**, 1016–1017 (2013). 24900598 10.1021/ml400361q PMC4027247

[640] Yasuda, T. et al. Endothelial lipase is increased by inflammation and promotes LDL uptake in macrophages. *J. Atheroscler. Thromb.* **14**, 192–201 (2007). 17726294 10.5551/jat.e502

[641] Wald, D., Gupta, K., Lu, Y. & Moreton, S. Targeting leukocyte derived MPO in heart failure. *Blood* **130**, 3570 (2017).

[642] Nguyen, N. et al. Abstract 14871: APD588, a novel, selective S1P receptor modulator, regulates inflammatory responses and attenuates cardiac dysfunction following experimental myocardial infarction in mice. *Circulation* **142**, A14871–A14871 (2020).

[643] Phan, F. et al. Sphingosine-1-phosphate signalling in the heart: exploring emerging perspectives in cardiopathology. *FEBS Lett.* **598**, 2641–2655 (2024). 38965662 10.1002/1873-3468.14973

[644] Zhang, F. et al. Sphingosine 1-phosphate signaling contributes to cardiac inflammation, dysfunction, and remodeling following myocardial infarction. *Am. J. Physiol. Heart Circ. Physiol.* **310**, H250–H261 (2016). 26589326 10.1152/ajpheart.00372.2015

[645] Reitz, C. J. et al. SR9009 administered for one day after myocardial ischemia-reperfusion prevents heart failure in mice by targeting the cardiac inflammasome. *Commun. Biol.* **2**, 353 (2019). 31602405 10.1038/s42003-019-0595-z PMC6776554

[646] Wang, D. et al. GDF15: emerging biology and therapeutic applications for obesity and cardiometabolic disease. *Nat. Rev. Endocrinol.* **17**, 592–607 (2021). 34381196 10.1038/s41574-021-00529-7

[647] Toldo, S. & Abbate, A. The NLRP3 inflammasome in acute myocardial infarction. *Nat. Rev. Cardiol.* **15**, 203–214 (2018). 29143812 10.1038/nrcardio.2017.161

[648] Jia, L. et al. Methylation of FOXP3 in regulatory T cells is related to the severity of coronary artery disease. *Atherosclerosis* **228**, 346–352 (2013). 23566804 10.1016/j.atherosclerosis.2013.01.027

[649] Komal, S. et al. Epigenetic regulation of macrophage polarization in cardiovascular diseases. *Pharmacuticals* **16**, 141 (2023). 10.3390/ph16020141 PMC9963081 37259293

[650] Bansal, S. S. et al. Dysfunctional and proinflammatory regulatory T-lymphocytes are essential for adverse cardiac remodeling in ischemic cardiomyopathy. *Circulation* **139**, 206–221 (2019). 30586716 10.1161/CIRCULATIONAHA.118.036065 PMC6322956

[651] Lu, X. et al. Reactive oxygen species responsive multifunctional fusion extracellular nanovesicles: prospective treatments for acute heart transplant rejection. *Adv. Mater.* **36**, e2406758 (2024). 10.1002/adma.202406758 38949397

[652] Zhou, J., et al. Natural melanin/alginate hydrogels achieve cardiac repair through ROS scavenging and macrophage polarization. *Adv. Sci.* **8**, e2100505 (2021). 10.1002/advs.202100505 PMC8529445 34414693

[653] Ridker, P. M. et al. Low-dose methotrexate for the prevention of atherosclerotic events. *N. Engl. J. Med.* **380**, 752–762 (2019). 30415610 10.1056/NEJMoa1809798 PMC6587584

[654] Cormack, S. et al. Effect of ciclosporin on safety, lymphocyte kinetics and left ventricular remodelling in acute myocardial infarction. *Br. J. Clin. Pharmacol.* **86**, 1387–1397 (2020). 32067256 10.1111/bcp.14252 PMC7318996

[655] Ali, R. M. et al. Treatment of coronary drug-eluting stent restenosis by a sirolimus- or paclitaxel-coated balloon. *JACC Cardiovasc. Inter.* **12**, 558–566 (2019). 10.1016/j.jcin.2018.11.040 30898253

[656] Rodriguez, A. E. et al. Randomized comparison of cost-saving and effectiveness of oral rapamycin plus bare-metal stents with drug-eluting stents: three-year outcome from the randomized oral rapamycin in Argentina (ORAR) III trial. *Catheter Cardiovasc. Inter.* **80**, 385–394 (2012). 10.1002/ccd.23352 22109997

[657] Stähli, B. E. et al. Mammalian target of rapamycin inhibition in patients with ST-segment elevation myocardial infarction. *J. Am. Coll. Cardiol.* **80**, 1802–1814 (2022). 36049557 10.1016/j.jacc.2022.08.747

[658] El Sayed, H. et al. A randomized phase II study of Xilonix, a targeted therapy against interleukin 1α, for the prevention of superficial femoral artery restenosis after percutaneous revascularization. *J. Vasc. Surg.* **63**, 133–141.e131 (2016). 26433546 10.1016/j.jvs.2015.08.069

[659] Abbate, A. et al. Interleukin-1 blockade inhibits the acute inflammatory response in patients with ST-segment-elevation myocardial infarction. *J. Am. Heart Assoc.* **9**, e014941 (2020). 32122219 10.1161/JAHA.119.014941 PMC7335541

[660] Myachikova, V. Y. et al. Treatment of idiopathic recurrent pericarditis with goflikicept: phase II/III study results. *J. Am. Coll. Cardiol.* **82**, 30–40 (2023). 37380301 10.1016/j.jacc.2023.04.046

[661] Ridker, P. M. et al. IL-6 inhibition with ziltivekimab in patients at high atherosclerotic risk (RESCUE): a double-blind, randomised, placebo-controlled, phase 2 trial. *Lancet* **397**, 2060–2069 (2021). 34015342 10.1016/S0140-6736(21)00520-1

[662] Kleveland, O. et al. Effect of a single dose of the interleukin-6 receptor antagonist tocilizumab on inflammation and troponin T release in patients with non-ST-elevation myocardial infarction: a double-blind, randomized, placebo-controlled phase 2 trial. *Eur. Heart J.* **37**, 2406–2413 (2016). 27161611 10.1093/eurheartj/ehw171

[663] Broch, K. et al. Randomized trial of interleukin-6 receptor inhibition in patients with acute ST-segment elevation myocardial infarction. *J. Am. Coll. Cardiol.* **77**, 1845–1855 (2021). 33858620 10.1016/j.jacc.2021.02.049

[664] Meyer, M. A. S. et al. Treatment effects of interleukin-6 receptor antibodies for modulating the systemic inflammatory response after out-of-hospital cardiac arrest (The IMICA Trial): a double-blinded, placebo-controlled, single-center, randomized, clinical trial. *Circulation* **143**, 1841–1851 (2021). 33745292 10.1161/CIRCULATIONAHA.120.053318 PMC8104015

[665] Zhang, F. S. et al. Therapeutic potential of colchicine in cardiovascular medicine: a pharmacological review. *Acta Pharmacol. Sin.* **43**, 2173–2190 (2022). 35046517 10.1038/s41401-021-00835-w PMC8767044

[666] Klück, V. et al. Dapansutrile, an oral selective NLRP3 inflammasome inhibitor, for treatment of gout flares: an open-label, dose-adaptive, proof-of-concept, phase 2a trial. *Lancet Rheumatol.* **2**, e270–e280 (2020). 33005902 10.1016/s2665-9913(20)30065-5 PMC7523621

[667] Wohlford, G. F. et al. Phase 1B, randomized, double-blinded, dose escalation, single-center, repeat dose safety and pharmacodynamics study of the oral NLRP3 inhibitor dapansutrile in subjects with NYHA II-III systolic heart failure. *J. Cardiovasc. Pharmacol.* **77**, 49–60 (2020). 33235030 10.1097/FJC.0000000000000931 PMC7774821

[668] Lin, S. H. et al. Treatment with TNF-α inhibitor rectifies M1 macrophage polarization from blood CD14+ monocytes in patients with psoriasis independent of STAT1 and IRF-1 activation. *J. Dermatol. Sci.* **91**, 276–284 (2018). 29914850 10.1016/j.jdermsci.2018.05.009

[669] Colombo, A. et al. A double-blind randomised study to evaluate the efficacy and safety of bindarit in preventing coronary stent restenosis. *EuroIntervention* **12**, e1385–e1394 (2016). 26690313 10.4244/EIJY15M12_03

[670] Lam, C. S. P. et al. Myeloperoxidase inhibition in heart failure with preserved or mildly reduced ejection fraction: SATELLITE trial results. *J. Card. Fail* **30**, 104–110 (2024). 37072105 10.1016/j.cardfail.2023.04.003

[671] Hernández-Jiménez, M. et al. First-in-human phase I clinical trial of a TLR4-binding DNA aptamer, ApTOLL: Safety and pharmacokinetics in healthy volunteers. *Mol. Ther. Nucleic Acids* **28**, 124–135 (2022). 35402075 10.1016/j.omtn.2022.03.005 PMC8938885

[672] Willingham, S. B. et al. The CD47-signal regulatory protein alpha (SIRPa) interaction is a therapeutic target for human solid tumors. *Proc. Natl. Acad. Sci. USA* **109**, 6662–6667 (2012). 22451913 10.1073/pnas.1121623109 PMC3340046

[673] D’Amico, M. et al. Lipocortin 1 reduces myocardial ischemia-reperfusion injury by affecting local leukocyte recruitment. *Faseb J.* **14**, 1867–1869 (2000). 11023969 10.1096/fj.99-0602fje

[674] Ferraro, B. et al. Pro-angiogenic macrophage phenotype to promote myocardial repair. *J. Am. Coll. Cardiol.* **73**, 2990–3002 (2019). 31196457 10.1016/j.jacc.2019.03.503

[675] Chen, J. et al. The annexin-A1 mimetic RTP-026 promotes acute cardioprotection through modulation of immune cell activation. *Pharmacol. Res.* **198**, 107005 (2023). 37992916 10.1016/j.phrs.2023.107005

[676] Gelevski, D. et al. Safety and activity of anti-CD14 antibody IC14 (atibuclimab) in ALS: experience with expanded access protocol. *Muscle Nerve* **67**, 354–362 (2023). 36533976 10.1002/mus.27775

[677] Zhao, R. et al. Recent advances in CXCL12/CXCR4 antagonists and nano-based drug delivery systems for cancer therapy. *Pharmaceutics* **14**, 1541 (2022). 35893797 10.3390/pharmaceutics14081541 PMC9332179

[678] Means, C. K. & Brown, J. H. Sphingosine-1-phosphate receptor signalling in the heart. *Cardiovasc. Res.* **82**, 193–200 (2009). 19282351 10.1093/cvr/cvp086 PMC2721649

[679] Guo, Y., et al. The therapeutic potential of mesenchymal stem cells for cardiovascular diseases. *Cell Death Dis.* **11**, 349 (2020). 32393744 10.1038/s41419-020-2542-9 PMC7214402

[680] Hare, J. M. et al. A randomized, double-blind, placebo-controlled, dose-escalation study of intravenous adult human mesenchymal stem cells (prochymal) after acute myocardial infarction. *J. Am. Coll. Cardiol.* **54**, 2277–2286 (2009). 19958962 10.1016/j.jacc.2009.06.055 PMC3580848

[681] Lee, J. W. et al. A randomized, open-label, multicenter trial for the safety and efficacy of adult mesenchymal stem cells after acute myocardial infarction. *J. Korean Med. Sci.* **29**, 23–31 (2014). 24431901 10.3346/jkms.2014.29.1.23 PMC3890472

[682] Gao, L. R., et al. Intracoronary infusion of Wharton’s jelly-derived mesenchymal stem cells in acute myocardial infarction: double-blind, randomized controlled trial. *BMC Med.* **13**, 162 (2015). 26162993 10.1186/s12916-015-0399-z PMC4499169

[683] Qayyum, A. A. et al. Danish phase II trial using adipose tissue derived mesenchymal stromal cells for patients with ischaemic heart failure. *ESC Heart Fail* **10**, 1170–1183 (2023). 36638837 10.1002/ehf2.14281 PMC10053281

[684] Ikeda, M. et al. Immunomodulatory cell therapy using αGalCer-pulsed dendritic cells ameliorates heart failure in a murine dilated cardiomyopathy model. *Circ. Heart Fail* **15**, e009366 (2022). 36268712 10.1161/CIRCHEARTFAILURE.122.009366 PMC9760469

[685] Chullikana, A. et al. Randomized, double-blind, phase I/II study of intravenous allogeneic mesenchymal stromal cells in acute myocardial infarction. *Cytotherapy* **17**, 250–261 (2015). 25484310 10.1016/j.jcyt.2014.10.009

[686] Butler, J. et al. Intravenous allogeneic mesenchymal stem cells for nonischemic cardiomyopathy: safety and efficacy results of a phase II-A randomized trial. *Circ. Res.* **120**, 332–340 (2017). 27856497 10.1161/CIRCRESAHA.116.309717

[687] Florea, V. et al. Dose comparison study of allogeneic mesenchymal stem cells in patients with ischemic cardiomyopathy (The TRIDENT Study). *Circ. Res.* **121**, 1279–1290 (2017). 28923793 10.1161/CIRCRESAHA.117.311827 PMC8742223

[688] Bartolucci, J. et al. Safety and efficacy of the intravenous infusion of umbilical cord mesenchymal stem cells in patients with heart failure: a phase 1/2 randomized controlled trial (RIMECARD trial [randomized clinical trial of intravenous infusion umbilical cord mesenchymal stem cells on cardiopathy]). *Circ. Res.* **121**, 1192–1204 (2017). 28974553 10.1161/CIRCRESAHA.117.310712 PMC6372053

[689] Ulus, A. T. et al. Intramyocardial transplantation of umbilical cord mesenchymal stromal cells in chronic ischemic cardiomyopathy: a controlled, randomized clinical trial (HUC-HEART Trial). *Int. J. Stem Cells* **13**, 364–376 (2020). 32840230 10.15283/ijsc20075 PMC7691850

[690] Nidorf, S. M. et al. Colchicine in patients with chronic coronary disease. *N. Engl. J. Med.* **383**, 1838–1847 (2020). 32865380 10.1056/NEJMoa2021372

